# Rieske Oxygenase Catalyzed C–H Bond Functionalization Reactions in Chlorophyll *b* Biosynthesis

**DOI:** 10.1021/acscentsci.2c00058

**Published:** 2022-07-27

**Authors:** Jianxin Liu, Madison Knapp, Minshik Jo, Zerick Dill, Jennifer Bridwell-Rabb

**Affiliations:** Department of Chemistry, University of Michigan, Ann Arbor, Michigan 48109, United States

## Abstract

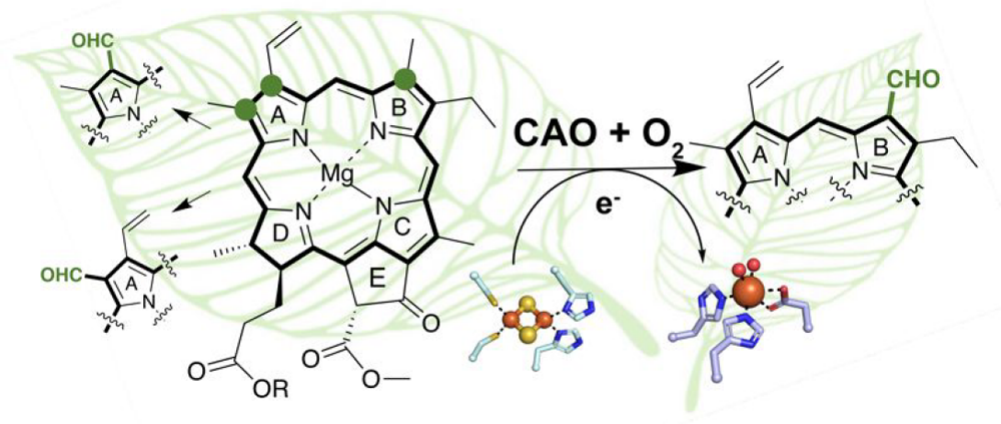

Rieske oxygenases perform precise C–H bond functionalization reactions in anabolic and catabolic pathways. These reactions are typically characterized as monooxygenation or dioxygenation reactions, but other divergent reactions are also catalyzed by Rieske oxygenases. Chlorophyll(ide) *a* oxygenase (CAO), for example is proposed to catalyze two monooxygenation reactions to transform a methyl-group into the formyl-group of Chlorophyll *b*. This formyl group, like the formyl groups found in other chlorophyll pigments, tunes the absorption spectra of chlorophyll*b* and supports the ability of several photosynthetic organisms to adapt to environmental light. Despite the importance of this reaction, CAO has never been studied in vitro with purified protein, leaving many open questions regarding whether CAO can facilitate both oxygenation reactions using just the Rieske oxygenase machinery. In this study, we demonstrated that four CAO homologues in partnership with a non-native reductase convert a Chlorophyll *a* precursor, chlorophyllide*a*, into chlorophyllide*b* in vitro. Analysis of this reaction confirmed the existence of the proposed intermediate, highlighted the stereospecificity of the reaction, and revealed the potential of CAO as a tool for synthesizing custom-tuned natural and unnatural chlorophyll pigments. This work thus adds to our fundamental understanding of chlorophyll biosynthesis and Rieske oxygenase chemistry.

## Abstract 2

Chlorophyll(ide) *a* oxygenase is a Rieske Oxygenase that can be used to formylate and custom-tune the absorption spectrum of various native and non-native chlorophyll scaffolds.

## Introduction

**Figure 1:**
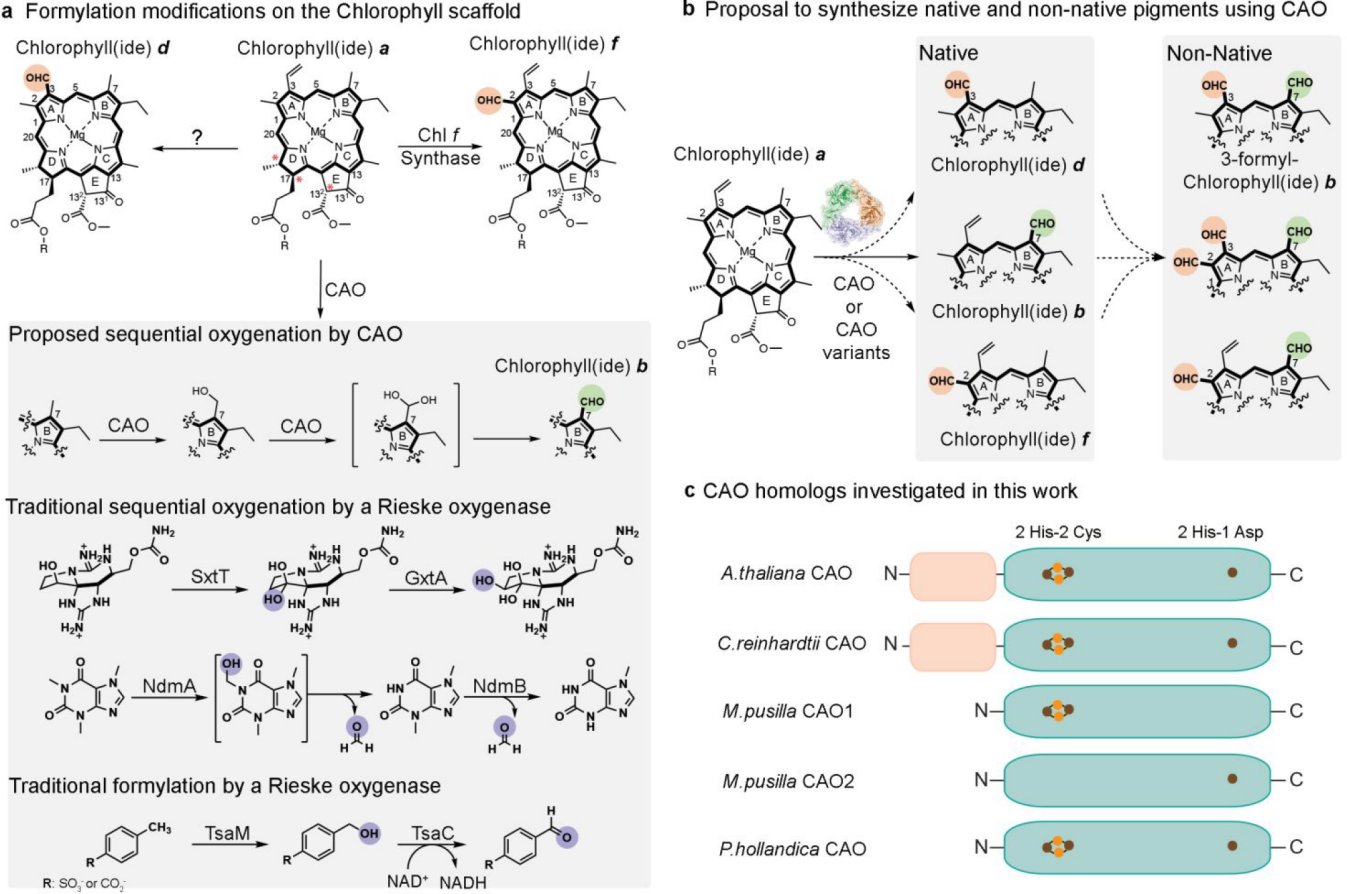
Formyl groups are abundant modifications made on the chlorophyll (Chl) scaffold. (a) The pigments Chl *b*, *d*, and *f* each bear formyl groups on their macrocyclic chlorin scaffolds at various positions. These modifications contribute to their characteristic absorbance patterns. The modifications found in Chl *d* and *f* are formed using an as-yet unidentified enzyme and the photooxidoreductase Chl *f* synthase,^1−3^ respectively. Formation of the C7 formyl group in chlorophyll(ide) *b* is instead proposed to be installed via two sequential chlorophyllide *a* oxygenase (CAO)-catalyzed reactions that transform the C7-methyl group of chlorophyll(ide) *a* into the formyl group of chlorophyll(ide) *b* through C7-hydroxymethyl and C7-dihydroxymethyl intermediates.^10^ All three chiral centers in Chl *a*, *b*, and intermediates are labeled with a red asterisk and for the chemical structure, R = H or C_20_H_39_ for chlorophyllide and chlorophyll pigments, respectively. This proposal for CAO is unlike that for other transformations that proceed through more than one monooxygenation reaction and require two Rieske oxygenases to be completed.^23−25^ This proposal is also unlike that needed to convert a methyl group into a formyl group in the catabolism of 4-toluene sulfonate, which requires both a Rieske oxygenase and dehydrogenase.^26^ (b) CAO has potential to be used as a tool for formylating the Chl scaffold to produce custom-tuned native and non-native pigments. (c) The CAO homologues studied in this work have different domain architectures. All homologues of CAO are predicted to have a Rieske [2Fe-2S] cluster and a mononuclear nonheme iron site in their catalytic domains (blue). These metallocenters are coordinated by two His and two Cys ligands and a facial triad of residues, respectively. The *Micromonas pusilla* CAO homologue is found in two polypeptide chains and the *Arabidopsis thaliana* and *Chlamydomonas reinhardtii* homologues have N-terminal regulatory domains (peach).^27^ As *Ph*CAO appears to contain the simplest architecture of all four homologues, it was the first CAO homologue characterized in this work.

Chlorophylls (Chls) are a class of naturally occurring pigments that play key roles in photosynthetic organisms. These pigments capture solar energy and facilitate its transformation into chemical energy.^1−4^ The diversity of this class of molecules, in some cases, arises from C–H bond functionalization reactions that decorate the macrocyclic scaffold and adjust its spectroscopic properties.^1−4^ One such scaffold modification that is found in Chl derivatives *b*, *d*, and *f*, is a formyl group^5^ (Figure 1a). These modifications have received attention for their ability to customize and extend the range of usable light in photosynthetic organisms.^5^ For example, Chl *d* and Chl *f*, allow photosynthetic microorganisms to absorb red and far-red light, which is typically found in soils, algal blooms, microbial mats, and other shaded environments.^6^ Chl *b*, on the other hand, is an integral accessory pigment that has enhanced absorption features for blue and green light.^3^ The enzyme involved in the biosynthesis of Chl *d* has yet to be identified and for Chl *f*, formylation is catalyzed by a membrane-bound photooxidoreductase (Figure 1a).^7−9^ Last, the formyl group of Chl *b* is installed by Chlorophyll(ide) *a* oxygenase (CAO), an annotated member of the large Rieske nonheme iron oxygenase, or Rieske oxygenase superfamily of enzymes (Figure 1a).^10,11^

Rieske oxygenases are classified by the presence of motifs for binding an N-terminal Rieske [2Fe-2S] cluster and a C-terminal mononuclear nonheme iron center. The Rieske cluster accepts external electrons from a dedicated reductase protein and shuttles them to the mononuclear iron site for activation of molecular oxygen (O_2_).^12−16^ The formed oxidant is then used to facilitate subsequent chemistry. Most characterized Rieske oxygenases have been shown to use their metallocenters to facilitate monooxygenation or dioxygenation events.^12−16^ CAO, however, is proposed to use the Rieske machinery to catalyze two sequential monooxygenation reactions and form both a hydroxymethyl and dihydroxymethyl intermediate.^10^ The latter species is then proposed to spontaneously lose water to become the formyl group found at the C7-position of Chl *b*(10) (Figure 1a). In support of this proposal, early in vivo biochemical characterization of CAO determined that the formyl group oxygen atom originates from O_2_.^17,18^ Other pioneering studies on the CAO homologue from *Arabidopsis thaliana* (*At*CAO) demonstrated that lysed cells, from which *At*CAO was recombinantly expressed, could facilitate the conversion of a small amount of the Chl precursor, Chlorophyllide (Chlide) *a*, into Chlide *b* when combined with a reducing system (Figure S1).^10^ This result, along with data that shows a Chl *b* deficiency can be traced to a single gene in multiple photosynthetic organisms suggests that CAO, in the absence of a dedicated partner protein, is sufficient to synthesize the formyl group.^10,19−21^

Therefore, CAO represents the seemingly simplest enzyme to formylate a pigment and has the potential to serve as a tool for catalyzing late-stage oxidation reactions at different positions on the pigment scaffold (Figure 1a and b). Remarkably, however, whether the substrate of this enzyme is Chl *a* or Chlide *a* is still in debate: no purification and in vitro reconstitution of a CAO homologue has been reported, and the activity of CAO has never been demonstrated in isolation.^4^ As this sequential oxygenation reaction represents a divergence from traditional Rieske oxygenase chemistry, many questions remain regarding whether the proposed sequential oxygenation reaction can occur in the absence of an additional component in vitro. For example, in other biosynthetic pathways where two oxygenation reactions are required, multiple proteins work together (Figure 1a). The two required oxygenations on the saxitoxin scaffold are made by the Rieske oxygenases SxtT and GxtA (Figure 1a).^22−24^ Likewise, two Rieske oxygenases, NdmA and NdmB, catalyze iterative oxidative demethylation reactions in caffeine degradation (Figure 1a).^25^ The Rieske oxygenase 4-toluenesulfonate methyl monooxygenase (TsaM) also catalyzes a methyl- to formyl-group transformation to convert 4-tolunesulfonate into 4-sulfobenzoate.^26^ Interestingly, TsaM requires the help of a nicotinamide adenine dinucleotide (NAD^+^)-dependent dehydrogenase (TsaC) to transform the monohydroxylated intermediate into the product (Figure 1a).^26^ In comparison to these reactions, however, it is possible that differences in the substrate structures or relative acidity of the C–H bonds at the site of functionalization allow CAO to use an alternative reaction path.

Thus, in this work, we designed methods to study four homologues of CAO from different kingdoms of life^27^ in vitro with purified protein (Figures 1c and S2). These homologues were chosen based on their predicted involvement in Chl *b* biosynthesis and their different annotated domain architectures (Figures 1c and S2). As CAO could potentially serve as a tool for synthesizing formylated Chl species that would otherwise be synthetically complicated to obtain,^28,29^ we sought to determine whether these homologues could catalyze the predicted formylation reaction in vitro. Through these studies, a non-native Rieske reductase was identified that could work in partnership with each CAO homologue to convert a Chl *a* precursor, Chlide *a*, into Chlide *b* in vitro. Analysis of this reaction using liquid chromatography mass spectrometry (LC-MS) confirmed that the CAO homologues could facilitate two oxygenation reactions in the absence of an additional Rieske oxygenase, dehydrogenase, or cofactor. In addition, these experiments confirmed the identity of the proposed monooxygenated intermediate and showed that it too can be synthesized and accepted by CAO as a substrate. Finally, this work revealed intriguing details regarding the stereoselectivity of this reaction, the substrate scope of CAO, and the potential for using CAO as an enzymatic tool for synthesizing custom Chl pigments, those of which could be utilized in the cosmetic, food, agricultural, and pharmaceutical industries.^30^

## Results

### Purification of Four Different CAO Homologues

**Figure 2:**
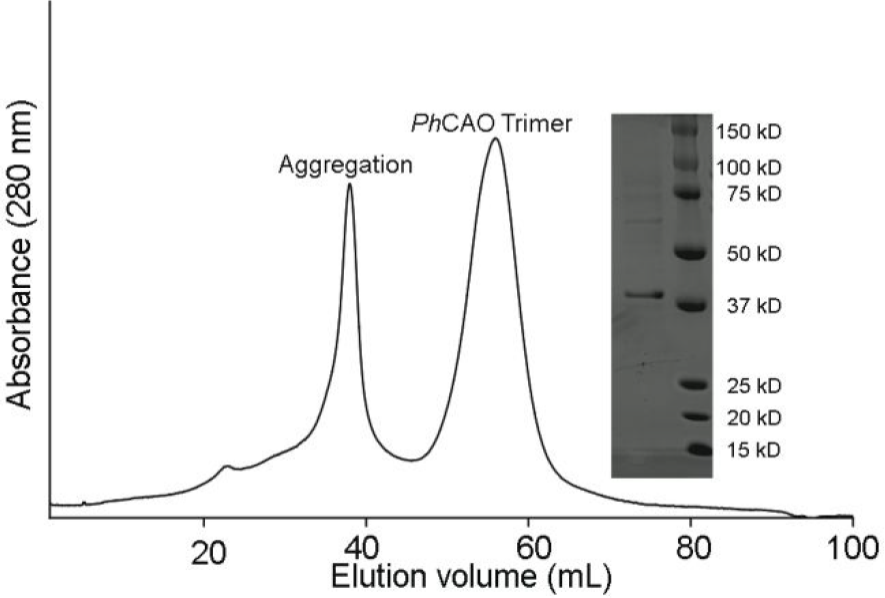
*P. hollandica* CAO can be purified and used for in vitro biochemical studies. A gel filtration profile of *Ph*CAO reveals that CAO purifies with the expected trimeric quaternary structure of a Rieske oxygenase. An inset of an SDS-PAGE gel reveals the purity of the isolated trimeric *Ph*CAO (expected monomeric molecular weight of 41 kDa).

To address the ability of CAO to form chlorophyll(ide) *b* in vitro, genes encoding the CAO homologues from the prokaryotic organism *Prochlorothrix hollandica* (*Ph*CAO), the model plant *Arabidopsis thaliana* (*At*CAO), and two green algae *Chlamydomonas reinhardtii* (*Cr*CAO) and *Micromonas pusilla* (*Mp*CAO), were synthesized and codon-optimized for expression in *Escherichia coli*. Because of the simpler annotated domain structure,^27^ the CAO homologue from *Prochlorothrix hollandica* (*Ph*CAO) was investigated first (Figures 1c and S3). Here, it was determined that *Ph*CAO could be overexpressed in *E. coli*, isolated, and reconstituted to contain three iron ions per monomer. As expected for a Rieske oxygenase,^15,16^ isolated *Ph*CAO was shown to have a trimeric architecture and display a distinctive Fe–S cluster absorption peak at approximately 430 nm (Figures 2 and S3). By capitalizing on the lessons learned in the purification of *Ph*CAO, the three other homologues, *At*CAO, *Cr*CAO, and *Mp*CAO, were subsequently isolated (Figure S3c).

### CAO Converts Chlorophyllide *a* into Chlorophyllide *b*

**Figure 3:**
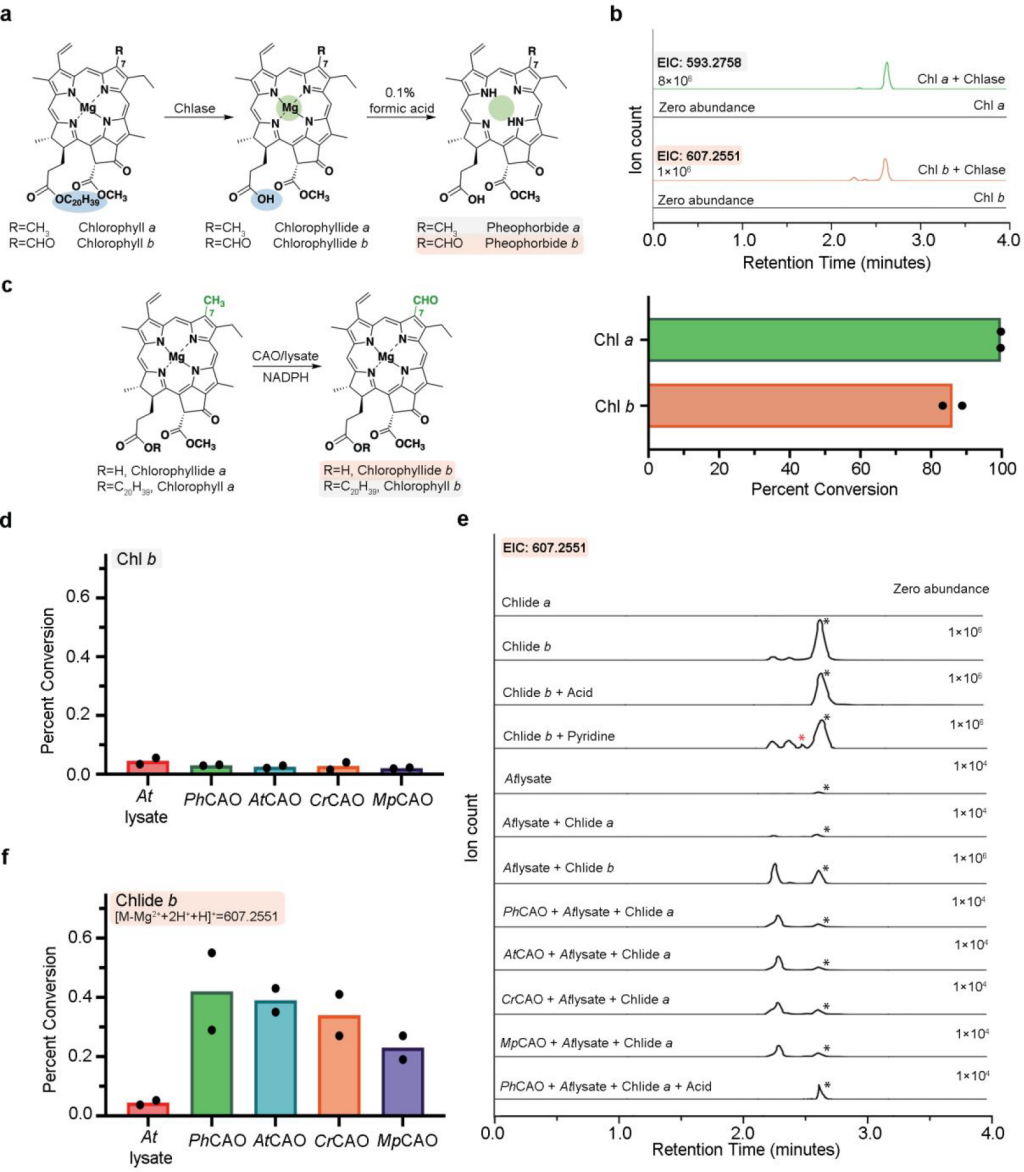
CAO homologues transform Chlide *a*, not Chl *a*, into Chlide *b* in cell lysate. (a) Combination of a Chl pigment with recombinantly expressed and purified *T. aestivum* chlorophyllase (Chlase) permits formation of the desired Chlide *a* and Chlide *b* pigments. (b) Activity of chlorophyllase on Chl *a* and Chl *b*. Extracted ion chromatograms of the chlorophyllase activity assays product with Chl *a* (top trace) and Chl *b* (bottom trace). The LC-MS method designed and employed for pigment separation in this work relies on an acidic running solvent and causes loss of the central Mg^2+^ ion and the addition of two protons to the pigments under study. Therefore, the *m*/*z* = 593.2758 and *m*/*z* = 607.2551 represents the [M + H]^+^ of Chlide *a* and Chlide *b* minus Mg^2+^ plus 2H^+^, respectively (top panel). Chlorophyllase converts nearly 100% and 85% of Chl *a* and Chl *b* into their Chlide counterparts, respectively (bottom panel). (c) Proposed reaction scheme catalyzed by CAO in cell lysate. (d) None of the CAO homologues can transform Chl *a* into Chl *b* in *A. thaliana* cell lysate. (e) The extracted ion chromatograms for the CAO homologue reaction products when combined with a Chlide *a* substrate and *A. thaliana* cell lysate reveal that all four CAO homologues can convert Chlide *a* into Chlide *b*. Of note, the cell lysate, pyridine, and acid can each shift the diastereomer equilibrium of the Chlide *b* standard (traces 3–4 and 7). The black asterisk indicates the major peak of the standard, which is also observed in the assays and the red asterisk corresponds to the diastereomer peak observed in the enzymatic assays. (f) *Ph*CAO shows the highest percent conversion among all the four homologues in the presence of *A. thaliana* cell lysate and a Chlide substrate. All data shown in the bar graphs was performed in duplicate and data are presented as mean values.

Once purified, the ability of CAO to convert both Chl *a* and Chlide *a* into Chl *b* and Chlide *b*, respectively, was investigated (Figure 3). As these experiments require both substrate and product standards, Chl *a* and Chl *b* were purchased. To obtain Chlide *a* and Chlide *b*, the serine protease chlorophyllase from *Triticum aestivum*, which catalyzes the first step of Chl catabolism, the conversion of Chl into Chlide, was recombinantly expressed, purified, and assayed for activity (Figures S3c and S4). Using LC-MS experiments, it was revealed that an approximate 1 h incubation of chlorophyllase with Chl *a* or Chl *b* resulted in near complete conversion into the respective Chlide *a* or Chlide *b* pigments (Figures 3b, S5, and S6). To test the ability of CAO to convert these potential substrates into their respective product molecules, an electron donor for the reaction also needed to be identified. As the partner reductase protein for CAO is unknown, chemical reductants, including ascorbate, sodium dithionite, titanium(III) chloride (TiCl_3_), and dithiothreitol (DTT), were individually tested in assays that contained either Chl *a* or the enzymatically produced Chlide *a*, and *Ph*CAO. Here, it was unexpectedly determined that despite the ability of sodium dithionite to reduce the Rieske cluster, that neither combination of it, nor the other tested reductants with *Ph*CAO and the possible substrates resulted in production of Chl *b* or Chlide *b* (S7a and b). Similarly, the ability of CAO to form product, was probed using the so-called peroxide shunt reaction.^31^ This reaction uses hydrogen peroxide (H_2_O_2_) and directly forms an activated oxygen species in the Rieske oxygenase active site.^31^ However, even under these conditions, no production of Chlide *b* or Chl *b* was detected (Figure S7c and d). As it has previously been shown that thiol-containing molecules can add to the C3-vinyl group of Chl, it was hypothesized that these chemicals were either unable to support the reaction or were reacting with the substrate.^32^ Indeed, the Chl absorption peaks in the presence of ascorbate, sodium dithionite, and TiCl_3_, showed notable differences, suggesting these small molecules may affect the properties and competence of the tested substrates (Figure S8).

These experiments suggested that a protein-based, rather than a small molecule reductant may be important to the experimental setup. To investigate this hypothesis, assays were performed by combining Chl *a*, *Ph*CAO, and a small amount of *A. thaliana* cell lysate (Figure 3c). This combination did not produce any Chl *b* product above background levels (Figure 3d). Replacement of *Ph*CAO with *At*CAO, *Cr*CAO, or *Mp*CAO, in an equivalent assay likewise showed no production of Chl *b* (Figure 3d). However, when the same reaction was performed with added chlorophyllase, two new peaks were formed that matched the exact mass of the expected Chlide *b* product (Figures 3e and S9a–c). The retention time of one of these peaks matched the major peak of the Chlide *b* standard (Figure 3e, black asterisk). The second peak was hypothesized to represent a diastereomer of Chlide *b*, which contains three chiral centers at C13^2^, C17, and C18 (Figures 1a and S1, red asterisk). To test this hypothesis, samples of the Chlide *b* standard were treated with either or 15-percent pyridine or 10 mM HCl (Figure 3e). These chemicals were chosen as it has previously been demonstrated that pyridine can be used to change the stereochemistry at the C-13^2^ position of Chl pigments via removal of the acidic proton and enolization (Figure S10).^33−35^ In both cases, the LC-MS peak distribution of the standard changed (Figure 3e). Similarly, incubation of the Chlide *b* standard in *A. thaliana* cell lysate showed interconversion of peaks, suggesting that there may be an enzyme in the lysate that can similarly interchange the stereochemistry of Chlide *b* using acid–base chemistry (Figure 3e). The assignment of these peaks as Chlide *b* diastereomers is further supported by MS/MS data, which yielded identical fragmentation patterns between the enzymatically produced Chlide *b* and the pyridine-treated sample (Figures S11 and S12). Substitution of *Ph*CAO in the assays that contained Chl *a*, chlorophyllase, and *A. thaliana* cell lysate with *At*CAO, *Cr*CAO, or *Mp*CAO resulted in similar formation of product diastereomers (Figures 3e,f and S9a–c).

**Figure 4:**
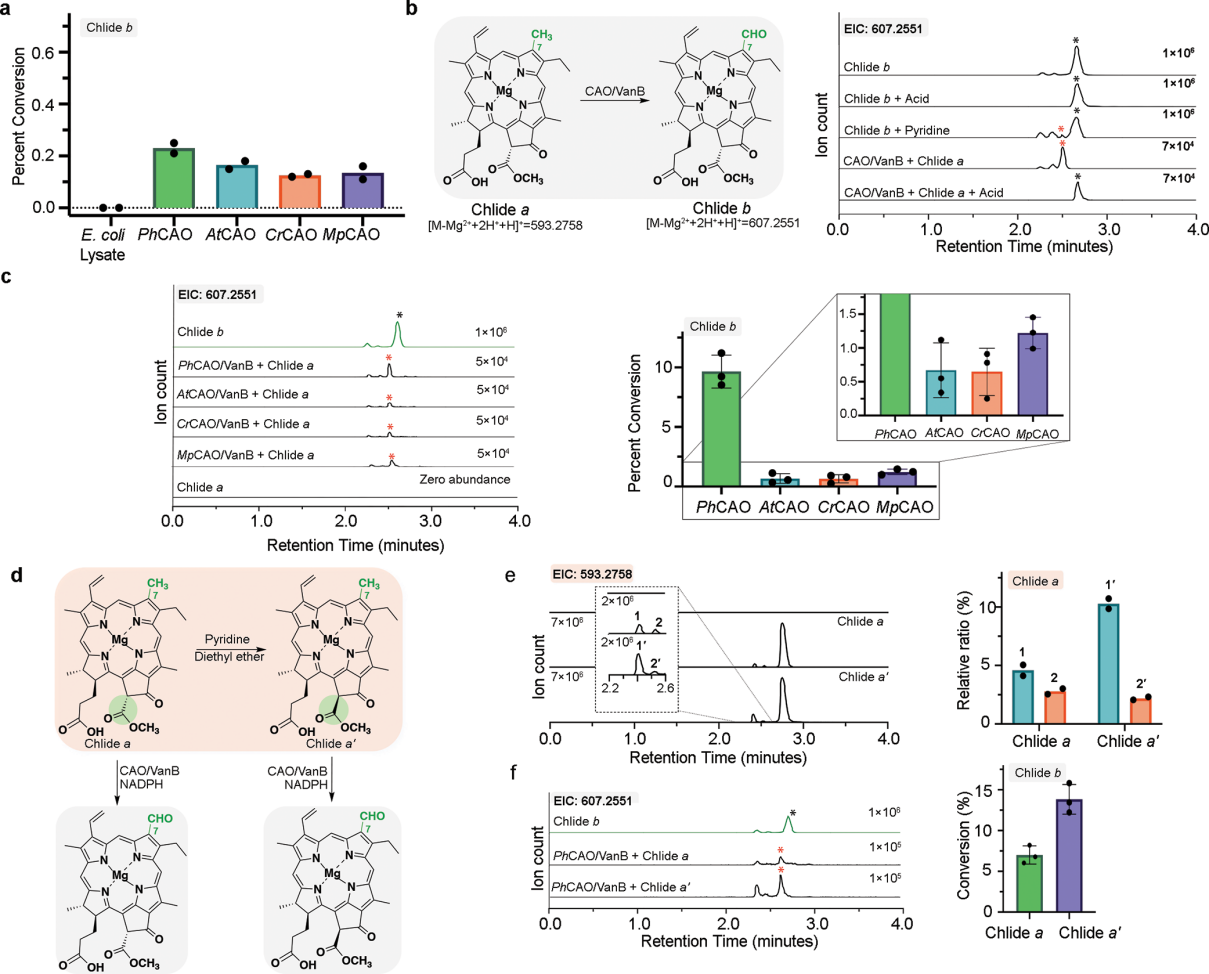
CAO homologues transform chlorophyllide *a* into chlorophyllide *b* in the presence of the non-native reductase VanB. (a) All four CAO homologues show the ability to convert Chlide *a* into Chlide *b* in *E. coli* cell lysate, suggesting that a non-native reductase may work with CAO to facilitate the reaction. *Ph*CAO shows the highest percent conversion among all four homologues. (b) Reaction scheme of the CAO/VanB catalyzed conversion of Chlide *a* into Chlide *b* (left panel). Treatment of the Chlide *b* standard with acid or pyridine shows new diastereomer peaks. Similarly, the reaction product from the CAO homologue reactions can be converted into the main diastereomer of the Chlide *b* standard under acidic conditions (right panel). (c) The extracted ion chromatograms for the product formed when the CAO homologues are combined with Chlide *a*, VanB, and NADPH. This data shows a diastereomer of Chlide *b* is formed in these reactions. Again, *Ph*CAO shows the highest percent conversion among all four homologues. (d) Reaction scheme for converting Chlide *a* into Chlide *a*′ with pyridine. (e) An extracted ion chromatogram of the Chlide *a* peak suggests the Chlide *a*′ sample shows a different diastereomer distribution than Chlide *a*. (f) *Ph*CAO shows a higher percent conversion on Chlide *a*′ than Chlide *a.* In panels c and f, reactions were performed in triplicate, and in panels a and e, they were performed in duplicate. In all bar graphs presented, data are shown as mean values.

Additional studies were performed to evaluate whether cell lysate from spinach, *Chlamydomonas reinhardtii*, or barley would support higher conversion of the other purified CAO homologues (Figure S9a–c). These experiments revealed that substitution of *A. thaliana* cell lysate with spinach cell lysate in the assays, results in a comparable amount of Chlide *b* formation in all four homologues (Figure S9b and c). The substitution of *Chlamydomonas reinhardtii* lysate, however, resulted in a much lower yield relative to *A. thaliana.* Barley lysate did not facilitate the CAO-mediated production of Chlide *b* at all (Figure S9b and c). Of note, these additional cell lysate options also failed to support the ability of CAO to convert Chl *a* into Chl *b* (Figure S9d). Finally, to evaluate whether an additional component that is found exclusively in photosynthetic organisms is required to support the activity of the different CAO homologues, the assays were also performed in *E. coli* cell lysate (Figure 4a). Unlike that previously described,^10^ without the addition of an external reducing system, these assays showed the production of a small amount of Chlide *b* and suggested the possibility that the in vitro activity of these proteins may be supported using a non-native reductase (Figure 4a).

### The Combination of CAO with a Non-native Reductase Allows for Production of Chlorophyllide *b* in Vitro

To evaluate whether a non-native reductase system, rather than cell lysate, could facilitate the CAO reaction, a flavodoxin–flavodoxin reductase system from *E. coli* and several different known Rieske reductase systems were purified (Figure S3c). These systems include the ferredoxin-NAD^+^ reductases, VanB and TsaB, and the two-component [2Fe-2S] cluster containing ferredoxin (DdmB) and ferredoxin–NAD^+^ reductase system (DdmA). These proteins, as well as a spinach ferredoxin–ferredoxin reductase system, were subsequently tested as candidate reductases for the CAO homologues (Figure S13). Here, the combination of a CAO homologue with Chl *a*, chlorophyllase, and either *E. coli* flavodoxin–flavodoxin reductase or spinach ferredoxin–ferredoxin reductase did not result in any detectable production of Chlide *b* (Figure S13). In contrast, the use of VanB, DdmB/DdmA, or TsaB and NADPH in the assays resulted in production of a compound, which has the same molecular weight as Chlide *b* but is shifted in retention time relative to the enzymatically produced Chlide *b* standard (Figure 4b, red asterisk and Figure S13). The highest amount of this compound was produced in the assays that contained VanB (Figures S13 and 4b). Although produced to a much greater extent, like that described above, the retention time of this molecule could be shifted to match the Chlide *b* standard by the addition of *A. thaliana* cell lysate or by treatment with acid (Figure 3e and 4b). Similarly, formation of a matching peak could be made in the Chlide *b* standard via the addition of pyridine (Figure 4b). Last, as it appeared that VanB could support the CAO-catalyzed production of Chlide *b*, it was investigated whether it was providing the needed electrons for the reaction. Here, it was determined that, as observed with the small molecule chemical reductants, VanB and NADPH also facilitate reduction of the Rieske [2Fe-2S] cluster in *Ph*CAO (Figure S14). However, as VanB works as an electron mediator and delivers electrons directly from NADPH to the Rieske cluster, we suggest that its ability to prohibit these electrons from reacting with the Chlide *a* substrate, the protein, or an activated oxygen intermediate, is integral to its success participating in this reaction (Figures S8 and S13). The *At*CAO, *Cr*CAO, and *Mp*CAO homologues were then tested in equivalent assays that contained VanB, NADPH, and Chlide *a* (produced in a chlorophyllase reaction). Here, it was demonstrated that each homologue yielded the same diastereomer of Chlide *b*, albeit to a lower extent than *Ph*CAO (Figure 4c).

### CAO Shows a Preference for a Chlide *a*′ Diastereomer

The intriguing observation that CAO produces a different major diastereomer of Chlide *b* than that observed in the Chlide *b* standard, prompted an investigation into the preferred Chlide *a* substrate diastereomer. As described above, previous work has demonstrated that the addition of pyridine to a Chl pigment can change the absolute configuration at the C-13^2^ position.^33−35^ A similar phenomenon has also been shown to occur following addition of triethylamine to Chl.^33^ Therefore, to investigate the preferred diastereomer of the CAO-catalyzed reaction, both methods were tested for their ability to produce chlorophyll *a*′ (Chl *a*′) from Chl *a*. Chl *a*′ has an (*S)*-13^2^-carbomethoxy group rather than the (*R)*-13^2^-carbomethoxy group found in Chl *a*. In these experiments, only subtle changes were noticed in the LC-MS analysis after addition of triethylamine (Figure S15). In contrast, the product of the pyridine-treated reaction, which is a mixture of Chl *a*′ and Chl *a*,^35^ shows a near 2-fold increase in the size of one diastereomer peak following the chlorophyllase catalyzed hydrolysis reaction (Figure 4d and e). Despite these differences, the chlorophyllase-cleaved samples of Chl *a*′ and Chl *a* behave similarly in cell lysate, again suggesting the presence of an enzyme that can change the stereochemistry of Chlide molecules (Figure S16). More interestingly, however, combination of the produced Chlide *a*′ mixture with *Ph*CAO, VanB, and NADPH resulted in almost twice as much Chlide *b* product (Figure 4f).

Related experiments on extracted chlorophyllase from *Melia azedarach* and *Tetragonia ezpansa* have also revealed that chlorophyllase is stereospecific. Chlorophyllase prefers a substrate that has an (*R)*-13^2^-carbomethoxy group and shows a preference for hydrolyzing Chl *a* into Chlide *a* rather than Chl *a*′ into Chlide *a*′.^36^ Our data on chlorophyllase are consistent with this previous work: the purchased Chl *a* is a mixture of diastereomers that appear as different peaks in the LC-MS experiment. Incubation of this mixture for an extended amount of time with chlorophyllase results in complete hydrolysis but the major diastereomer of the purchased Chl *a* is consumed at a faster rate (Figure S17). Treatment of Chl *a* with pyridine and subsequent hydrolysis with chlorophyllase also leads to complete conversion into Chlide, but as described above, the product mixture has a different diastereomer distribution than the nonpyridine-treated Chlide product (Figures 4e and S18). Combined with the data that shows incubation of the Chl *b* product standard with pyridine results in the production of a peak that matches the major peak produced in the in vitro CAO reaction, these data suggest that CAO shows a clear preference for a Chlide *a*′ substrate and produces a major product that is the equivalent to the sample of pyridine treated Chlide *b* (Figure 4f). As observed for *Ph*CAO, the other homologues are also able to use Chlide *a*′ as a substrate, albeit to different extents (Figure S18).

### CAO is a Rieske Oxygenase that Catalyzes Two Monooxygenation Reactions

**Figure 5:**
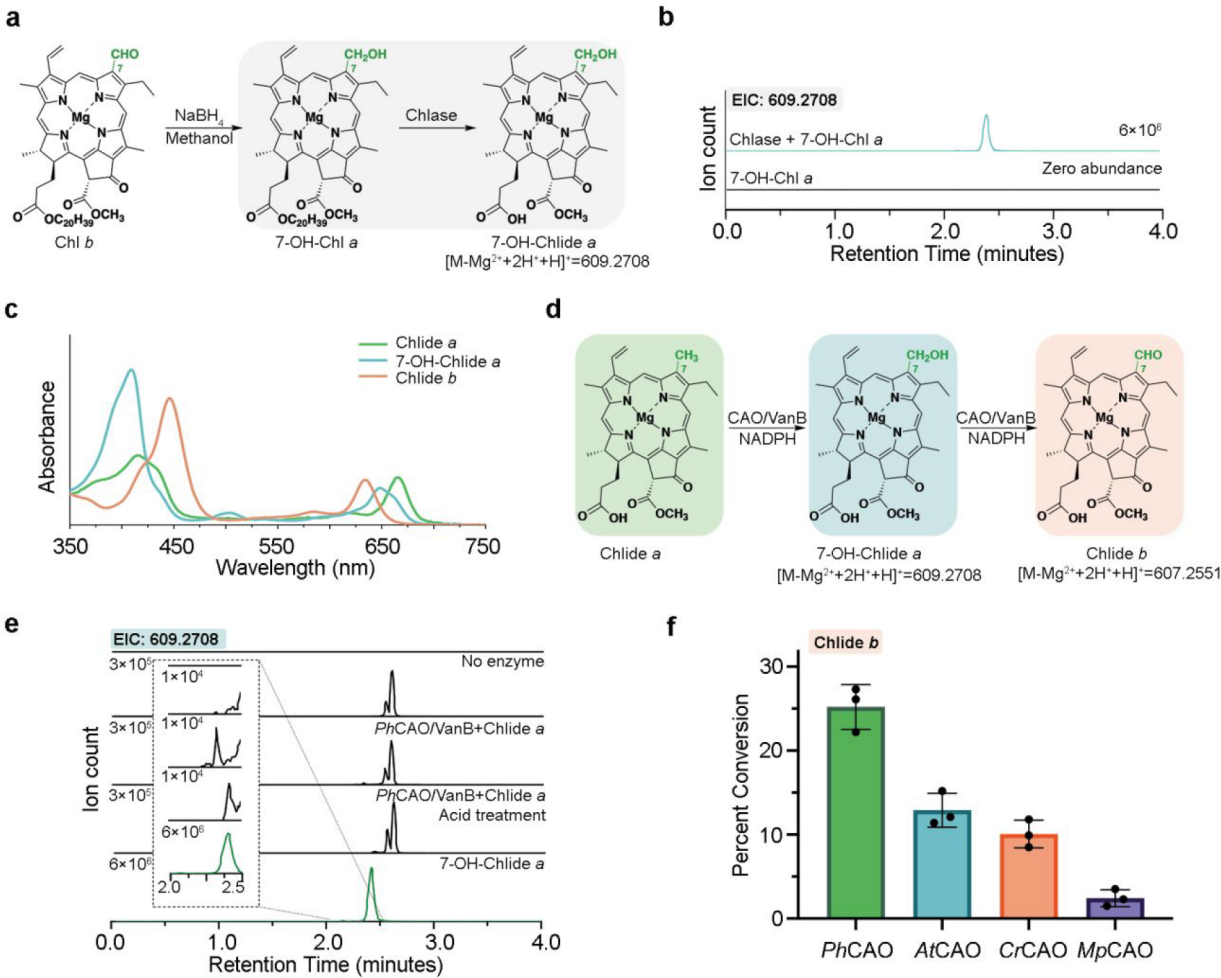
7-OH-Chlide *a* can be transformed into Chlide *b* by combination of the CAO homologues with VanB and NADPH. (a) Reaction scheme for synthetic conversion of Chl *b* into 7-OH-Chlide *a*. (b) Extracted ion chromatograms of chlorophyllase catalyzed hydrolysis of 7-OH-Chl *a*. The *m*/*z* = 609.2708 represents the [M + H]^+^ of 7-OH-Chlide *a* minus Mg^2+^ plus 2H^+^ (see Figure 3a). (c) Absorbance spectra of the Chlide *a*, Chlide *b*, and 7-OH Chlide *a* that were enzymatically and synthetically produced in this work. (d) Reaction scheme of the oxygenation reactions that are catalyzed by CAO. (e) Extracted ion chromatograms for the *Ph*CAO reaction products reveals the formation of a 7-OH-Chlide *a* intermediate. Acid treatment refers to a screening that was performed over a range of different pH values. This screening revealed that when the pH was adjusted to a value of 2, all Chlide *b* appeared as one peak. (f) All four CAO homologues show the ability to transform the intermediate (7-OH-Chlide *a*) into the final product (Chlide *b*). Again, *Ph*CAO shows the highest percent conversion among all four homologues.

The proposal in Figure 1a suggests that CAO works by catalyzing two sequential monooxygenation reactions that transform the C7-methyl group into a formyl group via a 7-hydroxymethyl intermediate. To probe whether 7-OH-Chlide *a* is a true intermediate of the CAO reaction in vitro, 7-OH-Chlide *a* was produced in two steps. First, the formyl group of Chl *b* was reduced with sodium borohydride to produce 7-OH-Chl *a*(37,38) and second, the phytol tail of 7-OH-Chl *a* was cleaved using purified *T. aestivum* chlorophyllase (Figures 5a, S19, and S20). The successful production of the 7-OH-Chlide *a* molecule was then evaluated using LC-MS and UV–vis spectroscopy (Figure 5b and c). LC-MS demonstrated production of a single peak and the Q bands of the UV–vis absorption spectrum of this pigment, as previously described,^37,38^ are blue-shifted relative to Chlide *a* (Figure 5b and c). The produced 7-OH-Chlide *a* compound was then used as a standard to look for its production in the assays that contained *Ph*CAO, VanB, NADPH, Chl *a*, and chlorophyllase (Figure 5d). This experiment showed the existence of two peaks in the CAO-lacking control reactions that showcase different retention times and have the expected mass of the 7-OH-Chlide *a* intermediate (Figure 5e). However, in the LC-MS trace from the reaction that contained CAO, one peak at relatively low intensity was present that did not appear in the controls (Figure 5e). This peak displayed a different retention time compared to the 7-OH-Chlide *a* standard, but like what was observed in the CAO-Chlide *a* reaction, the retention time of this peak shifted when treated with acid (Figure 5e). Again, this peak was hypothesized to be a diastereomer of 7-OH-Chlide *a* and MS/MS analysis revealed that this peak had a similar fragmentation pattern to the produced 7-OH-Chlide *a* standard (Figure S21).

To further investigate the hydroxylation proposal, 7-OH-Chlide *a*, the proposed intermediate, was added into an assay that contained CAO, VanB, and NADPH as a substrate. In this experiment, it was observed using LC-MS, that all four CAO homologues could produce Chlide *b*. In these reactions, the product displayed the exact same retention time as the main peak of the Chlide *b* standard, which is presumably due to it being synthesized from the same Chl *b* precursor (Figure S22). Again, when provided with the 7-OH-Chlide *a* substrate, *Ph*CAO showed the highest percent conversion among all four CAO homologues (Figures 5f and S22). Importantly, it was determined that Chlide *b* production was observed only in the case where both CAO and the reductase were included in the reaction, suggesting that this second reaction is indeed completed using Rieske oxygenase chemistry (Figure S22b). Therefore, the steady-state kinetic behavior was tested for *Ph*CAO using a 7-OH Chlide *a* substrate. Here, it was determined that the Michaelis constant is 7.8 μM and the *k*_cat_= 0.12 min^–1^ (Figure S23). It is also worth noting that in these reactions the dihydroxylated intermediate was not observed, presumably due to the spontaneous loss of water and immediate collapse into a Chlide *b* product. Collectively, these results show that CAO is a Rieske oxygenase that performs two oxygenation reactions in vitro when combined with an annotated ferredoxin-NAD^+^ Rieske reductase, VanB.

### Factors That Dictate the Substrate Scope of CAO

**Figure 6:**
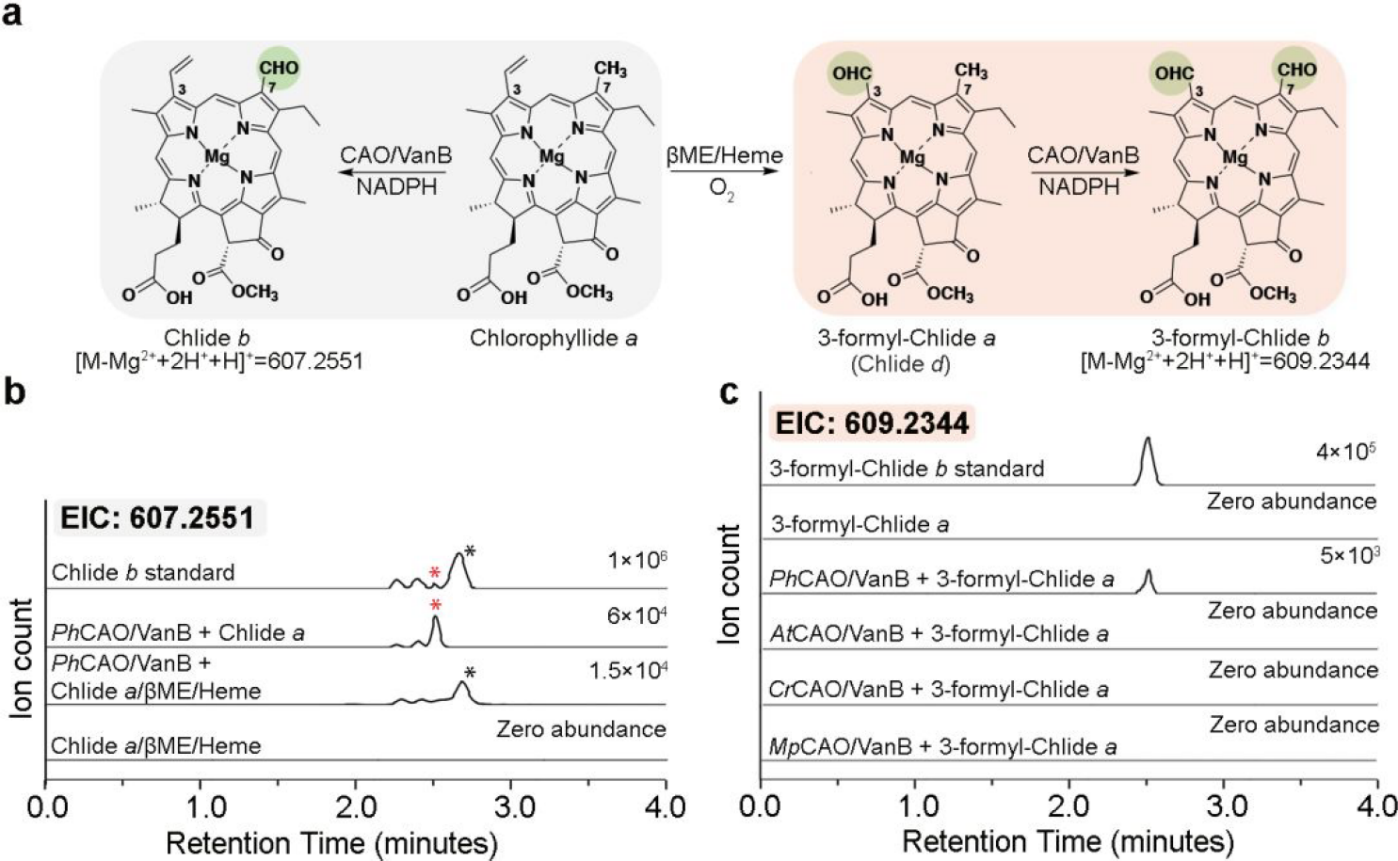
3-formyl-Chlide *a* (Chlide *d*) can be transformed into 3-formyl-Chlide *b* by combination of *Ph*CAO with VanB and NADPH. (a) Reaction scheme to synthesize 3-formyl-Chlide *a* and the proposed route to C7-oxygenation by CAO. (b) The extracted ion chromatograms for the product formed when Chlide *a* or Chlide *a* with β-mercaptoethanol and heme was provided to *Ph*CAO as a substrate. (c) The extracted ion chromatograms show that *Ph*CAO can transform 3-formyl-Chlide *a* (Chlide *d*) into 3-formyl-Chlide *b*.

As described above, none of the CAO homologues purified in this work are able to oxidize the C7-methyl group of Chl *a* into the formyl group of Chl *b* either in the presence or absence of cell lysate (Figures 3d and S24a). Rather, each of the tested homologues shows a preference for a Chlide *a* or Chlide *a*′ substrate. To further explore the scope of substrates accepted by CAO, several Chlide *a* analogs, including pheophorbide *a*, bacteriochlorophyll (Bchl) *a*, bacteriochlorophyllide *a* (Bchlide *a*), and Chlide *d* were tested for their propensity to be oxidized by *Ph*CAO. Here, it was determined that pheophorbide *a*, which lacks the central Mg^2+^ ion of Chlide *a*, is not a substrate of *Ph*CAO (Figure S24b). Similarly, Bchl *a*, which resembles Chl *a* but instead contains a bacteriochlorin scaffold and a C3-acetyl group, rather than a chlorin scaffold and a C3-vinyl group, is also not a substrate of *Ph*CAO (Figure S25b). Bchlide *a*, produced via combination of commercially purchased Bchl *a* with chlorophyllase, was also not formylated by *Ph*CAO (Figure S25a and c). Last, a report which showed that a doubly formylated Chl species, 7-formyl Chl *d*, could be produced by transformation of *Ph*CAO into the Chl *d* producer, *Acarychloris marina*, was investigated.^39^ Specifically, as this in vivo experiment did not provide details about the order of the formylation reactions, it was explored whether CAO could formylate Chlide *d* in vitro. To produce Chlide *d*, chlorophyllase was first used to produce Chlide *a*. Chlide *a* was then transformed into Chlide *d* using a recently described protocol for producing Chl *d* from Chl *a* in vitro^32^ (Figure 6a and S26). When this reaction mixture was given to *Ph*CAO as a substrate, formation of both Chlide *b* and a second product was observed (Figure 6b and c). This second product is present at relatively low yields but has a mass that is consistent with it being 3-formyl-Chlide *b* (Figure 6c). To investigate the nature of this molecule, a standard of 3-formyl-Chlide *b* was produced similarly to Chlide *d*, except that Chl *b* was used as a starting material (Figure 6c). This standard showed the same retention time as the product made by *Ph*CAO and had a similar MS/MS fragmentation pattern, indicating that *Ph*CAO can accept a pigment that is formylated at the C3 position to produce a non-natural 3-formyl-Chlide *b* pigment (Figure S27).

## Discussion

Despite the identification of CAO over 20 years ago,^10^ the mechanism of the required methyl-to-formyl group transformation in chlorophyll *b* biosynthesis has never been studied in vitro with purified protein. Traditionally, these studies have been hindered by the lack of published protocols for the recombinant expression and purification of a CAO homologue, the reported insolubility and reactive nature of the proposed substrates, and the lack of an annotated reductase for the reaction. To overcome these obstacles, in this work, a homogeneous and reconstituted “simple” CAO that lacks the accessory domains found in *A. thaliana* and *C. reinhardtii* homologues and carries both the Rieske cluster and nonheme iron site on a single polypeptide chain was recombinantly expressed and purified (Figures 2 and S2–S3). The successful methods for isolating *Ph*CAO were then extended to isolate other CAO homologues (Figure S3). Each of these purified CAO homologues were then shown to convert Chlide *a* into Chlide *b* rather than Chl *a* into Chl *b* in cell lysate and in the presence of the non-native Rieske reductase VanB (Figures 3 and 4). Thus, this work suggests that despite previous proposals, Chl *a* is not formylated by CAO even in the presence of other photosynthetic proteins that could function as carriers of Chl in the native organism.^4,40,41^ Likewise, the ability of chlorophyllase to cleave Chl into Chlide in our assays negates previous proposals^40,41^ that the insolubility of Chl prohibits its ability to serve as an enzyme substrate in vitro.

This work also showed that all four CAO homologues, which come from different kingdoms of life, and contain different annotated architectures,^27^ each function similarly and can use a combination of NADPH and the same non-native reductase as a source of electrons. The ability of *Mp*CAO to accept electrons from VanB and produce Chlide *b* is particularly surprising as it has been previously suggested to exist as a heterodimer,^27^ rather than as a prototypical Rieske oxygenase trimer. However, on the basis of previous work that shows Rieske reductase proteins, like VanB, bind to the Rieske oxygenase at the interfaces between adjacent protomers,^25,42^ it is likely that *Mp*CAO forms an architecture, such as a trimer of dimers, that is more similar to the prototypical Rieske oxygenases. Future structural work to reveal how these proteins assemble and interact with a reductase are paramount to our understanding of this enzyme class. Nevertheless, small changes in the way that the*Mp*CAO subunits arrange and the presence of extra N-terminal regulatory domains in the *A. thaliana* and *C. reinhardtii* homologues, which have been suggested to sense Chl *b* accumulation and/or destabilize CAO,^43^ may be partially responsible for their lower observed in vitro activity relative to *Ph*CAO (Figures 1c, 4c, and S2). Most notably, in this work, it was determined that these CAO-catalyzed reactions form a different diastereomer of Chlide *b* than what is found in the Chlide *b* standard (Figures 3 and 4). As the product formed can be converted into the standard by the addition of pyridine, we propose that the CAO product is natively Chlide *b*′ (Figures 3 and 4). Curiously, it is also known that the last step of Chl synthesis requires the Chl synthetase-catalyzed attachment of the hydrophobic tail (Figure S4).^44^ This protein, like chlorophyllase is known to show a preference for both Chlide *a* and Chlide *b* substrates, rather than Chlide *a*′ and Chlide *b*′.^44^ The observation that cell lysate shows the remarkable ability to interchange diastereomer peaks may suggest that there are epimerase enzymes present that are involved in regulating which Chl and Chlide diastereomers are present in the cell (Figure 3e).

This work also used MS experiments to reveal that 7-OH-Chlide *a*, is a true intermediate of the reaction and shows that CAO is a Rieske oxygenase that can facilitate two oxygenation reactions in lieu of an additional Rieske protein, dehydrogenase, or cofactor (Figure 5). Using 7-OH-Chlide *a* as a substrate, it was determined that *Ph*CAO displays a *K*_M_ of 7.8 ± 0.9 μM and a *k*_cat_ of 0.12 min^–1^ (Figure S23). This *K*_M_ is consistent with that determined for other Chl biosynthetic enzymes, including AcsF (7.0 μM)^45^ and light-dependent protochlorophyllide oxidoreductase (8.6 μM).^46^ The *k*_cat_ for the second oxygenation reaction catalyzed by CAO, on the other hand, is lower than that recently described for AcsF (0.9 min^–1^)^45^ and may reflect the complexity of the reaction, which requires chlorophyllase to cleave the phytol tail of the substrate and a nonphysiological reductase to deliver electrons. In addition, it may correlate with providing the nonpreferred substrate diastereomer in the assay, or may suggest that as previously described for other Chl biosynthetic enzymes,^47^ the in vivo rate is amplified by protein–protein interactions with other pathway enzymes.

Last but not least, along with Chl *a*, several Chlide *a* analogs were used to test the substrate requirements of the CAO reaction. Chl *a*, pheophorbide *a*, Bchl *a*, and Bchlide *a*, were each shown to not be formylated by CAO (Figures S24 and S25). These results suggest that the presence of a phytol side chain at the C17 position, the lack of a central metal ion, and the bacteriochlorin scaffold each effect the reactivity of CAO. We propose that the lack of a phytol chain and presence of a metal ion are important dictators of substrate binding in CAO. In addition, these data suggest that the electronics of the substrate may be significant for the CAO reaction. In the case of both Bchl *a* and Bchlide *a*, the B ring is oxidized by two electrons relative to Chl *a* and Chlide *a* (Figure S25). This structural feature means that for CAO to accept these molecules as a substrate, that a C7-substrate radical must be produced adjacent to an sp^3^-, rather than an sp^2^-hyridized carbon atom. We hypothesize that due to this difference, CAO is unable to abstract the needed hydrogen atom to initiate the formylation reaction. Protochlorophyllide (Pchlide) *a* also resembles Chlide *a*, but it has a 22-electron-containing scaffold. Conflicting previous experiments performed in cell lysate suggested that CAO either could^48^ or could not^10^ convert a small amount of this molecule into Pchlide *b*. Intriguingly, however, a second Rieske oxygenase, protochlorophyllide *a* oxygenase (PTC52), is instead credited for this transformation.^48^ Most interestingly, it was determined here that 3-formyl-Chlorophyllide *a*, or Chlorophyllide *d*, can be accepted by *Ph*CAO and a second formyl group can be installed at the C7 position to produce 3-formyl-Chlide *b* (Figure 6). Collectively, these results highlight the remarkable flexibility of the active site for binding pigment substrates, that lack a phytol tail and contain a central metal ion and a chlorin scaffold.

Therefore, this work adds to our knowledge of chlorophyll biosynthesis, extends the known reactivity of the Rieske oxygenase class, and provides a framework for developing the CAO-VanB system as a tool to produce non-native Chl pigments (Figure 1b). The procedures established in this work for expressing, purifying, and reconstituting four CAO homologues can now be used to facilitate future structural and biochemical studies on CAO. Likewise, the procedures developed for production of the desired Chlide molecules and bottom-up approach for measuring activity CAO activity have revealed intriguing details regarding the stereoselectivity and substrate scope of the reaction. Future work will be aimed at pinpointing key amino acids involved in the transformation of Chlide *a* into Chlide *b*, optimizing the partner reductase used in the reaction, identifying the component(s) in cell lysate that change the stereochemistry of Chlide, determining whether the CAO reactions are catalyzed in a processive manner or via release of the 7-OH-Chlide *a* intermediate after one catalytic cycle, and increasing the yields of native and non-native pigments.

## Materials and Methods

Methods and any associated references are available in the Supporting Information.

## Supporting Information Available

Structures of chlorophyll (Chl) and chlorophyllide (Chlide) pigments, sequence alignment of CAO homologs used in this work, purification of CAO homologs, enzymes involved in Chl *b* metabolism, a standard curve was used to convert Q-TOF LC-MS peak area into the amounts of pigments that were produced and consumed in the chlorophyllase-catalyzed reactions, chlorophyllase can be used as a tool to produce the needed Chlide *a* and Childe *b* standards, several chemical reductants and the peroxide (H_2_O_2_) shunt reaction were tested in the CAO reactions, addition of sodium dithionite, ascorbate, and TiCl_3_ change the distinctive absorption peaks of chlorophyll *a*, cell lysate from four different photosynthetic organisms was tested for its ability to support Chlide *b* production, stereochemistry at the C-13^2^ position of Chl pigments can be changed using acid-base chemistry, analysis of the enzymatically produced Chlide *a* standard, analysis of the enzymatically produced Chlide *b* standard and the product of the CAO reaction, five different non-native reductase systems were tested for their ability to support Chlide *b* production, VanB can mediate the reduction of the Rieske cluster in *Ph*CAO, diastereomer distribution of Chlide *a* and Chlide *b* can be shifted by triethylamine (TEA) or pyridine, diastereomer equilibrium of the Chlide *a* and Chlide *a*′ can be shifted by *A. thaliana* cell lysate, a certain diastereomer of Chl *a* is preferred by chlorophyllase, the Chlide *a*′ diasteromer is preferred by all tested homologs of CAO, standard curve for Q-TOF LC-MS was used to determine the amount of intermediate (7-OH-Chlorophyllide *a*) in the reactions, chlorophyllase can be used as a tool to produce a 7-OH-Chlide*a* standard, analysis of the chemically produced 7-OH-Chlide a standard and the intermediate produced in the CAO reaction, extracted ion chromatograms for the CAO homolog reaction products when combined with VanB, NADPH, and a 7-OH-Chlide *a* substrate, steady state kinetic behavior of*Ph*CAO with a 7 OH-Chlide *a* substrate, chlorophyll*a* and pheophorbide *a* are not substrates of the CAO homologs, activity of the CAO homologs was tested on bacteriochlorophyll *a* and bacteriochlorophyllide *a* substrates, analysis for the chemically produced Chlide *d* (3-formyl-Chlide *a*), analysis for the 3-formyl-Chlide *b* produced in the CAO reaction, DNA and protein sequences, protein production and purification, preparation of substrates and product standards, enzymatic reactions, and supplementary references (PDF)
