# Supplementary material for: Rieske Oxygenase Catalyzed C–H Bond Functionalization Reactions in Chlorophyll b Biosynthesis
Source: ACS Cent Sci. 2022 Jul 27;8(10):1393–403. doi: 10.1021/acscentsci.2c00058 (PMC9615114; doi:10.1021/acscentsci.2c00058)
Supplement: Supplementary file 1 — oc2c00058_si_001.pdf [file oc2c00058_si_001.pdf]

Supporting Information For:

Rieske Oxygenase Catalyzed C–H Bond Functionalization Reactions in Chlorophyll *b* Biosynthesis

Jianxin Liu, Madison Knapp, Minshik Jo, Zerick Dill, and Jennifer Bridwell-Rabb*

Department of Chemistry, University of Michigan, Ann Arbor, Michigan, 48109

*To whom correspondence should be addressed: jebridwe@umich.edu

Table of Contents:

Table of Contents: S1-S2

I. Supplementary Figures S3-S27

Supplementary Figure 1. Structures of Chlorophyll (Chl) *and* Chlorophyllide (Chlide) pigments *a* and *b*.

Supplementary Figure 2. Sequence alignment of CAO homologs used in this work.

Supplementary Figure 3. Purification of CAO homologs.

Supplementary Figure 4. Enzymes involved in Chl *b* metabolism.

Supplementary Figure 5. A standard curve was used to convert Q-TOF LC-MS peak area into the amounts of pigments that were produced and consumed in the chlorophyllase-catalyzed reactions.

Supplementary Figure 6. Chlorophyllase can be used as a tool to produce the needed Chlide *a* and Childe *b* standards.

Supplementary Figure 7. Several chemical reductants and the peroxide (H_2O_2) shunt reaction were tested in the CAO reactions.

Supplementary Figure 8. Supplementary Figure 6. The addition of sodium dithionite, ascorbate, and $TiCl_3$ change the distinctive absorption peaks of Chlorophyll *a***.**

Supplementary Figure 9. Cell lysate from four different photosynthetic organisms was tested for its ability to support Chlide *b* production.

Supplementary Figure 10. The stereochemistry at the C-13^2 position of Chl pigments can be changed using acid-base chemistry.

Supplementary Figure 11. Analysis of the enzymatically produced Chlide *a* standard.

Supplementary Figure 12. Analysis of the enzymatically produced Chlide *b* standard and the product of the CAO reaction.

Supplementary Figure 13. Five different non-native reductase systems were tested for their ability to support Chlide *b* production.

Supplementary Figure 14. VanB can mediate the reduction of the Rieske cluster in *Ph*CAO.

Supplementary Figure 15. The diastereomer distribution of Chlide *a* and Chlide *b* can be shifted by triethylamine (TEA) or pyridine.

Supplementary Figure 16. The diastereomer equilibrium of the Chlide *a* and Chlide *a′* can be shifted by *A. thaliana* cell lysate.

Supplementary Figure 17. A certain diastereomer of Chl *a* is preferred by chlorophyllase.
Supplementary Figure 18. The Chlide *a′* diastereomer is preferred by all tested homologs of CAO.
Supplementary Figure 19. A standard curve for Q-TOF LC-MS was used to determine the amount of intermediate (7-OH-Chlorophyllide *a*) in the reactions.
Supplementary Figure 20. Chlorophyllase can be used as a tool to produce a 7-OH-Chlide *a* standard.
Supplementary Figure 21. Analysis of the chemically produced 7-OH-Chlide *a* standard and the intermediate produced in the CAO reaction.
Supplementary Figure 22. Extracted ion chromatograms for the CAO homolog reaction products when combined with VanB, NADPH, and a 7-OH-Chlide *a* substrate.
Supplementary Figure 23. Steady state kinetic behavior of *Ph*CAO with a 7 OH-Chlide *a* substrate.
Supplementary Figure 24. Chlorophyll *a* and Pheophorbide *a* are not substrates of the CAO homologs.
Supplementary Figure 25. The activity of the CAO homologs was tested on Bacteriochlorophyll *a* and Bacteriochlorophyllide *a* substrates.
Supplementary Figure 26. Analysis for the chemically produced Chlide *d* (3-formyl-Chlide *a).*
Supplementary Figure 27. Analysis for the 3-formyl-Chlide *b* produced in the CAO reaction.

II. Supplementary Methods S28-S40

IIa. DNA and protein sequences

IIb. Protein production and purification

IIc. Preparation of Substrates and Product Standards

IId. Enzymatic Reactions

III. Supplementary References S41

I. Supplementary Figures

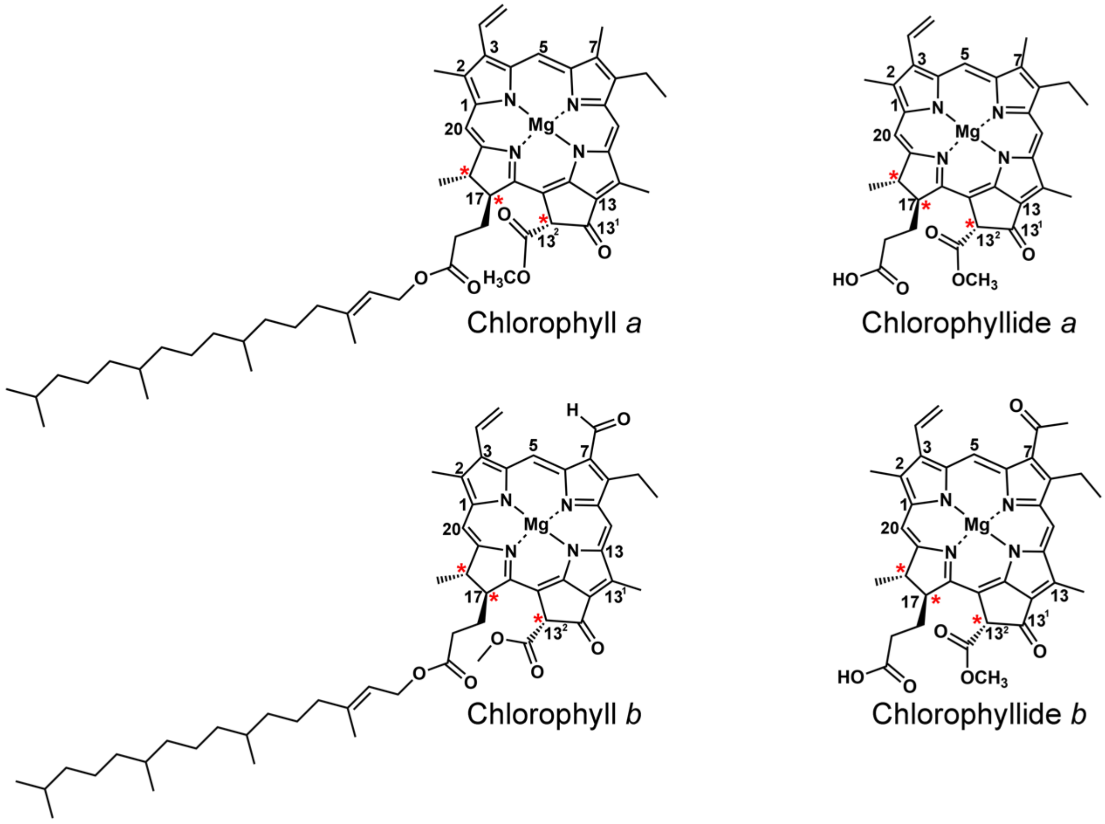

Supplementary Figure 1. Structures of Chlorophyll (Chl) *and* Chlorophyllide (Chlide) pigments *a* and *b*. Each pigment has the scaffold carbon atoms numbered and the chiral centers are indicated by a red asterisk. Chlorophyll a differs from Chlorophyllide by the presence of a phytol tail ($C_{20}H_{39}$).

```
CrCAO    -----------MLPASLQRKAAAVGGRGPTNQSRVAVRVSAQPKEAPPASTPIVEDPESK    49
PhCAO    ------------------------------------------------------------    0
AtCAO    MNAAVFSPSALSLPISFSKTRSSFLSRK-----------------------KGVKGEFRV    37

CrCAO    FRRYGKHFGGIHKLSMDWLDSVPRVRVRTKDSRQLDDMLEL--AVLNERLAGRLEPWQAR    107
PhCAO    ------------------------------------------------------------    0
AtCAO    FAVFGDESGLV-EKKSQWRPLFDVEDPRSKAPPYKGKFLDVNQAIEVARFDIQYLDWRAR    96

CrCAO    QKLEYLRKRRKNWERIFEYVTRQDAAATLAMIEEANRKVEESLSEEAREKTAVGDLRDQL    167
PhCAO    ------------------------------------------------------------    0
AtCAO    QDLL--------------------------TIMILHDKVVDVLNPLAREYKSIGTVKKEL    130

CrCAO    ESLRAQVAQAQERLAMTQSRVEQNLQRVNELKAEATTLERMRKASDLDIKERERIAISTV    227
PhCAO    ------------------------------------------------------------    0
AtCAO    AGLQEELSKAHQQVHISEARVSTALDKLAHMEEL----------------VNDRLLPGRV    174

CrCAO    AAKGPASSSSSAAAVSAPATSATLTVERPAATTVTQEVPSTSYGTPVDRAPRRSKAAIRR    287
PhCAO    ---------------------------------------------------------MNN    3
AtCAO    VT----------------------ELDKPSSSTTAS-------AVELDRE---KTNTGAK    202

CrCAO    SRG-LESSMEIEEGLRNFWYPAEFSARLPKDTLVPFELFGEPWVMFRDEKGQPSCIRDEC    346
PhCAO    SLNVSAT-LDLANGLRNFWYPVEFSKNLGMADPLGFELFDQCWVLFRDDQGTAACILDEC    62
AtCAO    SLNVSGPVPPYSPHLKNFWYPVAFTADLKHDTMVPIECFEQPWVIFRGEDGKPGCVRNTC    262

CrCAO    AHRGCPLSLGKVVEGQVMCPYHGWEFNGDGACTKMPSTPFCRNVGVAALPCAEKDGFIWV    406
PhCAO    AHRACPLSLGKVIQGRIQCPYHGWEYDRQGECVHMPSCQAIS-NPILTLPVMEQGGMIWV    121
AtCAO    AHRACPLDLGTVNEGRIQCPYHGWEYSTDGECKKMPSTKLLK-VKIKSLPCLEQEGMIWI    321

CrCAO    WPGDGLPAETLPDFA-QPPEGFLIHAEIMVDVPVEHGLLIENLLDLAHAPFTHTSTFARG    465
PhCAO    WPGTDEPGAL-PSLAPTLPDNFTLQAELVMDLEVEHGLMLENLLDLAHAPFTHTGTFAKG    180
AtCAO    WPGDEPPAPILPSL--QPPSGFLIHAELVMDLPVEHGLLLDNLLDLAHAPFTHTSTFAKG    379

CrCAO    WPVPDFVKFHA--NKALSGFWDPYPIDMAFQPPCMTLSTIGLAQPGKIMRGVTASQCKNH    523
PhCAO    WPVPPFVRFANAATTPWTGHWDPYPIHMTFEPPCFVISTIGLR----------GKDCGRH    230
AtCAO    WSVPSLVKFLTP-TSGLQGYWDPYPIDMEFKPPCIVLSTIGISKPGKL-EGKSTQQCATH    437

CrCAO    LHQLHVCMPSKKGHTRLLYRMSLDFLPWMRHVPFIDRIWKQVAAQVLGEDLVLVLGQQDR    583
PhCAO    LHQVHACLPRGQGRTRLLYRLALDFGHWLRWVPGTHCLWQHLANRVIQEDLRLVQGQQER    290
AtCAO    LHQLHVCLPSSKNKTRLLYRMSLDFAPILKNLPFMEHLWRHFAEQVLNEDLRLVLGQQER    497

CrCAO    MLRGGSNWSNPAPYDKLAVRYRRWRNGVNAEVARVRAG-----E-PPSNPVAMS-----A    632
PhCAO    LKGGANVWNQPVGYDKLGVAYRHWRNQVERHGSDWPESPADEGREPALNAIVTGSDAPIT    350
AtCAO    MLNGANIWNLPVAYDKLGVRYRLWRNAVDRGDDKLPFSG---------------------    536

CrCAO    GEMFSVDEDDMDN---    645
PhCAO    GSVVSLPPSQAPPTGH    366
AtCAO    ----------------    536
```

```
MpCAO1    ------------------------------------------------------------    0
PhCAO     ---------------------------MNNSLNVSAT---LDLANGLRNFWYPVEFSKNL    30
MpCAO2    MAPEVSSSPAPVDASRGGAEKAARRLGMGRRFADEVSLDNPSDVGAIRNYWYPIHFISKL    60

MpCAO1    -----MIPFDLFNVPWVAFRDQDGMAGCIKDECAHRACPISLGKVVEGRVQCPYHGWEYT    55
PhCAO     GMAD-PLGFELFDQCWVLFRDDQGTAACILDECAHRACPLSLGKVIQGRIQCPYHGWEYD    89
MpCAO2    NKGDAATSFVLFGERWELVADDDAAVAAAKTAVGVFG-P-EYAETQAHLVDGAAQRWT--    116

MpCAO1    SGGECKKMPSIKNLLPNVYVDAAPIVERDGLLYVWAGVWEPERAEEILSELPPSAATAPP    115
PhCAO     RQGECVHMPSCQAISNP--ILTLPVMEQGGMIWVWPGTDEPGA--------LPSLAPTLP    139
MpCAO2    ----------CRSRDDA--TRFLPIGLQDGLVM--PDVA------------LP-TTFTPP    149

MpCAO1    SGFAAMAE-VTVDVPLDAPAILSRLMDENKVPFTRVDTTTLSDDVFP--KVIA-------    165
PhCAO     DNFTLQAE-LVMDLEVEHGLMLENLLDLAHAPFTHTGTFAKGWPVPPFVRFANAAT----    194
MpCAO2    AGYTTHAELIIEDVPVEHGLLMENLLDLAHAPFTHTGTFAKGWGVPTFVEFVTSKLRREG    209

MpCAO1    -----------------KVLRGFGKPAPKRVEFTPACILDSTIGLDGVGG----------    198
PhCAO     -----------------TPWTGHWDPYPIHMTFEPPCFVISTIGLR--------------    223
MpCAO2    DGWQDMARGLTREGIGLGSQQGSWNPYPIDMKFVTPCMVDSHIGMSQAGAAGKGAQFEEG    269

MpCAO1    -----QDWNVHQTHVVLPSRPGKARVLYRLSVDFVVGAEIARTVGGQVWQNLAEMILQEQ    253
PhCAO     --GKDCGRHLHQVHACLPRGQGRTRLLYRLALDFGHWLRWVPG-THCLWQHLANRVIQED    280
MpCAO2    VQCAECSNHLHQLHVCVPSEPGRTRLLYRMALDFAGWAKYVPG-IELVWTEMANQVLGED    328

MpCAO1    LEGIRGGR--------FEDDSVGEQAADVSQSYDEWMEEIQAPR----------------    289
PhCAO     LRLVQGQQERLKGGANVWNQPVGY--DKLGVAYRHWRNQVERHGSDWPESPADEGREPAL    338
MpCAO2    LRLVTGQQDRMRRGGRVWAHPVAY--DKLGLVYRRWRNFSVGEACDVSAGIGAEGAGETA    386

MpCAO1    ----------------------------  289
PhCAO     NAIVTGSDAPITGSVVSLPPSQAPPTGH  366
MpCAO2    QRSR------------------------  390
```

Supplementary Figure 2. A sequence alignment of the different CAO homologs used in this work. The first sequence alignment is of *Prochlorothrix hollandica* (*Ph*CAO), *Arabidopsis thaliana* (*At*CAO), and *Chlamydomonas reinhardtii* (*Cr*CAO). The second sequence alignment is of *Ph*CAO with *Micromonas pusilla* (*Mp*CAO). In both alignments, the Rieske cluster binding residues are shown in green and the mononuclear iron binding residues are show in purple. These metallocenters are found in the catalytic domains of the CAO proteins. In the first alignment, the peach and blue colors correspond to the regulatory and linker domains, respectively[1].

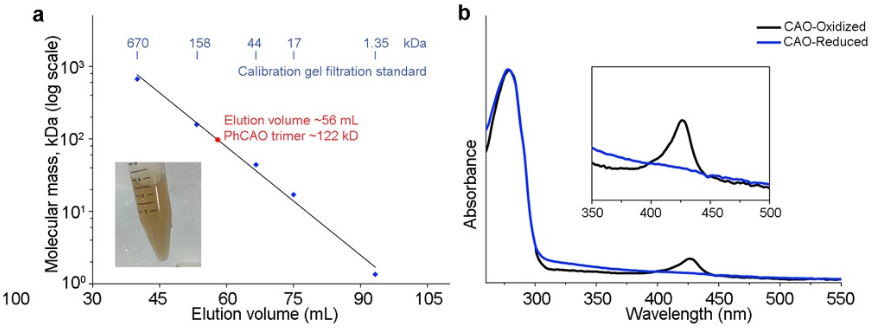

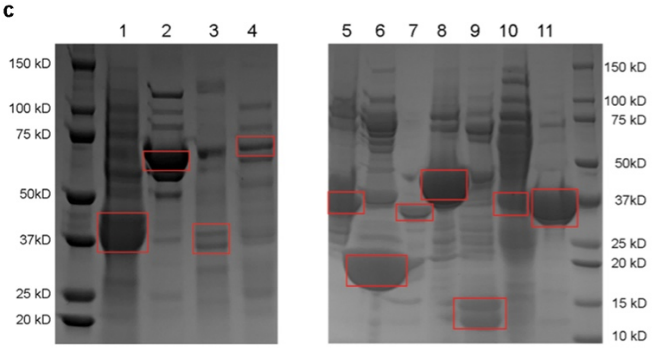

Lane #	Protein	Predicted Size (KDa)
1	*P. hollandica* CAO	40.8
2	*A. thaliana* CAO	60.3
3	*M. pusilla* CAO 1&2	31.7&42.4
4	*C. reinhardtii* CAO	72.1
5	VanB	35.9
6	*E. coli* flavodoxin reductase	19.7
7	*E. coli* flavodoxin	27.7
8	DdmA	44.6
9	DdmB	12.4
10	TsaB	34.2
11	Chlorophyllase	35.7

Supplementary Figure 3. Purification of CAO homologs. **a,** The molecular weight of native *Ph*CAO from the gel filtration chromatography step was estimated using a calibration curve (logarithm of molecular mass versus elution volume) generated from a Bio-Rad gel filtration standard (blue diamonds). **b,** The UV-Vis absorption spectra of the oxidized [2Fe-2S] Rieske cluster of as isolated CAO (black trace) was measured as was the absorption spectra in the presence of the chemical reductant sodium dithionite (blue trace). **c**, An SDS-PAGE gel of the proteins purified in this study. Each purified protein band is outlined in a red box. The predicted protein sizes were determined by the ProtParam tool on the Expasy server and are indicated in the table on the right.

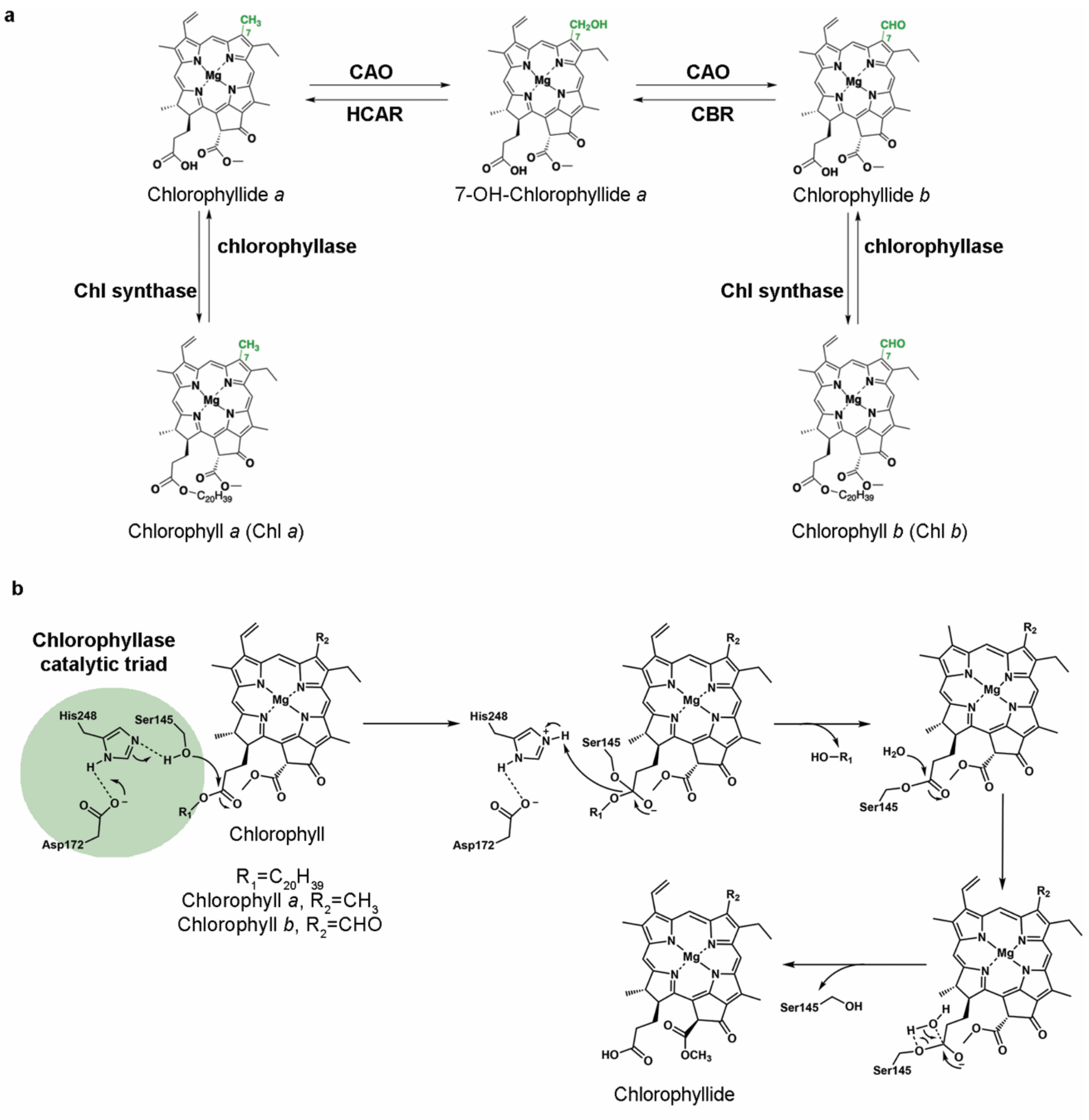

Supplementary Figure 4. Enzymes involved in Chl *b* metabolism. **a,** Chlorophyllase, a degradative enzyme hydrolyzes Chlorophyll into Chlorophyllide by removing the phytol group. Chlorophyllase was used in this work to produce the needed Chlide molecules. Chlorophyll synthase appends a phytol tail to the Chl scaffold. The proposed reactions that constitute the so-called "Chlorophyll cycle" are shown with Chlide for simplicity. Formation of the C7 formyl group in Chlorophyll(ide) *b* is proposed to proceed via two sequential reactions that transform the C7-methyl group of Chlorophyll(ide) *a* into the formyl group of Chlorophyll(ide) *b* through a C7-hydroxymethyl intermediate (7-OH-Chlorophyll(ide) *a*). Chlorophyll(ide) *b* reductase (CBR) and 7-hydroxymethyl-Chlorophyll a reductase (HCAR) are proposed to reduce Chlorophyll(ide) *b* back to Chlorophyll(ide) *a* to fulfill the cycle. **b,** A mechanistic proposal for the chlorophyllase catalyzed removal of the phytol tail from Chlorophyll. The catalytic triad of chlorophyllase, which is an annotated serine protease, is highlighted in a green circle.

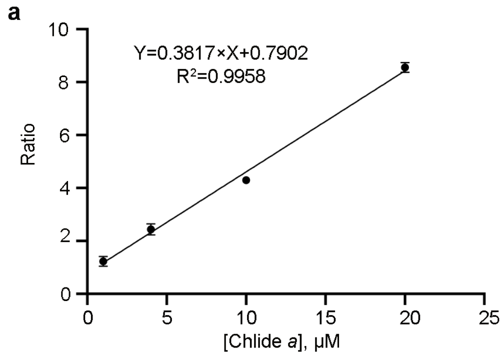

Supplementary Figure 5. A standard curve was used to convert the Q-TOF LC-MS peak area into the amounts of pigments that were produced and consumed in the chlorophyllase-catalyzed reactions. **a**, A standard curve for Chlorophyllide *a*. **b**, A standard curve for Chlorophyllide *b*. In both panels, the x-axis is the concentration of each Chlorophyllide compound whereas the y-axis is the ratio of the peak area integration of the Chlorophyllide compound to the 1,3,5-trimethoxybenzene (0.5 mM) internal standard. All data points were measured in duplicate.

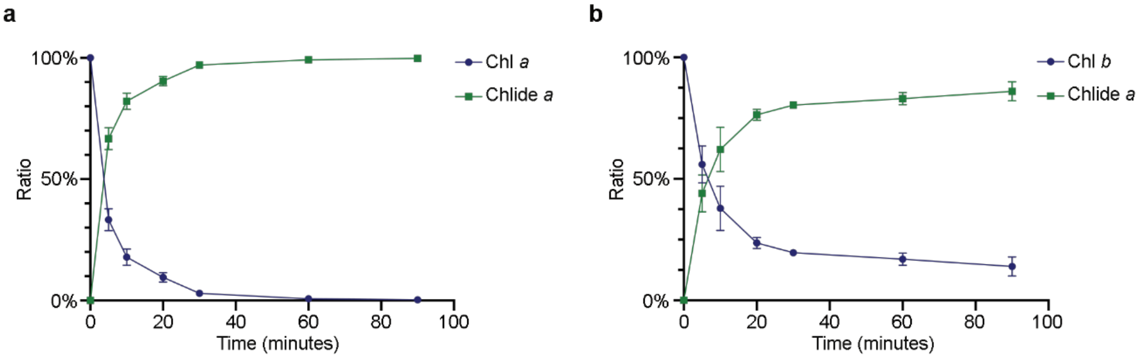

Supplementary Figure 6. Chlorophyllase can be used as a tool to produce the needed Chlide *a* and Childe *b* standards. **a**, A time dependent measurement of Chl *a* (1 mM, blue) consumption and Chlide *a* production using chlorophyllase (1.5 μM, green). The chlorophyllase reaction reaches the maximum amount of substrate conversion at 1 hour, which corresponds to conversion of nearly 100-percent Chl *a* into Chlide *a*. **b**, A time dependent measurement of Chl *b* (1 mM, blue) consumption and Chlide *b* production using chlorophyllase (1.5 μM, green). The chlorophyllase reaction reaches the maximum amount of substrate conversion at 1.5 hours. At this point, nearly 85-percent of the Chl *b* substrate has been converted into Chlide *b.* For both panels, the data points were measured in duplicate.

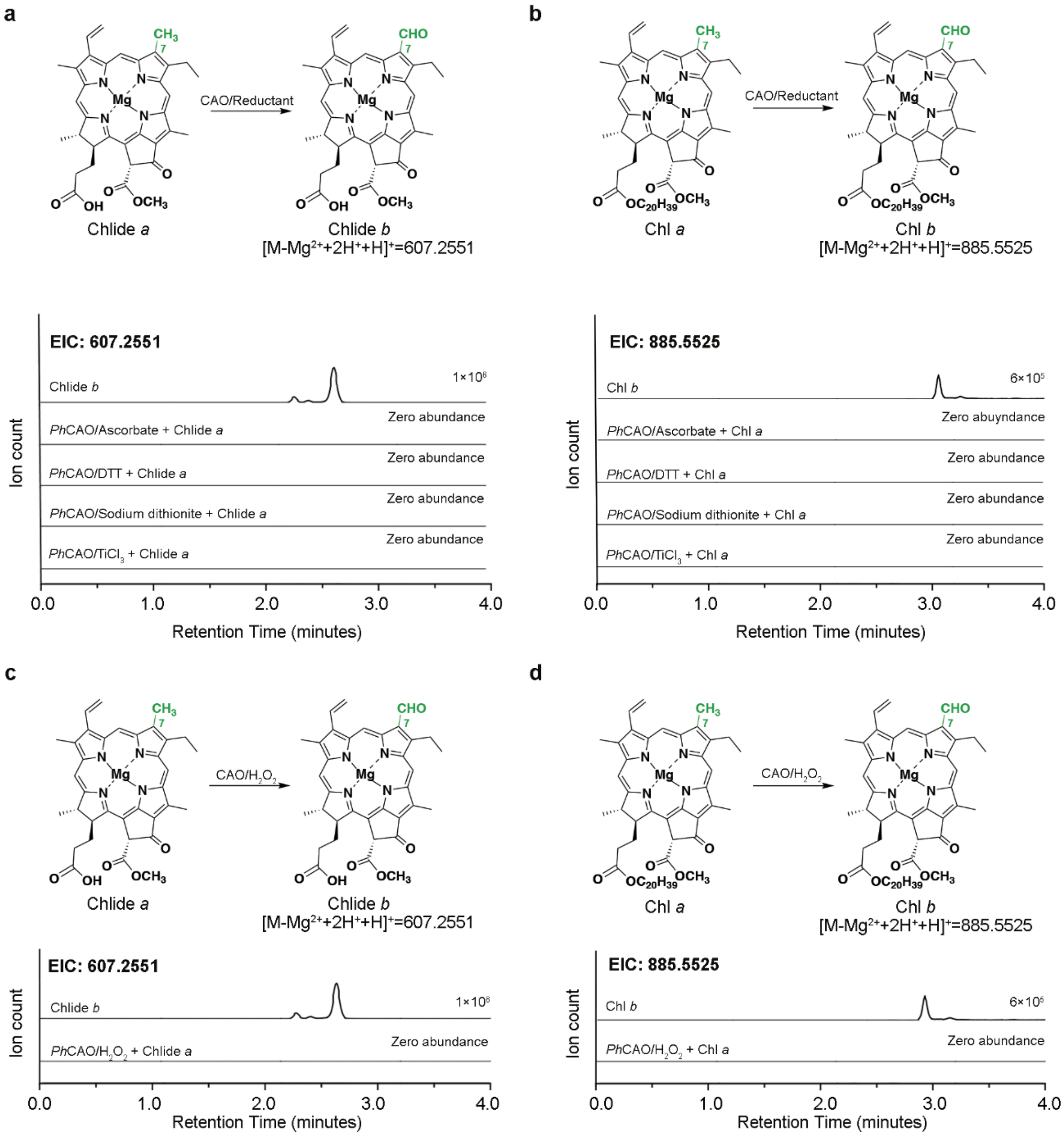

Supplementary Figure 7. Several chemical reductants and the peroxide (H_2O_2) shunt reaction were tested in the CAO assays. **a**, CAO is unable to convert Chlide *a* into Chlide *b* using ascorbate, dithiothreitol (DTT), sodium dithionite, or titanium (III) chloride ($TiCl_3$) as a reductant. **b**, Similarly, CAO does not catalyze the conversion of Chl *a* into Chl *b* using a chemical reductant. **c**, CAO is unable to transform Chlide *a* into Chlide *b* using H_2O_2 to bypass the need for an electron donor. **d**, Likewise, CAO does not show any activity on Chl *a* with H_2O_2. In the extracted ion chromatograms the m/z of 607.2551 and 885.5525 represent the mass of $[M+H]^+$ of Chlorophyllide *b* and Chlorophyll *b* minus Mg^{2+} plus 2 H^+, respectively. All data was performed in triplicate.

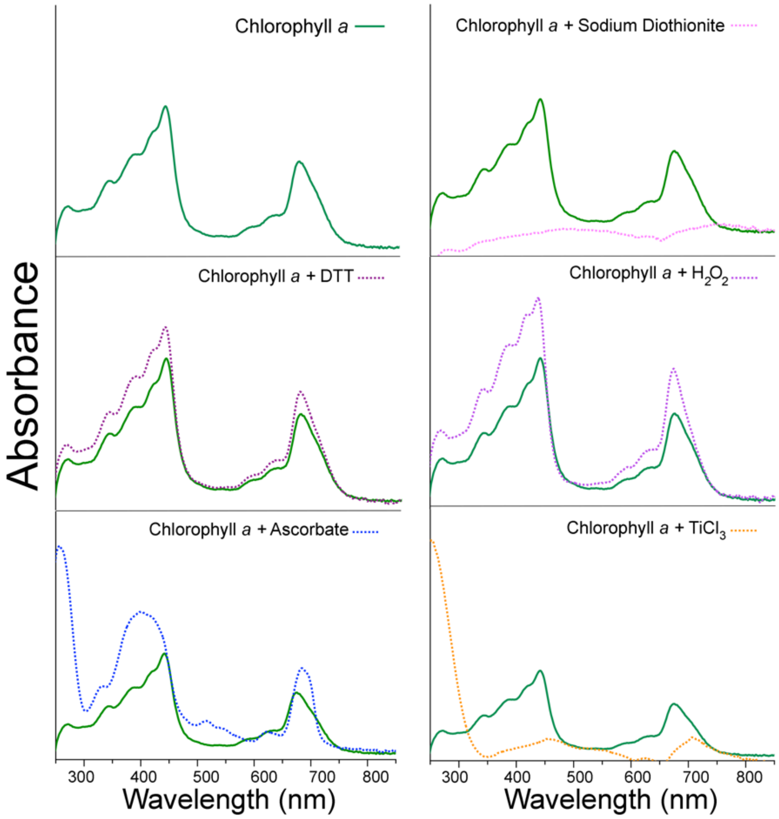

Supplementary Figure 8. The addition of 1 mM sodium dithionite, ascorbate, or $TiCl_3$ to 20 μM Chlorophyll *a* result in notable changes to the distinctive absorption peaks**.**

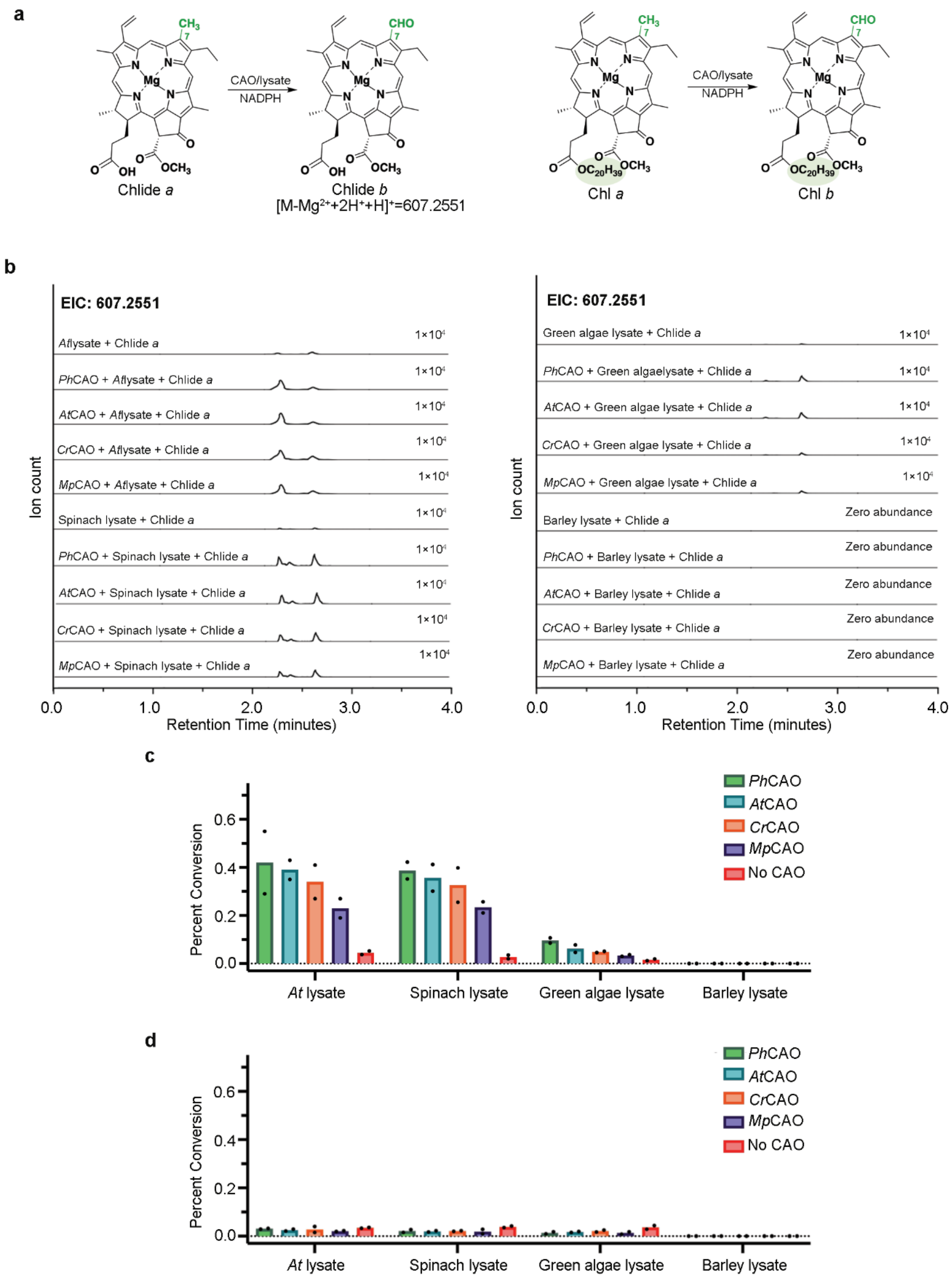

Supplementary Figure 9. Cell lysate from four different photosynthetic organisms was tested for its ability to support Chlide *b* production. **a**, Reaction scheme for the CAO catalyzed conversion of Chlide *a* into Chlide *b* or Chl *a* into Chl *b* in different cell lysates. **b**, The extracted ion chromatograms of the CAO-catalyzed reactions performed in cell lysate with a Chlide *a* substrate.

c, A bar chart to compare the activity of CAO homologs in different cell lysates using Chlide *a* as the substrate. Of note, *Arabidopsis thaliana* cell lysate allows for the highest production of Chlide *b* for each of the different CAO homologs. Spinach lysate shows similar level activity compared with *At* lysate. Green algae lysate, on the other hand, shows much lower activity. Finally, barley lysate failed to support the ability of any CAO homolog to produce Chlide *b*. **d**, A bar chart to compare the activity of CAO homologs with different cell lysates using Chl *a* as the substrate. None of the tested cell lysates shows the ability to support any of the CAO homologs to transform Chl *a* into Chl *b*. All data in the bar graph was measured in duplicate and are shown as mean values ± SD.

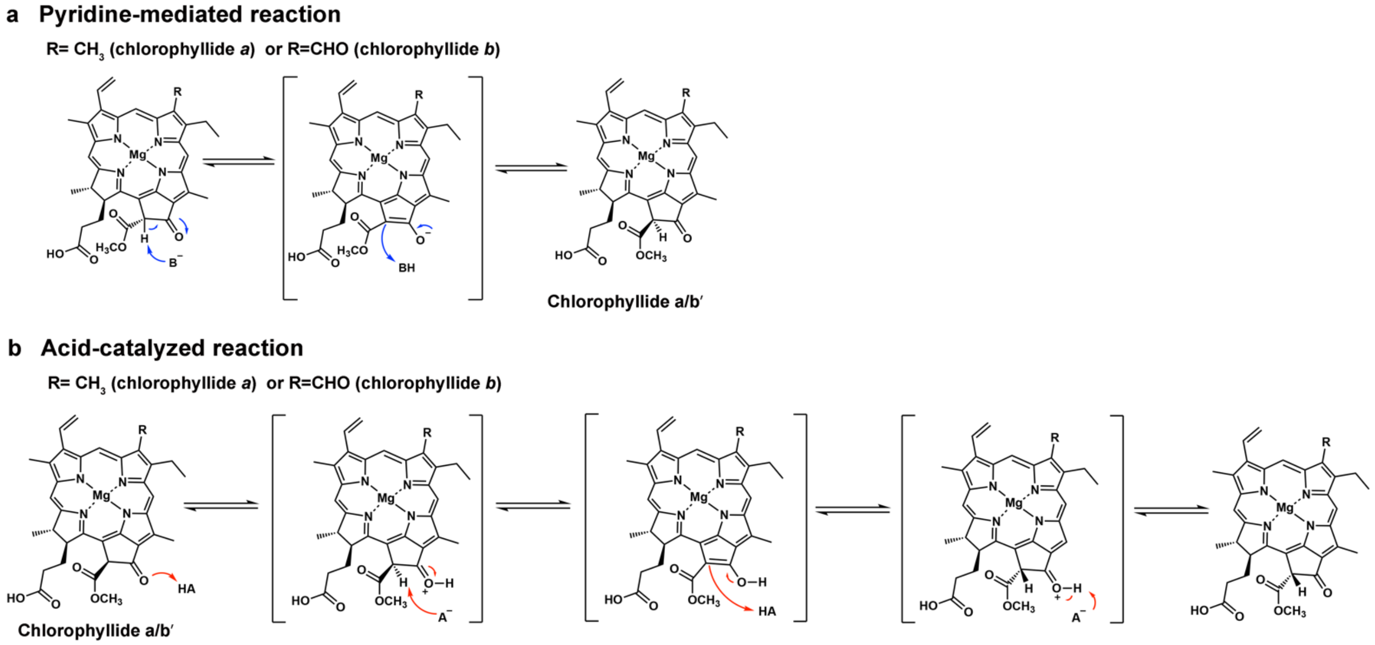

Supplementary Figure 10. The stereochemistry at the C-13^2 position of Chl pigments can be changed using acid-base chemistry. (a) Proposal for the pyridine catalyzed conversion of Chlorophyllide *a* or *b* into Chlorophyllide *a*′ or *b*′. Here, pyridine abstracts the proton from the C-13^2 position of Chl to form an enolate anion. (b) Proposal for the acid (HCl) catalyzed conversion of Chlorophyllide *a*′ or *b*′ into Chlorophyllide *a* or *b*.

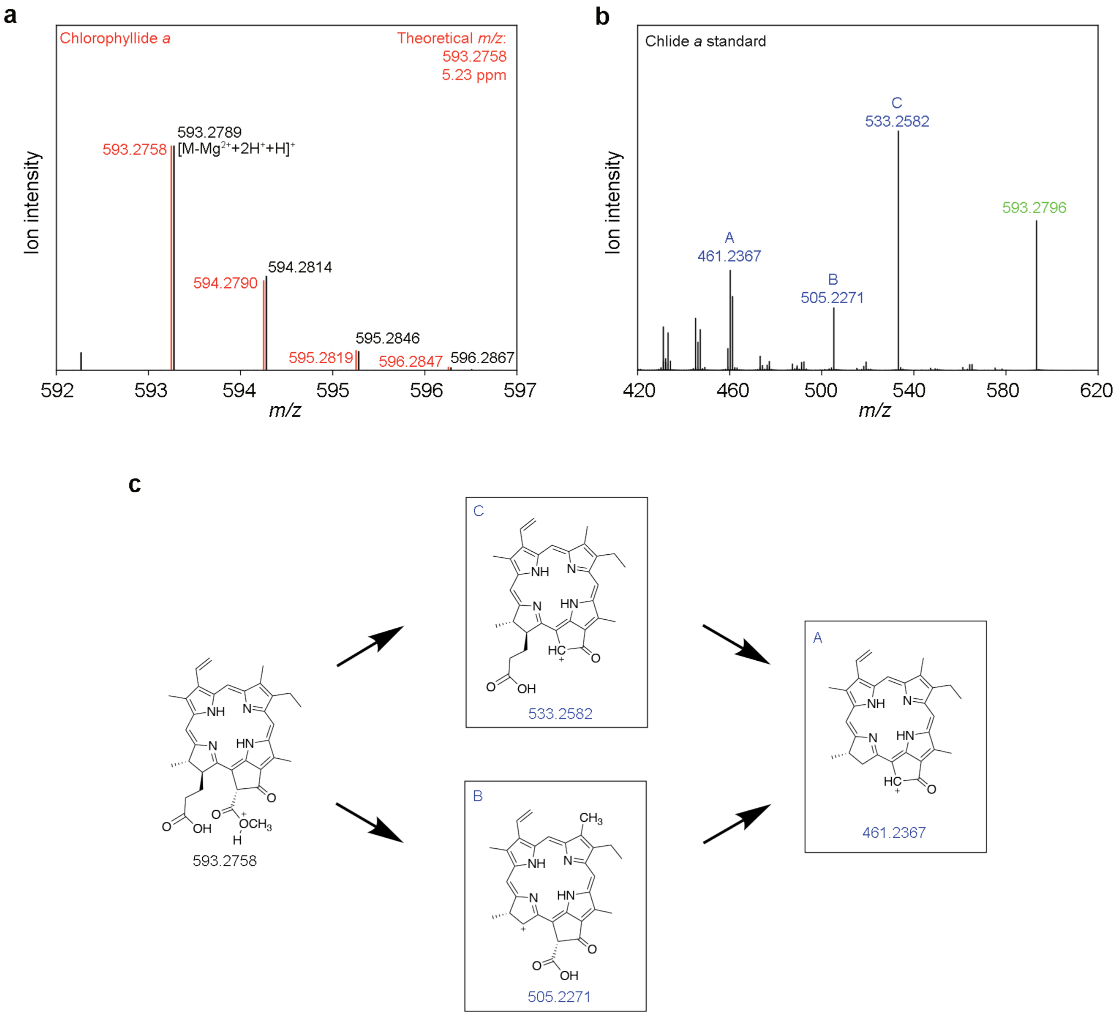

Supplementary Figure 11. Analysis of the enzymatically produced Chlide *a* standard. **a**, The predicted (red) and the observed (from the chlorophyllase reaction, black) isotope distribution for Chlide *a* show good agreement. **b**, MS/MS spectrum of the Chlide *a* standard shows three major fragments of interest, labelled as A, B and C. **c**, The proposed fragmentation pathway for Chlide *a*. Of note, the major fragments (C and A) have a mass loss of 60.0176 Da ($C_2H_4O_2$) and 132.0391 ($C_5H_8O_4$), suggesting that the side chains on C13^2 and C17 are relatively easy to dissociate.

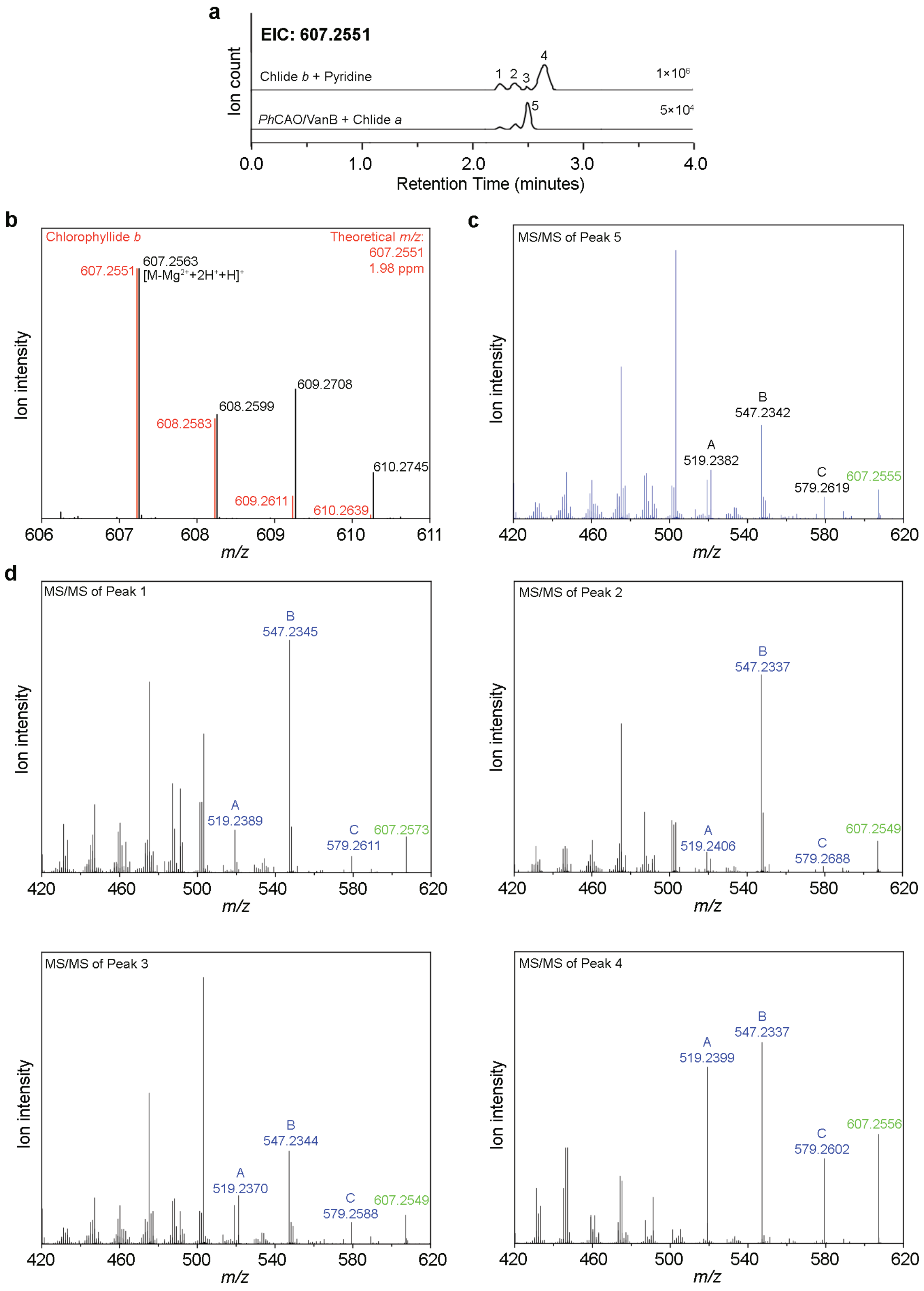
a
EIC: 607.2551
Ion count
Chlide b + Pyridine
1 2 3 4
1×10^6
PhCAO/VanB + Chlide a
5
5×10^4
0.0 1.0 2.0 3.0 4.0
Retention Time (minutes)
b
Chlorophyllide b
Theoretical m/z: 607.2551 1.98 ppm
607.2551
607.2563 [M-Mg2++2H++H]+
608.2583
608.2599
609.2708
609.2611
610.2745
610.2639
Ion intensity
606 607 608 609 610 611
m/z
c
MS/MS of Peak 5
B 547.2342
A 519.2382
C 579.2619
607.2555
Ion intensity
420 460 500 540 580 620
m/z
d
MS/MS of Peak 1
B 547.2345
A 519.2389
C 579.2611
607.2573
MS/MS of Peak 2
B 547.2337
A 519.2406
C 579.2688
607.2549
MS/MS of Peak 3
B 547.2344
A 519.2370
C 579.2588
607.2549
MS/MS of Peak 4
B 547.2337
A 519.2399
C 579.2602
607.2556
Ion intensity
420 460 500 540 580 620
m/z

e

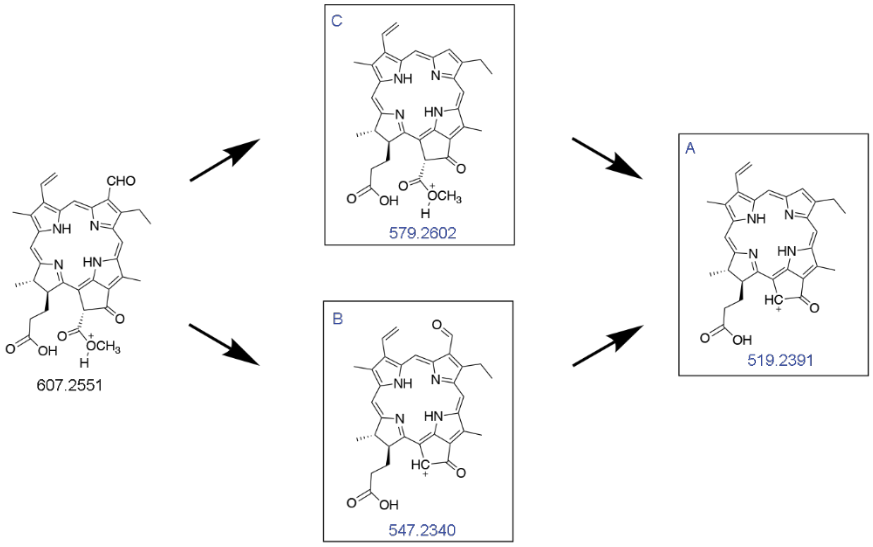

Supplementary Figure 12. Analysis of the enzymatically produced Chlide *b* standard and the product of the CAO reaction. **a**, Extracted ion chromatograms for the Chlide *b* standard and product of the *Ph*CAO reaction. There are four peaks shown in the Chlide *b* standard (labeled peaks 1-4) and the product peak of the *Ph*CAO reaction (peak 5) has the same retention time as peak 3 from the standard. **b**, The predicted (red) and observed (from the *Ph*CAO reaction, black) isotope distribution for Chlide *b* show good agreement. **c**, The MS/MS spectrum of peak 5 from the *Ph*CAO reaction. **d**, The MS/MS spectrum of peaks 1-4 from the Chlide *b* standard. Each of the peaks 1-4 show different fragmentation patterns consistent with them being diastereomers of Chlide *b*. Comparison of the retention time (panel a) and MS/MS spectrum of the product peak of the CAO reaction (panel c) with the Chlide *b* standard (panel d), suggests that the major product of the CAO reaction shows the same diastereomer configuration as peak 3 in the Chlide *b* standard. **e**, A proposed fragmentation pathway for Chlide *b* that gives rise to the fragments that are labeled A, B, and C, and are observed in all five MS/MS spectra. Fragment C, which demonstrates a mass loss of 27.9949 Da corresponds to the loss of a formyl group.

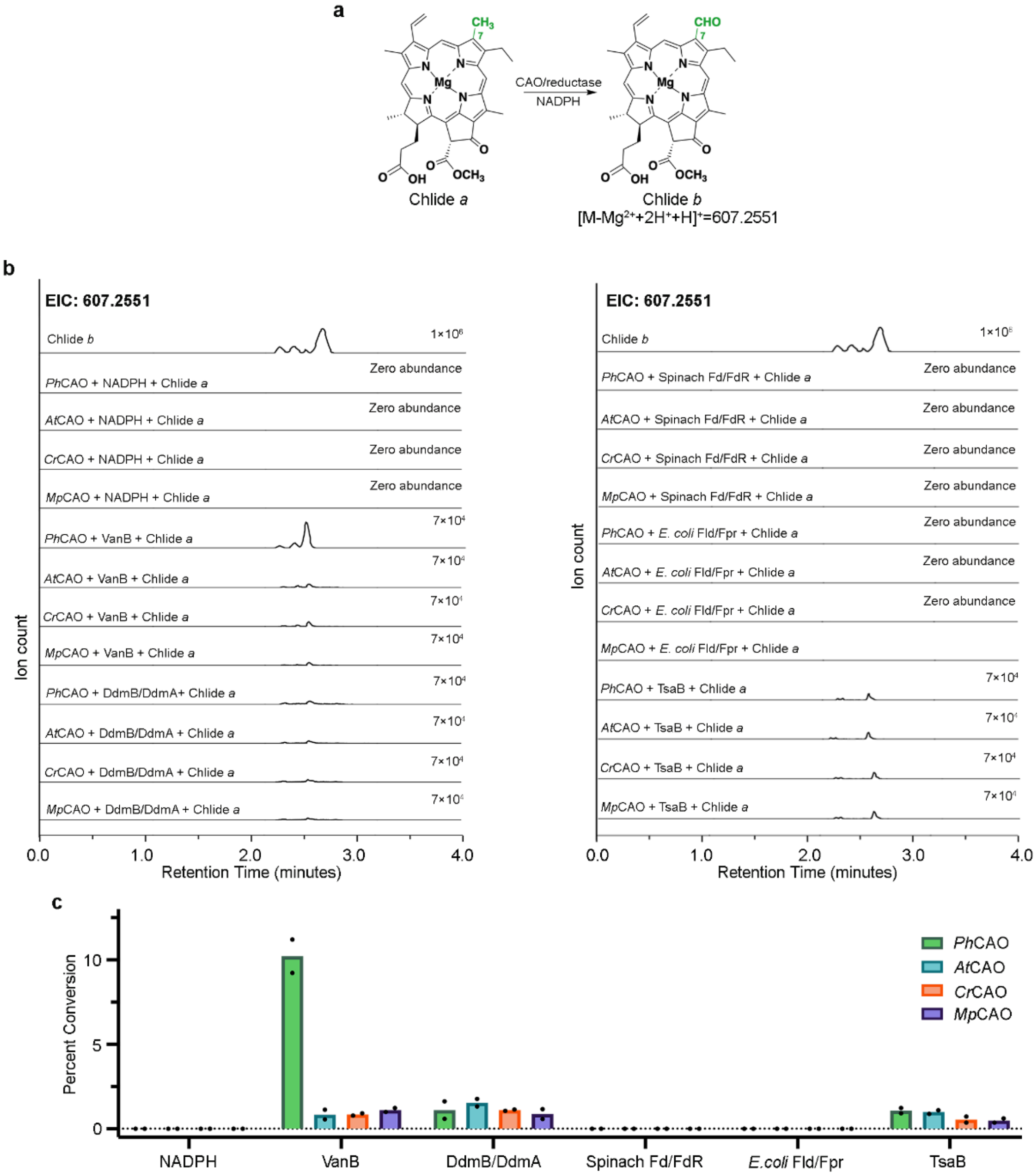

Supplementary Figure 13. Five different non-native reductase systems were tested for their ability to support Chlide *b* production. **a**, Reaction scheme of the CAO catalyzed conversion of Chlide *a* into Chlide *b* with NADPH and different reductase systems. **b**, The extracted ion chromatograms of the products from the CAO reactions that were performed using NADPH and five different reductases (VanB, DdmB/DdmA, Spinach ferredoxin/ferredoxin reductase, *E. coli* flavodoxin/flavodoxin reductase, and TsaB). **c**, A bar chart to compare the activity of the CAO homologs with NADPH and the different tested reductases. VanB, the reductase partner of a Rieske oxygenase, vanillate *O*-demethylase, shows the highest activity among all tested reductase systems. All data in the bar graph was performed in duplicate and are shown as mean values ± SD.

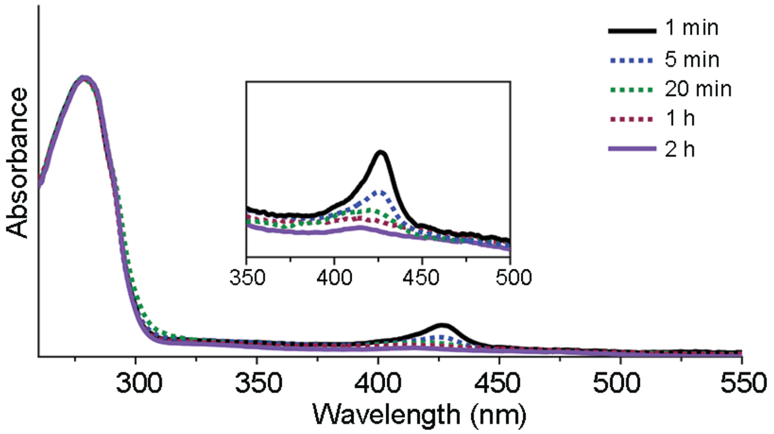

Supplementary Figure 14. The UV-Vis absorption spectra of the Rieske [2Fe-2S] cluster in *Ph*CAO shows that it can be reduced by the non-native reductase VanB over time.

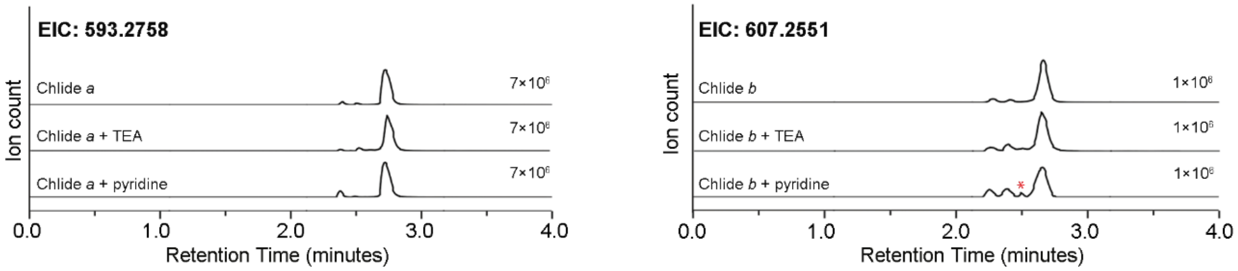

Supplementary Figure 15. The diastereomer distribution of Chlide *a* (left) and Chlide *b* (right) can be shifted by triethylamine (TEA) or pyridine. The peak observed in the Chlide *b* with pyridine reaction that matches the product peak in CAO reaction is labelled with a red asterisk. The m/z=593.2758 and m/z=607.2551 represents the $[M+H]^+$ of Chlide *a* and Chlide *b* minus Mg^{2+} plus $2H^+$, respectively.

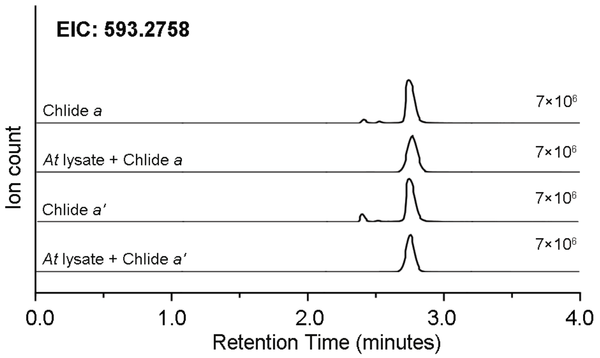

Supplementary Figure 16. The diastereomer equilibrium of the Chlide *a* and Chlide *a′* can be shifted by *A. thaliana* cell lysate.

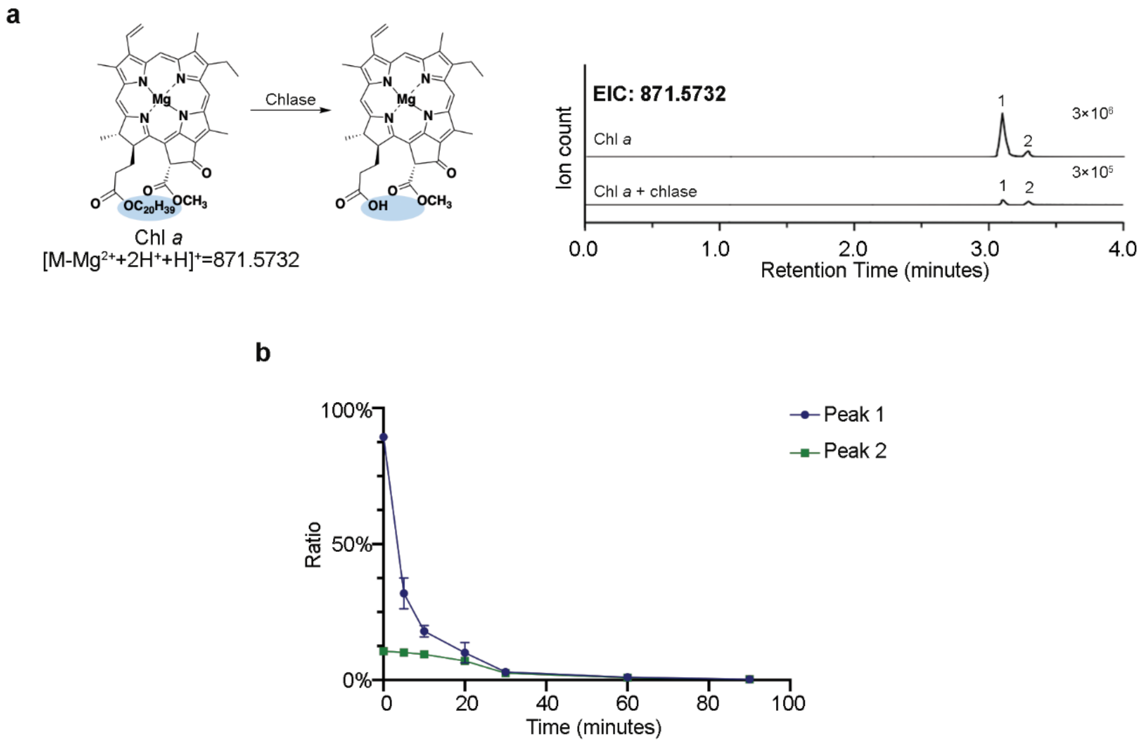

Supplementary Figure 17. A certain diastereomer of Chl *a* is preferred by chlorophyllase. **a**, Reaction scheme and the extracted ion chromatograms of the substrate (Chl *a*) from the hydrolysis reaction catalyzed by chlorophyllase (abbreviated chlase). **b**, A time dependent measurement of the consumption of the two diastereomers present in the Chl *a* standard. The diastereomer of peak 1 is preferred by chlorophyllase although both diastereomers are mostly consumed after the 1 hour incubation.

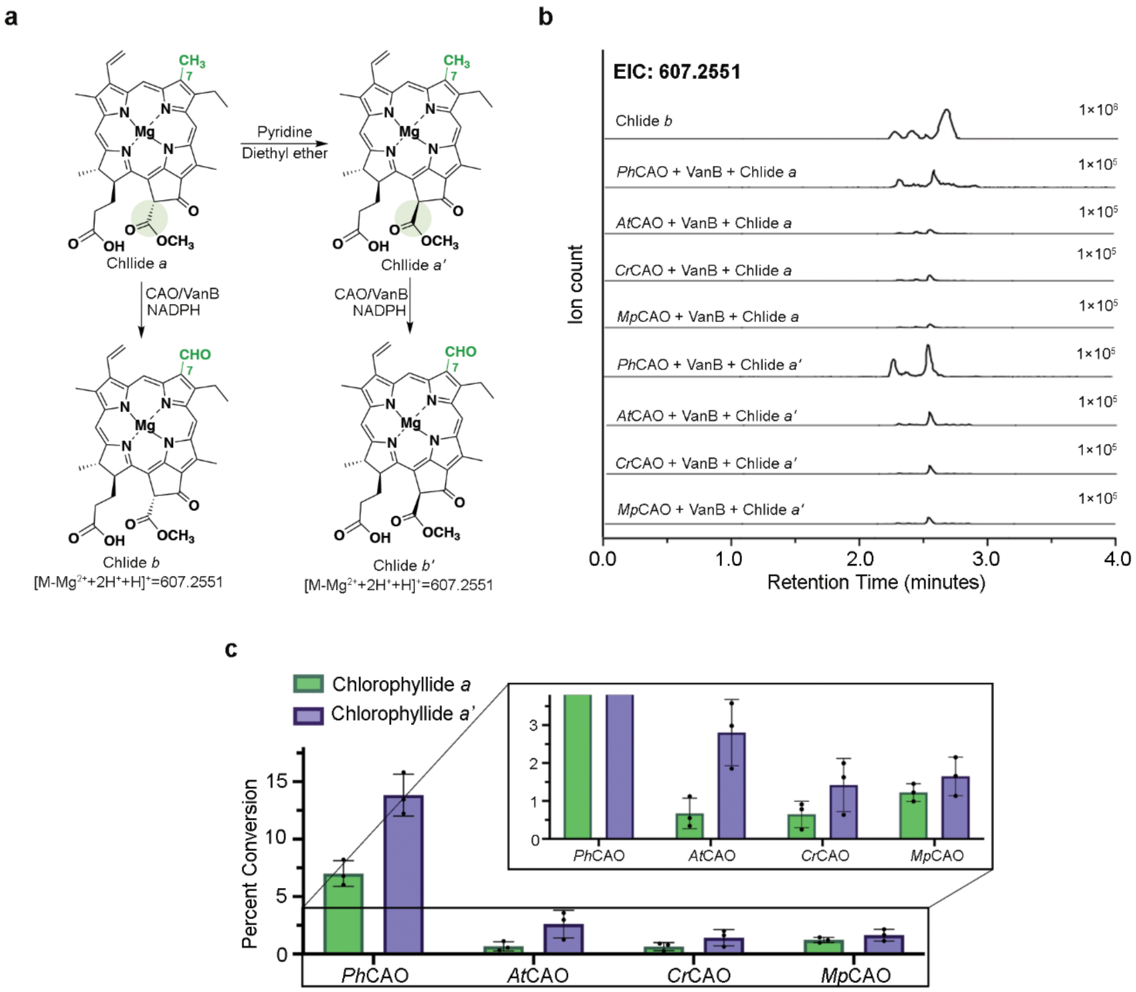

Supplementary Figure 18. The Chlide *a*′ diastereomer is preferred by all tested homologs of CAO. **a**, Reaction scheme that shows the pyridine-mediated transformation of Chlide *a* into Chlide *a*′. **b**, The extracted ion chromatograms of the CAO reaction product using either Chlide *a* or Chlide *a*′ as a substrate. **c**, A bar chart to compare the percent yield of CAO homologs with Chlide *a* and Chlide *a*′. This data revealed that all four homologs show a preference for Chlide *a*′ rather than Chlide *a*. All data points were measured in triplicate and are shown as mean values ± SD.

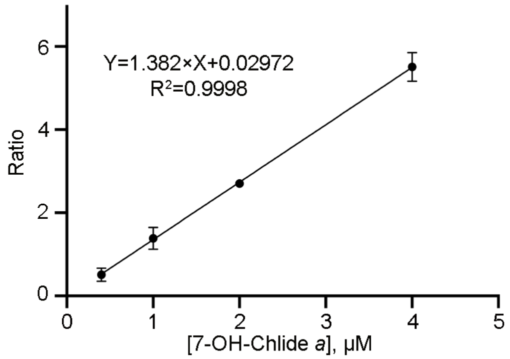

Supplementary Figure 19. A standard curve for the Q-TOF LC-MS data was used to determine the amount of intermediate (7-OH-Chlorophyllide *a*) in the reactions. The x-axis is the concentration of 7-OH-Chlorophyllide *a* whereas the y-axis is the ratio of the peak area integration of the 7-OH-Chlorophyllide *a* to the 1,3,5-trimethoxybenzene (0.5 mM) internal standard. All data points were measured in duplicate.

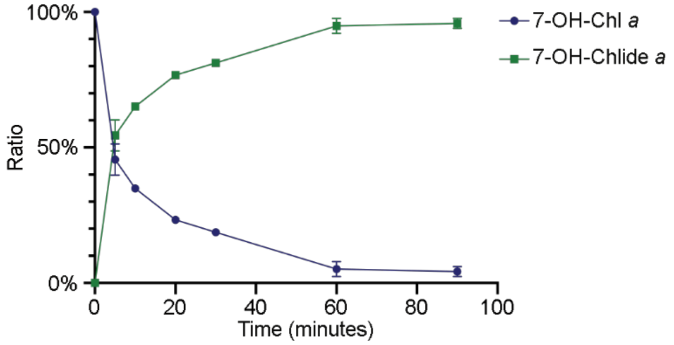

Supplementary Figure 20. Chlorophyllase can be used as a tool to produce a 7-OH-Chlide *a* standard. A time dependent measurement of 7-OH-Chl *a* (1 mM, blue) consumption and 7-OH-Chlide *a* production using chlorophyllase (1.5 µM, green) shows that the chlorophyllase reaction reaches the maximum amount of substrate conversion at 1 hour. At this time point, approximately 95-percent of the 7-OH-Chl *a* has been converted into 7-OH-Chlide *a.* All data points were measured in duplicate.

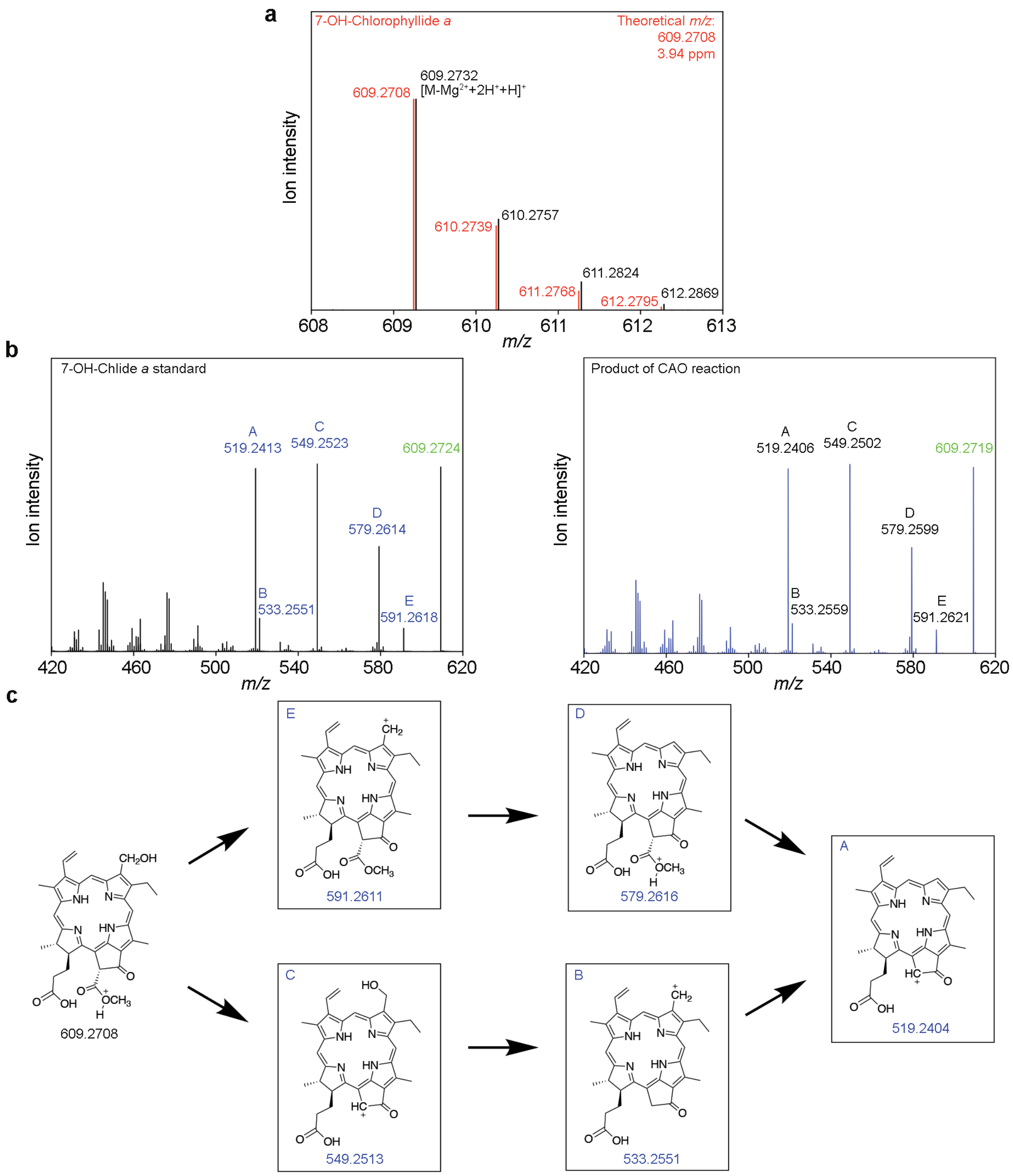

Supplementary Figure 21. Analysis of the chemically produced 7-OH-Chlide *a* standard and the intermediate produced in the CAO reaction. **a**, The predicted (red) and observed (from the *Ph*CAO reaction, black) isotope distribution for 7-OH-Chlide *a* show good agreement. **b**, The MS/MS spectrum of the produced 7-OH-Chlide *a* standard (left panel) is compared to the MS/MS spectrum of the proposed 7-OH-Chlide *a* intermediate from the *Ph*CAO reaction (right panel). **c**, Proposed fragmentation pathway for 7-OH-Chlide *a*. The existence of fragments D and E are consistent with the presence of a hydroxymethyl group in the compound being tested due to the mass loss of 18.0097 Da (H_2O) and 30.0092 Da (CH_2O).

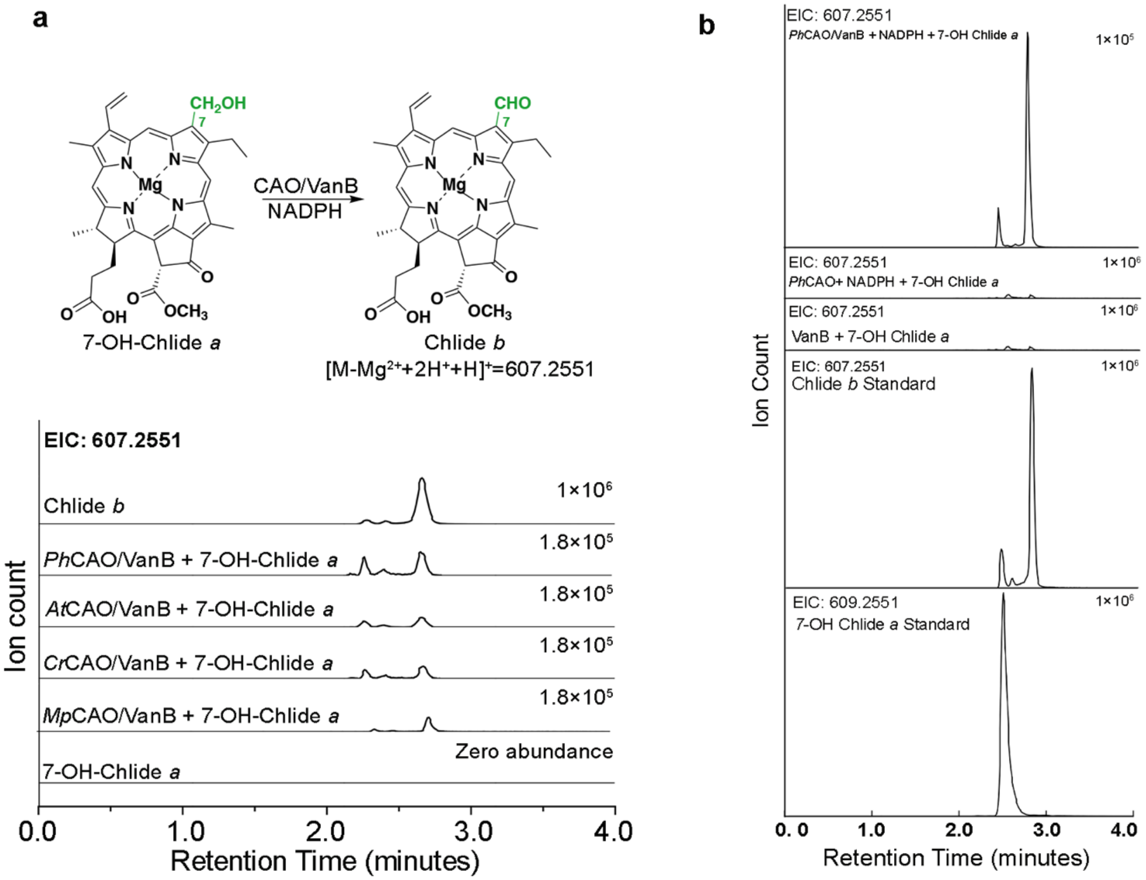

Supplementary Figure 22. Extracted ion chromatograms for the CAO homolog reaction products formed when CAO was combined with VanB, NADPH, and a 7-OH-Chlide *a* substrate. **a**, These traces reveal that 7-OH-Chlide *a* can be converted into Chlide *b*. **b**, These traces reveal that in the absence of CAO or VanB, the expected Chlide *b* product is not formed which means that to convert the intermediate into the product, both enzymes are needed.

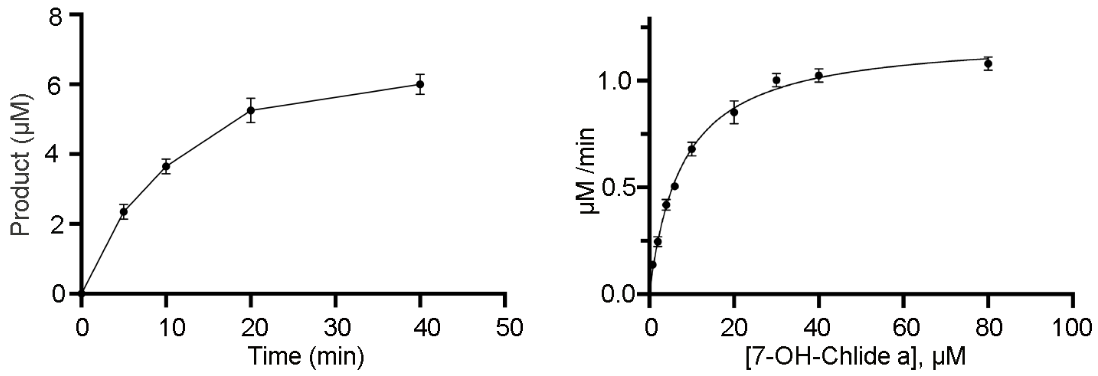

Supplementary Figure 23. Steady state kinetic behavior of *Ph*CAO with a 7-OH-Chlide *a* substrate. A time course of *Ph*CAO with a 7-OH-Chlide *a* substrate was measured to find the linear range of product formation (left panel). This data demonstrates that 5 min is an optimal time to capture the initial reaction rate. The steady-state kinetics of *Ph*CAO with the 7-OH-Chlide *a* substrate was fitted to the Michaelis-Menten equation with K_M= 7.8 ± 0.9 µM and V_{max}=1.2 ± 0.1 µM min^{-1} (right panel). All data points in this figure were measured in duplicate.

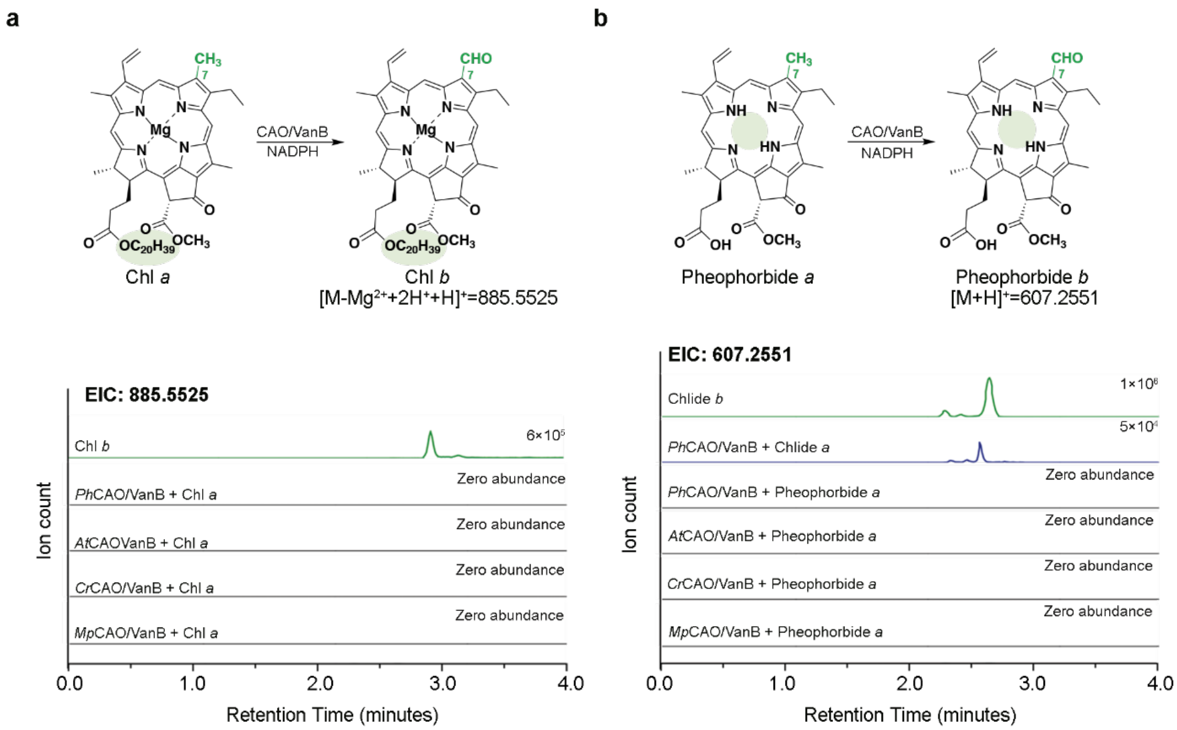

Supplementary Figure 24. Chlorophyll *a* and Pheophorbide *a* are not substrates of the CAO homologs. **a**, An extracted ion chromatogram of the expected product of a CAO-VanB-catalyzed reaction with a Chlorophyll *a* substrate does not reveal production of a hydroxylated product, suggesting the long phytol group of Chlorophyll *a*, relative to Chlide *a*, impairs the activity of CAO. **b**, Similarly, none of the CAO homologs show production of a hydroxylated product when provided with a Pheophorbide *a* substrate, suggesting that the central metal ion (Mg^{2+}) is also important for CAO activity.

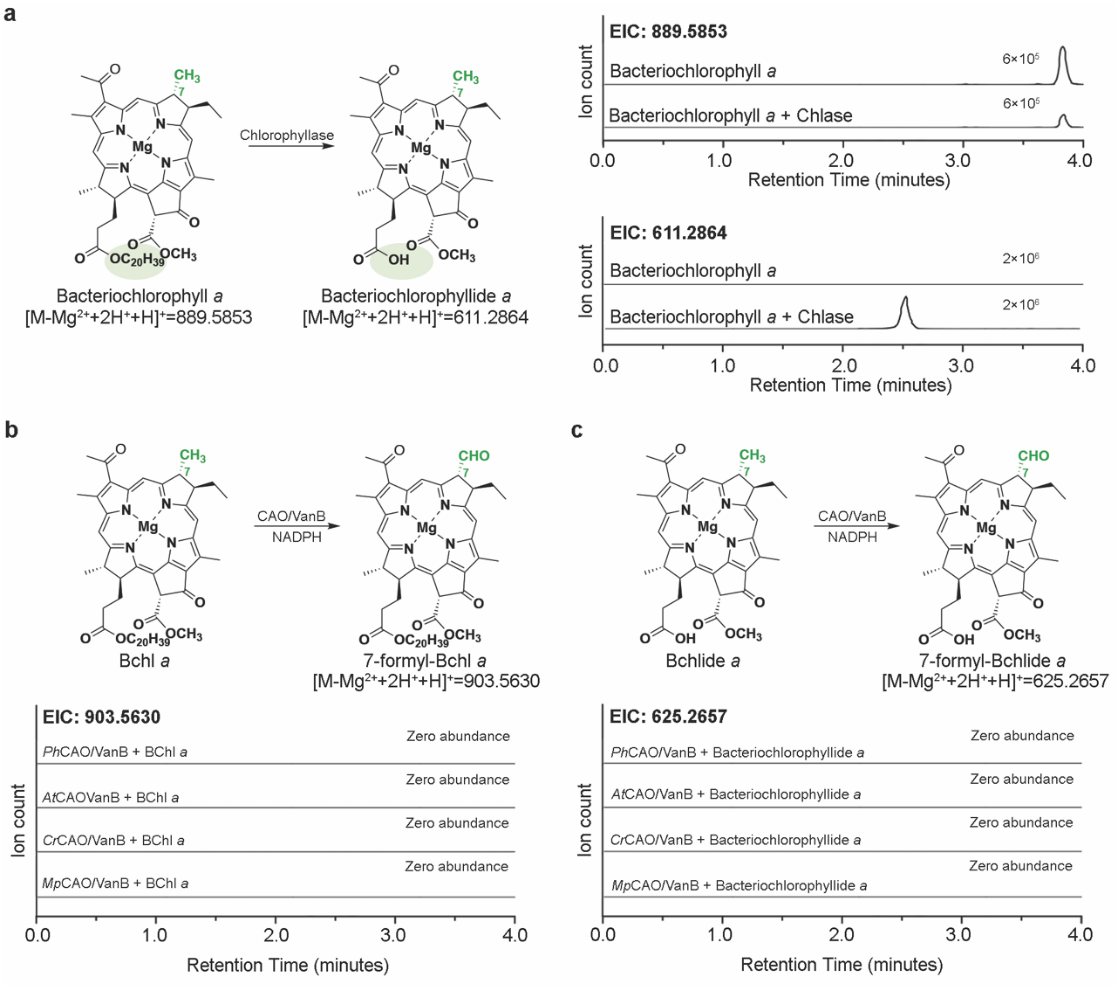

Supplementary Figure 25. The activity of the CAO homologs was tested on Bacteriochlorophyll *a* and Bacteriochlorophyllide *a* substrates. **a**, Chlorophyllase was demonstrated to hydrolyze the phytol tail of Bacteriochlorophyll *a* to produce Bacteriochlorophyllide *a.* **b**, The extracted ion chromatograms of the CAO-VanB reaction products when incubated with a Bacteriochlorophyll *a* substrate reveals that none of the CAO homologs accept Bacteriochlorophyll *a* as a substrate. **c**, Similarly, the CAO homologs do not show activity on Bacteriochlorophyllide *a* using VanB as a reductase. Collectively, these data suggest that substrates that contain a bacteriochlorin scaffold cannot be oxidized by CAO.

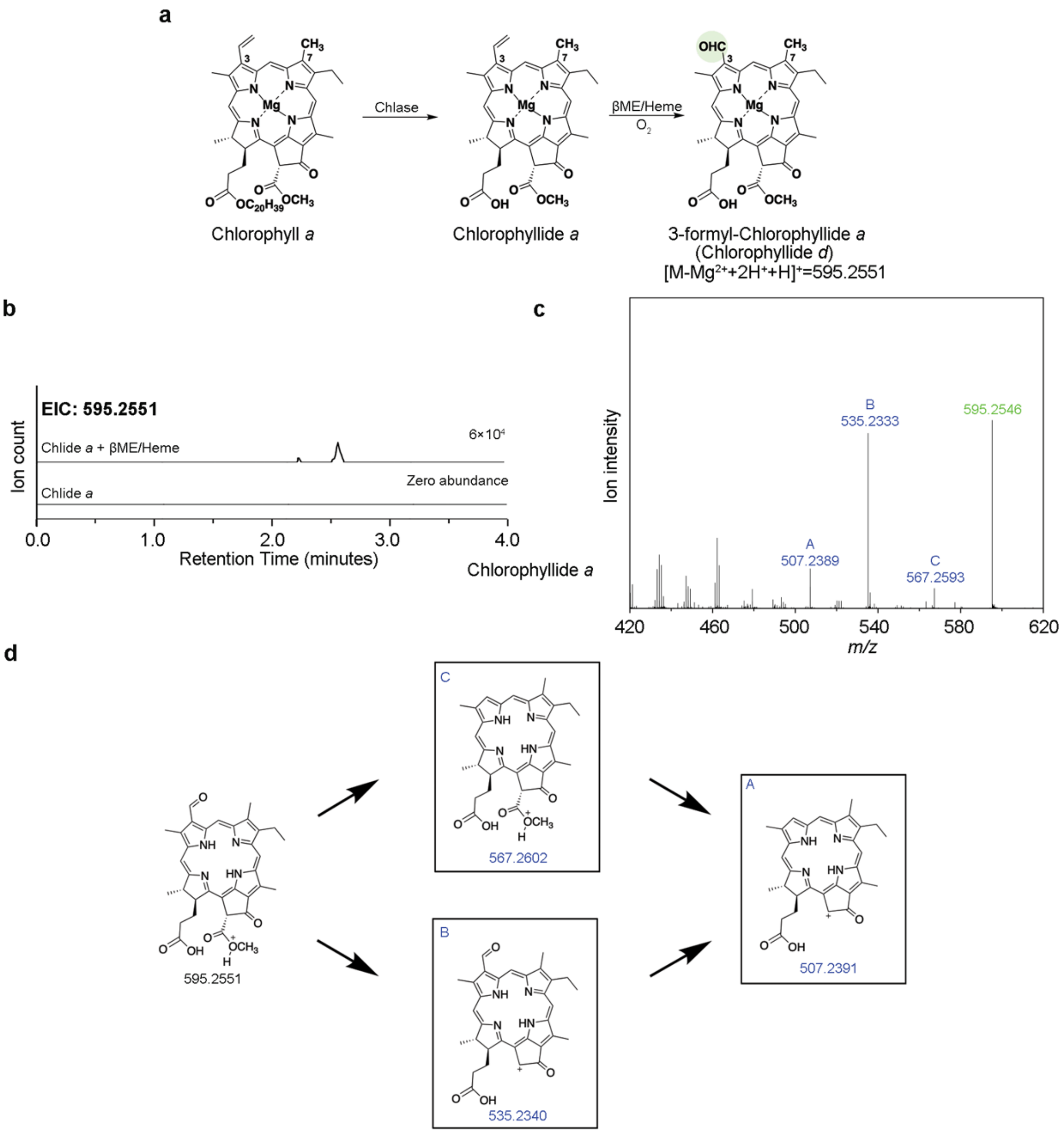

Supplementary Figure 26. Analysis of the chemically produced Chlide *d* (3-formyl-Chlide *a). * **a**, Reaction scheme of enzymatic and chemical synthesis of Chlide *d*. **b**, Extracted ion chromatogram of the product formed by following the published methods for using β-mercaptoethanol (βME) and heme to convert Chlide *a* into Chlide *d*[2]. **c**, MS/MS spectrum Chlide *d* shows three major fragments of interest, labelled as A, B and C. **d**, A proposed fragmentation pathway for Chlide *d*. Fragment C, which demonstrates a mass loss of 27.9949 Da corresponds to the loss of a formyl group.

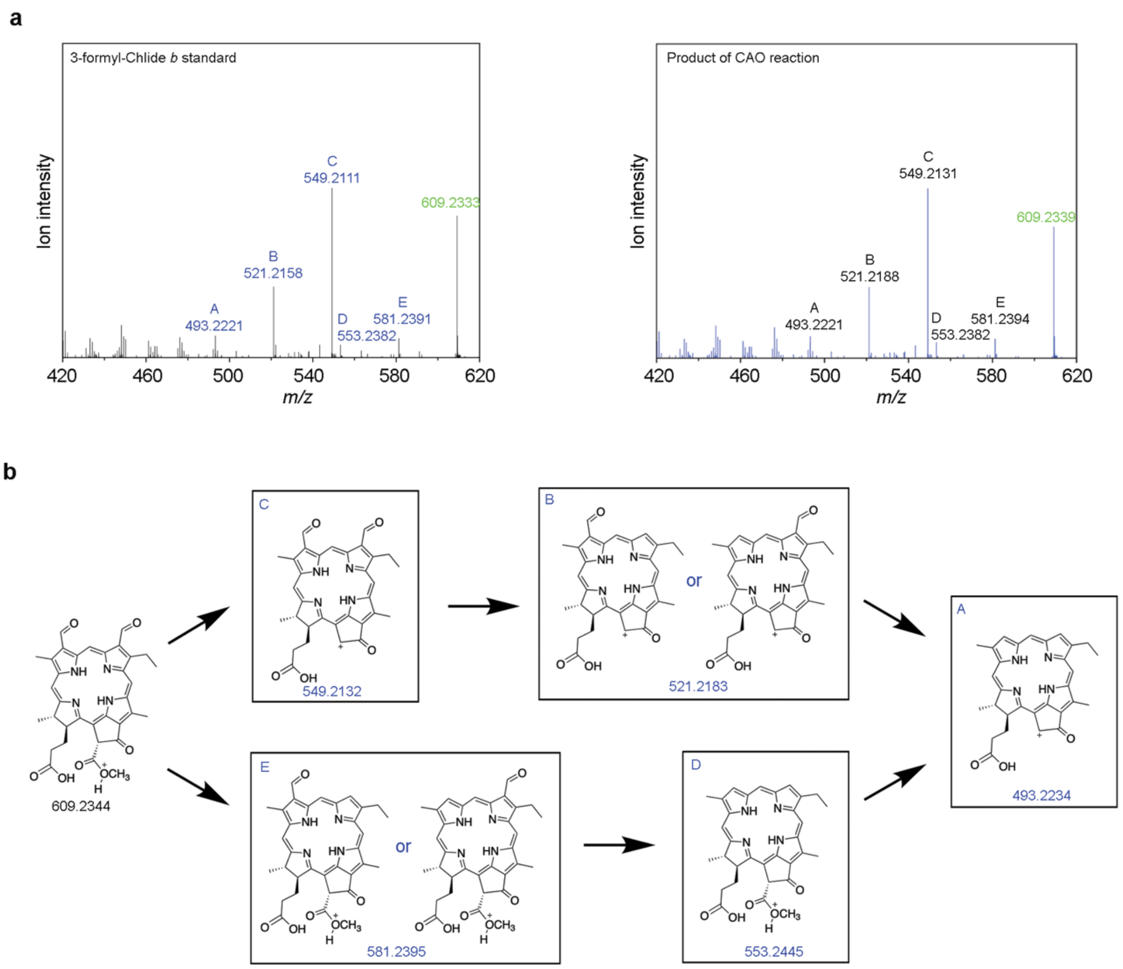

Supplementary Figure 27. Analysis of the 3-formyl-Chlide *b* produced in the CAO reaction. **a**, The MS/MS spectrum of produced 3-formyl-Chlide *b* standard (left panel) is compared to the MS/MS spectrum of the 3-formyl-Chlide *b* from the *Ph*CAO reaction (right panel). **b**, Proposed fragmentation pathway for 3-formyl-Chlide *b*. The existence of fragments D and E are consistent with the presence of two formyl groups in the compound being tested due to the mass loss of 27.9949 Da (CO) and 55.9899 Da (C_2O_2).

II. Supplementary Methods

IIa. DNA and protein sequences

Prochlorothrix hollandica **Chlorophyllide *a* oxygenase (*Ph*CAO):**

UniProt: A0A0M2PSM7

ATGAATAATAGCCTCAACGTATCCGCAACCCTAGATTTAGCGAACGGATTACGCAACTTTTGGTATCCCGTTGA
GTTTTCTAAGAATTTGGGGATGGCGGATCCCCTGGGCTTCGAGTTATTTGATCAATGTTGGGTTTTGTTTCGCG
ATGATCAGGGAACTGCCGCCTGTATTCTGGATGAATGTGCCCACCGCGCTTGTCCCCTATCCCTGGGCAAGGTG
ATCCAAGGGCGGATCCAGTGCCCCTACCATGGTTGGGAGTACGATCGCCAGGGGGAGTGTGTCCATATGCCCTC
CTGCCAAGCCATCAGCAATCCAATTTTGACCTTGCCAGTGATGGAGCAAGGGGGCATGATTTGGGTTTGGCCCG
GAACCGACGAGCCAGGTGCCTTGCCATCCTTAGCCCCCACCCTACCGGACAATTTCACCCTCCAGGCTGAATTG
GTGATGGATTTGGAGGTGGAGCATGGCTTAATGCTGGAAAACCTCTTAGATCTCGCCCATGCCCCCTTTACCCA
CACCGGCACCTTTGCCAAGGGCTGGCCCGTCCCCCCTTTTGTCCGGTTTGCCAATGCTGCCACCACACCCTGGA
CCGGGCACTGGGATCCCTATCCCATCCACATGACCTTTGAACCCCCTTGCTTTGTCATTAGCACCATCGGCTTG
CGGGGCAAAGACTGTGGCCGCCATCTGCATCAGGTTCACGCCTGTCTCCCTCGGGGCCAGGGGCGCACTCGCTT
GCTCTACCGCTTAGCCTTAGATTTTGGCCACTGGTTACGCTGGGTTCCGGGGACCCATTGTCTCTGGCAACATT
TGGCCAACCGAGTCATCCAGGAAGACTTACGCTTAGTCCAGGGCCAACAGGAGCGTCTCAAGGGGGGGGCTAAT
GTGTGGAACCAACCCGTGGGCTATGACAAGTTGGGGGTGGCCTATCGCCACTGGCGCAATCAAGTGGAACGCCA
CGGTTCTGACTGGCCTGAATCTCCCGCTGATGAGGGTAGGGAGCCTGCTCTCAATGCGATCGTCACGGGGTCAG
ATGCTCCGATCACGGGTTCTGTGGTTTCCCTGCCGCCGTCCCAGGCTCCTCCCACGGGGCACTGA

MNNSLNVSATLDLANGLRNFWYPVEFSKNLGMADPLGFELFDQCWVLFRDDQGTAACILDECAHRACPLSLGKV
IQGRIQCPYHGWEYDRQGECVHMPSCQAISNPILTLPVMEQGGMIWVWPGTDEPGALPSLAPTLPDNFTLQAEL
VMDLEVEHGLMLENLLDLAHAPFTHTGTFAKGWPVPPFVRFANAATTPWTGHWDPYPIHMTFEPPCFVISTIGL
RGKDCGRHLHQVHACLPRGQGRTRLLYRLALDFGHWLRWVPGTHCLWQHLANRVIQEDLRLVQGQQERLKGGAN
VWNQPVGYDKLGVAYRHWRNQVERHGSDWPESPADEGREPALNAIVTGSDAPITGSVVSLPPSQAPPTGH

Chlamydomonas reinhardtii **Chlorophyllide *a* oxygenase (*Cr*CAO)**

UniProt: Q9ZWM5

ATGCTGCCGGCGAGCCTGCAGCGTAAAGCGGCGGCGGTTGGTGGCCGTGGTCCGACCAACCAGAGCCGTGTGGC
GGTTCGTGTGAGCGCGCAACCGAAAGAGGCGCCGCCGGCGAGCACCCCGATCGTGGAGGATCCGGAAAGCAAGT
TCCGTCGTTACGGTAAACACTTTGGTGGCATTCACAAGCTGAGCATGGACTGGCTGGATAGCGTTCCGCGTGTT
CGTGTGCGTACCAAAGACAGCCGTCAGCTGGACGATATGCTGGAGCTGGCGGTGCTGAACGAACGTCTGGCGGG
TCGTCTGGAGCCGTGGCAGGCGCGTCAAAAGCTGGAATACCTGCGTAAGCGTCGTAAAAACTGGGAGCGTATCT
TCGAATATGTTACCCGTCAAGATGCGGCGGCGACCCTGGCGATGATTGAGGAAGCGAACCGTAAGGTGGAGGAA
AGCCTGAGCGAGGAAGCGCGTGAGAAAACCGCGGTTGGTGACCTGCGTGATCAGCTGGAAAGCCTGCGTGCGCA
AGTGGCGCAGGCGCAAGAGCGTCTGGCGATGACCCAGAGCCGTGTGGAACAGAACCTGCAACGTGTTAACGAGC
TGAAGGCGGAAGCGACCACCCTGGAGCGTATGCGTAAGGCGAGCGACCTGGATATCAAAGAGCGTGAACGTATC
GCGATTAGCACCGTTGCGGCGAAAGGTCCGGCGAGCAGCAGCAGCAGCGCGGCGGCGGTGAGCGCGCCGGCGAC
CAGCGCGACCCTGACCGTTGAGCGTCCGGCGGCGACCACCGTGACCCAAGAAGTTCCGAGCACCAGCTACGGTA
CCCCGGTTGATCGTGCGCCGCGTCGTAGCAAAGCGGCGATCCGTCGTAGCCGTGGCCTGGAGAGCAGCATGGAA

ATTGAGGAAGGTCTGCGTAACTTCTGGTATCCGGCGGAATTTAGCGCGTCTGCCGAAGGACACCCTGGTGCC
GTTCGAGCTGTTTGGCGAACCGTGGGTTATGTTCCGTGATGAGAAGGGTCAGCCGAGCTGCATTCGTGATGAAT
GCGCGCACCGTGGCTGCCCGCTGAGCCTGGGTAAAGTGGTTGAGGGCCAAGTTATGTGCCCGTACCACGGTTGG
GAATTCAACGGTGATGGTGCGTGCACCAAGATGCCGAGCACCCCGTTTTGCCGTAACGTTGGCGTGGCGGCGCT
GCCGTGCGCGGAGAAAGACGGTTTTATCTGGGTGTGGCCGGGTGATGGCCTGCCGGCGGAAACCCTGCCGGACT
TTGCGCAGCCGCCGGAGGGCTTTCTGATCCACGCGGAAATTATGGTTGATGTGCCGGTTGAGCACGGTCTGCTG
ATTGAAAACCTGCTGGATCTGGCGCATGCGCCGTTCACCCACACCAGCACCTTTGCGCGTGGCTGGCCGGTGCC
GGACTTCGTTAAGTTTCACGCGAACAAAGCGCTGAGCGGTTTCTGGGACCCGTACCCGATCGATATGGCGTTTC
AGCCGCCGTGCATGACCCTGAGCACCATCGGTCTGGCGCAACCGGGCAAGATTATGCGTGGTGTTACCGCGAGC
CAGTGCAAAAACCACCTGCACCAACTGCACGTGTGCATGCCGAGCAAGAAAGGTCACACCCGTCTGCTGTATCG
TATGAGCCTGGATTTCCTGCCGTGGATGCGTCACGTGCCGTTTATCGACCGTATTTGGAAGCAGGTGGCGGCGC
AAGTTCTGGGCGAGGATCTGGTGCTGGTTCTGGGTCAGCAAGACCGTATGCTGCGTGGTGGCAGCAACTGGAGC
AACCCGGCGCCGTACGACAAACTGGCGGTGCGTTATCGTCGTTGGCGTAACGGCGTTAACGCGGAAGTGGCGCG
TGTTCGTGCGGGTGAACCGCCGAGCAACCCGGTGGCGATGAGCGCGGGCGAGATGTTCAGCGTTGACGAAGACG
ATATGGATAACAGCAGCGGTGAAAACCTGTATTTTCAGGGTAGCGGCAGCAGC

MLPASLQRKAAAVGGRGPTNQSRVAVRVSAQPKEAPPASTPIVEDPESKFRRYGKHFGGIHKLSMDWLDSVPRV
RVRTKDSRQLDDMLELAVLNERLAGRLEPWQARQKLEYLRKRRKNWERIFEYVTRQDAAATLAMIEEANRKVEE
SLSEEAREKTAVGDLRDQLESLRAQVAQAQERLAMTQSRVEQNLQRVNELKAEATTLERMRKASDLDIKERERI
AISTVAAKGPASSSSSAAAVSAPATSATLTVERPAATTVTQEVPSTSYGTPVDRAPRRSKAAIRRSRGLESSME
IEEGLRNFWYPAEFSARLPKDTLVPFELFGEPWVMFRDEKGQPSCIRDECAHRGCPLSLGKVVEGQVMCPYHGW
EFNGDGACTKMPSTPFCRNVGVAALPCAEKDGFIWVWPGDGLPAETLPDFAQPPEGFLIHAEIMVDVPVEHGLL
IENLLDLAHAPFTHTSTFARGWPVPDFVKFHANKALSGFWDPYPIDMAFQPPCMTLSTIGLAQPGKIMRGVTAS
QCKNHLHQLHVCMPSKKGHTRLLYRMSLDFLPWMRHVPFIDRIWKQVAAQVLGEDLVLVLGQQDRMLRGGSNWS
NPAPYDKLAVRYRRWRNGVNAEVARVRAGEPPSNPVAMSAGEMFSVDEDDMDN

Arabidopsis thaliana **Chlorophyllide *a* oxygenase (*At*CAO):**

UniProt: Q9MBA1

ATGAACGCCGCCGTGTTTAGTCCTTCTGCTTTATCTCTCCCTATCTCCTTCTCTAAAACCCGATCCTCTTTTCT
CTCCAGAAAGAAGGGCGTGAAAGGAGAATTTAGGGTATTTGCTGTGTTTGGTGATGAGAGTGGATTAGTTGAGA
AGAAGAGTCAATGGAGACCTTTGTTTGATGTGGAGGATCCTAGATCAAAAGCTCCTCCTTATAAAGGAAAGTTT
TTAGATGTTAATCAAGCTATTGAAGTTGCTAGGTTTGATATTCAATACTTGGATTGGCGTGCTCGTCAAGATCT
TCTTACCATTATGATTCTTCATGACAAGGTTGTTGATGTACTTAATCCTCTAGCTCGTGAGTACAAGTCCATCG
GTACAGTGAAGAAAGAACTAGCTGGATTGCAGGAAGAATTATCGAAAGCACACCAACAGGTTCATATATCTGAA
GCAAGGGTTTCGACTGCTTTAGACAAGTTAGCCCACATGGAAGAATTGGTTAATGATAGGTTGTTACCTGGCAG
AGTTGTAACGGAATTAGATAAACCCTCCTCTTCAACCACTGCTTCTGCTGTCGAGTTAGATAGGGAAAAGACAA
ACACGGGTGCGAAAAGCTTGAATGTTTCTGGTCCGGTTCCGCCTTATAGTCCACACTTGAAGAATTTTTGGTAT
CCCGTTGCTTTCACTGCAGATCTCAAGCATGATACAATGGTACCAATTGAATGCTTTGAACAACCATGGGTTAT
CTTTAGGGGTGAAGACGGGAAACCAGGATGTGTACGGAATACATGTGCGCATAGAGCATGTCCTCTTGATCTTG
GCACAGTGAACGAGGGACGTATTCAATGTCCGTACCATGGATGGGAATACTCAACCGATGGAGAATGTAAGAAG
ATGCCGTCTACAAAGTTACTGAAGGTGAAGATCAAATCATTACCTTGTCTTGAACAAGAAGGTATGATCTGGAT
TTGGCCCGGTGATGAGCCACCTGCACCTATACTTCCTTCTTTACAGCCTCCATCAGGGTTTTTAATTCATGCTG

AGCTTGTAATGGACCTCCCGGTGGAACACGGTTTACTTCTAGATAATCTCTTGGATCTTGCTCATGCCCCATTC
ACTCATACATCCACTTTTGCAAAAGGCTGGAGTGTCCCAAGTTTGGTGAAGTTTTTAACACCTACCTCGGGTCT
CCAAGGATACTGGGATCCATATCCAATCGATATGGAATTTAAACCACCGTGTATTGTTTTATCGACAATCGGGA
TATCAAAACCCGGGAAACTAGAAGGCAAAAGCACACAGCAGTGTGCAACACATCTTCATCAACTCCATGTCTGT
TTACCTTCTTCTAAAAACAAGACAAGACTTCTATACCGAATGTCACTAGACTTTGCTCCTATATTAAAGAATCT
TCCATTCATGGAACATCTGTGGAGACATTTCGCTGAACAGGTCTTAAACGAAGATCTACGGCTAGTTTTAGGAC
AACAAGAACGGATGTTAAACGGAGCAAACATATGGAATTTACCAGTTGCTTATGACAAGCTCGGAGTTCGGTAT
AGACTATGGAGGAACGCAGTAGATCGTGGCGATGATAAACTACCTTTCTCCGGCAGCAGCGGCGAAAATCTGTA
CTTCCAAGGCAGCGGTAGCAGC

MNAAVFSPSALSLPISFSKTRSSFLSRKKGVKGEFRVFAVFGDESGLVEKKSQWRPLFDVEDPRSKAPPYKGKF
LDVNQAIEVARFDIQYLDWRARQDLLTIMILHDKVVDVLNPLAREYKSIGTVKKELAGLQEELSKAHQQVHISE
ARVSTALDKLAHMEELVNDRLLPGRVVTELDKPSSSTTASAVELDREKTNTGAKSLNVSGPVPPYSPHLKNFWY
PVAFTADLKHDTMVPIECFEQPWVIFRGEDGKPGCVRNTCAHRACPLDLGTVNEGRIQCPYHGWEYSTDGECKK
MPSTKLLKVKIKSLPCLEQEGMIWIWPGDEPPAPILPSLQPPSGFLIHAELVMDLPVEHGLLLDNLLDLAHAPF
THTSTFAKGWSVPSLVKFLTPTSGLQGYWDPYPIDMEFKPPCIVLSTIGISKPGKLEGKSTQQCATHLHQLHVC
LPSSKNKTRLLYRMSLDFAPILKNLPFMEHLWRHFAEQVLNEDLRLVLGQQERMLNGANIWNLPVAYDKLGVRY
RLWRNAVDRGDDKLPFSG

*Micromonas pusilla* Chlorophyllide *a* oxygenase1 (*Mp*CAO1):

UniProt: C1MQW7

ATGATCCCGTTCGATCTGTTCAACGTCCCGTGGGTCGCGTTCCGCGACCAGGACGGCATGGCGGGGTGCATCAA
GGACGAGTGCGCGCACAGGGCGTGCCCGATATCCCTCGGGAAAGTCGTCGAAGGACGCGTGCAGTGCCCGTACC
ACGGGTGGGAGTACACCTCGGGAGGGGAGTGCAAGAAGATGCCGTCGATAAAAAACCTCCTCCCGAACGTCTAC
GTCGACGCCGCGCCGATCGTGGAGCGCGACGGGCTGTTATACGTGTGGGCGGGGGTGTGGGAGCCGGAGAGGGC
GGAGGAGATATTGAGCGAGCTCCCGCCGAGCGCGGCGACCGCGCCGCCGAGCGGGTTCGCCGCGATGGCTGAGG
TCACCGTCGACGTCCCGCTCGACGCGCCCGCGATTTTGTCGCGACTGATGGACGAGAACAAGGTGCCGTTCACG
CGCGTGGACACGACGACGCTGAGCGACGACGTCTTCCCGAAGGTGATCGCGAAGGTGTTGCGGGGGTTCGGTAA
GCCCGCGCCGAAACGCGTGGAGTTCACCCCGGCGTGCATACTCGACAGCACGATCGGTCTGGACGGCGTGGGCG
GACAAGACTGGAACGTGCATCAGACGCACGTCGTGTTACCCTCGCGGCCCGGGAAGGCGCGGGTGTTGTACCGC
CTCTCCGTGGACTTTGTCGTCGGCGCGGAGATCGCGCGGACCGTGGGCGGGCAGGTGTGGCAGAACCTCGCGGA
GATGATCCTCCAAGAGCAGCTCGAGGGGATCCGGGGGGGGAGGTTCGAGGACGACAGCGTCGGGGAGCAGGCCG
CGGACGTGTCGCAGAGCTACGACGAGTGGATGGAAGAGATCCAGGCGCCGCGGTGA

MIPFDLFNVPWVAFRDQDGMAGCIKDECAHRACPISLGKVVEGRVQCPYHGWEYTSGGECKKMPSIKNLLPNVY
VDAAPIVERDGLLYVWAGVWEPERAEEILSELPPSAATAPPSGFAAMAEVTVDVPLDAPAILSRLMDENKVPFT
RVDTTTLSDDVFPKVIAKVLRGFGKPAPKRVEFTPACILDSTIGLDGVGGQDWNVHQTHVVLPSRPGKARVLYR
LSVDFVVGAEIARTVGGQVWQNLAEMILQEQLEGIRGGRFEDDSVGEQAADVSQSYDEWMEEIQAPR

*Micromonas pusilla* Chlorophyllide *a* oxygenase2 (*Mp*CAO2):

UniProt: C1MZ04

ATGGCGCCCGAGGTGTCCTCCTCCCCCGCGCCCGTGGACGCCTCGAGGGGGGGCGCTGAGAAAGCCGCGCGCAG
GCTCGGCATGGGACGAAGGTTCGCGGACGAGGTCTCCCTGGACAACCCCTCCGACGTCGGCGCGATCCGCAACT
ATTGGTACCCGATCCACTTCATCTCGAAGCTGAACAAGGGCGACGCGGCCACCTCCTTCGTGCTCTTCGGCGAG
CGGTGGGAGCTCGTCGCCGACGACGACGCCGCCGTCGCCGCCGCGAAGACCGCCGTCGGCGTCTTCGGCCCGGA
GTACGCGGAGACGCAGGCGCACCTGGTGGACGGCGCCGCACAGAGGTGGACGTGCCGCTCGCGCGACGACGCGA
CGCGCTTCCTCCCGATCGGGCTGCAGGACGGCCTCGTCATGCCCGACGTCGCGTTGCCGACGACGTTTACACCC
CCGGCGGGGTACACGACGCACGCGGAGCTCATCATCGAGGACGTCCCCGTGGAGCACGGGCTGCTGATGGAGAA
CCTGCTGGACCTCGCGCACGCGCCGTTCACGCACACCGGGACGTTCGCGAAGGGCTGGGGCGTCCCCACGTTCG
TCGAGTTCGTCACGTCGAAGCTCCGGAGGGAAGGGGACGGGTGGCAGGACATGGCGCGAGGGCTCACGCGCGAG
GGGATCGGGCTGGGGTCGCAGCAGGGGTCGTGGAACCCGTACCCGATCGACATGAAGTTCGTCACGCCGTGCAT
GGTGGACTCGCACATCGGGATGTCGCAGGCTGGCGCCGCGGGGAAGGGGGCGCAGTTTGAAGAGGGCGTCCAGT
GCGCGGAGTGCAGCAACCACCTCCACCAACTCCACGTGTGCGTGCCGTCCGAGCCCGGGCGGACGCGCCTGCTG
TATCGGATGGCGCTCGATTTCGCCGGGTGGGCGAAGTACGTCCCGGGGATCGAGCTCGTGTGGACGGAGATGGC
GAACCAGGTCTTGGGCGAGGACTTGAGGCTGGTGACCGGGCAGCAGGATCGGATGCGGAGAGGCGGACGGGTGT
GGGCGCATCCGGTGGCGTACGATAAGCTCGGGCTGGTGTACCGCAGGTGGAGGAACTTCAGCGTTGGCGAGGCG
TGCGACGTGAGCGCGGGTATCGGCGCGGAGGGCGCGGGGGAGACGGCGCAGCGGTCGCGGTGA

MAPEVSSSPAPVDASRGGAEKAARRLGMGRRFADEVSLDNPSDVGAIRNYWYPIHFISKLNKGDAATSFVLFGE
RWELVADDDAAVAAAKTAVGVFGPEYAETQAHLVDGAAQRWTCRSRDDATRFLPIGLQDGLVMPDVALPTTFTP
PAGYTTHAELIIEDVPVEHGLLMENLLDLAHAPFTHTGTFAKGWGVPTFVEFVTSKLRREGDGWQDMARGLTRE
GIGLGSQQGSWNPYPIDMKFVTPCMVDSHIGMSQAGAAGKGAQFEEGVQCAECSNHLHQLHVCVPSEPGRTRLL
YRMALDFAGWAKYVPGIELVWTEMANQVLGEDLRLVTGQQDRMRRGGRVWAHPVAYDKLGLVYRRWRNFSVGEA
CDVSAGIGAEGAGETAQRSR

***Pseudomonas* VanB**

UniProt: Q9HUQ8

ATGTACTTCCAATCCAATGCAATGATCGAGGTTTTGGTGGCAGCGCGCCACCACGAGGCGCTGGATATTTGCGG
CTACGAACTGACTGCTGTTGACGGTCAGCCACTGCCGGCATTTACGGCGGGTGCACACATTGATGTCCACCTTC
CTACTGGGATGATTCGCCAATATTCTTTATGCAATCACCCTGAGGAACGCCATCGCTACCTTATTGGTGTTCTG
CGTGACCCCGCATCCCGTGGTGGGTCGCGCGCGATGCACGAATTGATCGAGCCTGGTACACGTTTGCAAATCTC
AGAGCCGCGCAATCTTTTTCCTTTGGCCCCTGAGGCTCAACGTTCACTGCTTTTTGCGGGAGGGATCGGGATCA
CCCCGATCCTTTCAATGGCCGAGTTTCTGGCCCAAGACGGTGCCGCTTTCGAGCTGCACTACTGTGCGCGTTCC
CGTGAGCGCGCAGCTTTCGTAGAACGTTTGCGTTTGTCACCTTACGCGGACCGTGTCTTCCTTCATTTCGATGA
AGAGCCGGACACCTTGCTGGACGCCGCTGCAGTCCTGTGCGCGCCAACTGATGATCTTCATTTATATGTCTGCG
GACCCGGCGGATTCATGCAATATATTTTAGATACGGCGCGCCATCATGGGTGGGAAGAGACCCGCCTTCACCGT
GAGTATTTTTCCGCAGCGCCAGTTGATACGCGTGCGGATGGTTCTTTCAGTGTAAAGCTGGCGCGCTCGGGGCA
GGTTTTCGACATCCCGGCAGATCGCTCTGTAGTCCAAGTGTTGGAATCACACGGGATCGAAATTCCAATTAGTT
GTGAGCAGGGCATCTGTGGAACCTGCTTGACCCGCGTCTTGGAAGGGGTCCCAGAACATCGCGATATGTTTTTA
ACTGAAGCAGAGCAGGCGTGTAATGATCAATTCACGCCCTGTTGCTCACGCTCAAAGACGCCTCTTCTGGTCCT
TGACCTGTAGCATTGGAAGTGGATAA

MIEVIVGAIRLEAQDIHSFELFRADGAALPSFEPGAHIDLHLPNGLVRQYSLCGPAERPRHYRIAVLRCRDSRG
GSATLHAELRVGQRLHIGEPRNLFPLSPEPGPHLLFAGGIGITPLLAMAERLARDGADFQLHYCAHSGERAAFV
DYLGRCAFADRVHCHFDHGESSRRADLRALLATSPRDAQLYLCGPAGFMQWIEESARELGWEASRLHREHFAAA
PRDASADGTFEVQLASNGALIRVAAGQTVLAALREAGVDLPASCEQGICGTCLTRVLDGEPEHRDLYLSEEEQA
ANDCFTPCCSRSRSPRLVLDL

Stenotrophomonas maltophilia Dicamba monooxygenase reductase, ferredoxin component (DdmB)

UniProt: Q5S3I4

ATGCCGCAGATTACCGTCGTCAACCAGTCGGGTGAAGAATCCAGCGTCGAGGCGAGTGAAGGCCGCACCCTGATGGA
AGTCATCCGCGACAGCGGTTTTGACGAACTCCTGGCGCTTTGCGGCGGCTGCTGCTCGTGCGCGACCTGCCACGTCC
ACATCGACCCGGCCTTCATGGACAAGCTGCCGGAGATGAGCGAAGACGAGAACGACCTGCTCGACAGCTCGGACCAC
CGCAACGAGTACTCGCGTCTCTCGTGCCAGATTCCGGTCACCGGCGCCCTCGAAGGCATCAAGGTGACGATCGCGCA
GGAAGACTGA

MPQITVVNQSGEESSVEASEGRTLMEVIRDSGFDELLALCGGCCSCATCHVHIDPAFMDKLPEMSEDENDLLDSSDH
RNEYSRLSCQIPVTGALEGIKVTIAQED

Stenotrophomonas maltophilia Dicamba monooxygenase reductase (DdmA)

UniProt: Q5S3I2

ATGAGCAAGGCAGACGTCGTAATCGTGGGAGCCGGGCATGGCGGCGCACAGTGCGCGATCGCCCTTCGCCAGAACGG
CTTCGAAGGAACCATCACCGTCATCGGTCGTGAGCCGGAATATCCCTATGAGCGTCCGCCGCTCTCGAAGGAATATT
TCGCGCGCGAGAAGACCTTCGACCGCCTCTACATCCGTCCGCCGACGTTCTGGGCCGAGAAGAACATCGAGTTCAAG
CTTGGCACCGAAGTCACCAAGGTCGATCCCAAGGCGCACGAACTGACGCTCTCCAACGGCGAGAGCTACGGTTATGG
CAAGCTCGTCTGGGCCACCGGCGGCGATCCGCGTCGCCTTTCTTGCCAGGGGGCCGACCTCACCGGCATCCACGCCG
TGCGCACCCGCGAGGACTGCGACACGCTGATGGCCGAAGTCGATGCGGGCACGAAGAACATCGTCGTCATCGGCGGC
GGCTACATCGGTCTGGAAGCCGCTGCGGTGCTGTCCAAGATGGGCCTCAAGGTCACCCTGCTCGAAGCGCTTCCGCG
CGTGCTGGCGCGCGTTGCGGGTGAAGACCTCTCGACCTTCTACCAGAAGGAACATGTCGATCACGGCGTCGACCTGC
GCACCGAAGTCATGGTCGACAGCCTCGTCGGCGAAAACGGCAAGGTCACCGGCGTGCAGCTTGCCGGCGGCGAAGTG
ATCCCGGCCGAAGGCGTCATCGTCGGCATCGGCATCGTGCCTGCCGTCGGTCCGCTGATCGCGGCCGGCGCGGCCGG
TGCCAACGGCGTCGACGTGGACGAGTACTGCCGCACCTCGCTGCCCGACATCTATGCGATCGGCGACTGTGCGGCTT
TCGCCTGCGACTACGCCGGCGGCAACGTGATGCGCGTGGAATCGGTCCAGAACGCCAACGACATGGGCACCTGCGTG
GCCAAGGCGATCTGCGGCGACGAGAAGCCCTACAAGGCGTTCCCGTGGTTCTGGTCCAACCAGTACGACCTCAAGCT
GCAGACCGCCGGCATCAACCTGGGCTTCGACAAGACCGTGATCCGCGGCAATCCGGAGGAGCGCAGCTTCTCGGTCG
TCTATCTCAAGGACGGCCGCGTGGTCGCGCTGGACTGCGTGAACATGGTCAAGGATTACGTGCAGGGCCGCAAGCTG
GTCGAAGCCGGGGCCACCCCCGACCTCGAAGCGCTGGCCGATGCCGGCAAGCCGCTCAAGGAACTGCTCTAG

MSKADVVIVGAGHGGAQCAIALRQNGFEGTITVIGREPEYPYERPPLSKEYFAREKTFDRLYIRPPTFWAEKNIEFK
LGTEVTKVDPKAHELTLSNGESYGYGKLVWATGGDPRRLSCQGADLTGIHAVRTREDCDTLMAEVDAGTKNIVVIGG
GYIGLEAAAVLSKMGLKVTLLEALPRVLARVAGEDLSTFYQKEHVDHGVDLRTEVMVDSLVGENGKVTGVQLAGGEV

IPAEGVIVGIGIVPAVGPLIAAGAAGANGVDVDEYCRTSLPDIYAIGDCAAFACDYAGGNVMRVESVQNANDMGTCV
AKAICGDEKPYKAFPWFWSNQYDLKLQTAGINLGFDKTVIRGNPEERSFSVVYLKDGRVVALDCVNMVKDYVQGRKL
VEAGATPDLEALADAGKPLKELL

***Comamonas testosteroni* TsaB**

UniProt: P94680

ATGAGCGCCGATGTGCCCGTGACCGTGGCCGCCGTGCGGGCGGTGGCCCGCGACGTGCTGGCGCTGGAACTGCGCCA
CGCCAACGGCCAGCCCCTGCCCGGCGCCAGCGCCGGTGCCCACATTGACCTCGCCCTGCCCAATGGCCTGGTGCGCC
AGTACTCGCTGGTGAACGCCACCGGCCAGGCCACCATGGACTGCTACCAGGTGGCCGTGGGCTGGGACGCCAACAGC
CGTGGCGGCTCGGTGTGGATTCACGAAAAGCTCAAGGTGGGCCAGGCCCTGCGCGTGACGCACCGCGCAACCTGTTC
CGAGATGGCGCCCGAGCACCGCCGCGTGCTGCTGCTGGCCGGTGGCATTGGCGTCACGCCCATCTACGCCATGGCCC
AGGCTTGCGCGCAGCAAGGCGTGGACGTTGAGCTGTGGGCCAGCGCCCGCTCGGCCCCGCGCCTGGCCTACTTGGAA
GAACTCAAGGCACTGCTGGGCCAGCGCCTGCACCTGCATGCCGACGACGAGCAGGGCGGCCCCATGAACCTCACCGA
GCGCCTGGCCACCCAGCGCTGGGACGCGGTTTACGCCTGCGGCCCCGCGCCCATGCTGGACGCGCTCACCGCCGCCA
CCGCCCACTGGGCGCCGGGCTCGGTGCGCATGGAACGCTTCAAGGGCGCCGAGCAGCCCGCCAGCGAGCGCCAGCCC
TTTGAGCTGGTGCTGCAGCGCGCCGGCCTGAGCACCACGGTGGACGCGCACGAGAGCGTGCTCGACGCCATGGAGCG
CGTGGGCGTGGACTTCCCTTGGTCGTGCCGCGAAGGCATTTGCGGCACCTGCGAAGCGCCGGTGCTCGAAGGCGAGG
TGCAGCACCTCGATTACGTGCTCTCGCCCGAAGAACGCGCCGAACAGCGGCGAATGATGGTCTGCGTGTCGCGTTGT
GGCGGCGGCCGGCTGGTCCTGGACATCTGA

MSADVPVTVAAVRAVARDVLALELRHANGQPLPGASAGAHIDLALPNGLVRQYSLVNATGQATMDCYQVAVGWDANS
RGGSVWIHEKLKVGQALRVTHRATCSEMAPEHRRVLLLAGGIGVTPIYAMAQACAQQGVDVELWASARSAPRLAYLE
ELKALLGQRLHLHADDEQGGPMNLTERLATQRWDAVYACGPAPMLDALTAATAHWAPGSVRMERFKGAEQPASERQP
FELVLQRAGLSTTVDAHESVLDAMERVGVDFPWSCREGICGTCEAPVLEGEVQHLDYVLSPEERAEQRRMMVCVSRC
GGGRLVLDI

***Escherichia coli* Flavodoxin reductase**

UniProt: P28861

ATGGCTGATTGGGTAACAGGCAAAGTCACTAAAGTGCAGAACTGGACCGACGCCCTGTTTAGTCTCACCGTTCACGC
CCCCGTGCTTCCGTTTACCGCCGGGCAATTTACCAAGCTTGGCCTTGAAATCGACGGCGAACGCGTCCAGCGCGCCT
ACTCCTATGTAAACTCGCCCGATAATCCCGATCTGGAGTTTTACCTGGTCACCGTCCCCGATGGCAAATTAAGCCCA
CGACTGGCGGCACTGAAACCAGGCGATGAAGTGCAGGTGGTTAGCGAAGCGGCAGGATTCTTTGTGCTCGATGAAGT
GCCGCACTGCGAAACGCTATGGATGCTGGCAACCGGTACAGCGATTGGCCCTTATTTATCGATTCTGCAACTAGGTA
AAGATTTAGATCGCTTCAAAAATCTGGTCCTGGTGCACGCCGCACGTTATGCCGCCGACTTAAGCTATTTGCCACTG
ATGCAGGAACTGGAAAAACGCTACGAAGGAAAACTGCGCATTCAGACGGTGGTCAGTCGGGAAACGGCAGCGGGGTC
GCTCACCGGACGGATACCGGCATTAATTGAAAGTGGGGAACTGGAAAGCACGATTGGCCTGCCGATGAATAAAGAAA
CCAGCCATGTGATGCTGTGCGGCAATCCACAGATGGTGCGCGATACACAACAGTTGCTGAAAGAGACCCGGCAGATG
ACGAAACATTTACGTCGCCGACCGGGCCATATGACAGCGGAGCATTACTGGTAA

MADWVTGKVTKVQNWTDALFSLTVHAPVLPFTAGQFTKLGLEIDGERVQRAYSYVNSPDNPDLEFYLVTVPDGKLSP
RLAALKPGDEVQVVSEAAGFFVLDEVPHCETLWMLATGTAIGPYLSILQLGKDLDRFKNLVLVHAARYAADLSYLPL

MQELEKRYEGKLRIQTVVSRETAAGSLTGRIPALIESGELESTIGLPMNKETSHVMLCGNPQMVRDTQQLLKETRQM
TKHLRRRPGHMTAEHYW

Escherichia coli **Flavodoxin**

UniProt: P61950

ATGGCTATCACTGGCATCTTTTTCGGCAGCGACACCGGTAATACCGAAAATATCGCAAAAATGATTCAAAAACAGCT
TGGTAAAGACGTTGCCGATGTCCATGACATTGCAAAAAGCAGCAAAGAAGATCTGGAAGCTTATGACATTCTGCTGC
TGGGCATCCCAACCTGGTATTACGGCGAAGCGCAGTGTGACTGGGATGACTTCTTCCCGACTCTCGAAGAGATTGAT
TTCAATGGCAAACTGGTTGCGCTGTTTGGTTGTGGTGACCAGGAAGATTACGCCGAATATTTCTGCGACGCATTGGG
CACCATCCGCGACATCATTGAACCGCGCGGTGCAACCATCGTTGGTCACTGGCCAACTGCGGGCTATCATTTCGAAG
CATCAAAAGGTCTGGCAGATGACGACCACTTTGTCGGTCTGGCTATCGACGAAGACCGTCAGCCGGAACTGACCGCT
GAACGTGTAGAAAAATGGGTTAAACAGATTTCTGAAGAGTTGCATCTCGACGAAATTCTCAATGCC

MAITGIFFGSDTGNTENIAKMIQKQLGKDVADVHDIAKSSKEDLEAYDILLLGIPTWYYGEAQCDWDDFFPTLEEID
FNGKLVALFGCGDQEDYAEYFCDALGTIRDIIEPRGATIVGHWPTAGYHFEASKGLADDDHFVGLAIDEDRQPELTA
ERVEKWVKQISEELHLDEILNA

Triticum aestivum **Chlorophyllase**

UniProt: A0A3B6QC98

ATGGCTAGCGCAGCAGCAGCACCCGCAGAGACGATGAACAAGTCCGCCGCCGGCGCCGAGGTTCCCGAGGCGTT
CACATCGGTGTTCCAGCCGGGGAAGCTTGCGGTCGAGGCGATTCAGGTGGATGAGAATGCGGCGCCGACACCAC
CGATCCCGGTGCTGATCGTCGCACCCAAGGATGCAGGAACCTACCCCGTGGCCATGCTCTTGCACGGCTTCTTC
CTCCATAACCACTTCTACGAACACCTTCTCCGGCACGTCGCATCCCACGGCTTCATCATTGTCGCGCCCCAGTT
CAGCATCAGTATCATACCTTCGGGTGACGCAGAGGACATCGCCGCGGCAGCCAAGGTGGCAGACTGGCTCCCCG
ACGGCCTCCCGTCCGTGCTGCCCAAAGGCGTCGAGCCGGAGCTCTCGAAGCTCGCCTTGGCCGGCCACAGCCGA
GGAGGCCACACGGCTTTCTCCCTGGCCTTGGGGCACGCCAAGACCCAGCTAACCTTCTCCGCGCTCATCGGACT
CGACCCCGTCGCCGGCACGGGGAAGTCCTCCCAGCTCCAGCCCAAGATCCTCACCTACGAGCCGTCCTCCTTCG
GCATGGCGATGCCGGTGCTGGTCATCGGCACCGGGCTCGGCGAGGAGAAGAAGAACATATTCTTCCCTCCCTGC
GCACCCAAGGACGTGAACCACGCCGAGTTCTACCGCGAGTGCAGGCCGCCCTGCTACTACCTTGTGACCAAGGA
CTACGGGCATCTGGACATGCTGGACGACGACGCCCCCAAGTTCATCACCTGCGTCTGCAAGGATGGGAACGGGT
GCAAGGGCAAGATACGGAGGTGCGTTGCTGGGATCATGGTGGCATTTCTTAATGCTGCCTTGGGTGAGAAAGAT
GCAGATCTTGAGGCCATACTGAGAGACCCGGCGGTTGCACCCACCACGCTTGATCCGGTTGAGCACCGCGTGGC
GGCGGCCGCACTCGAG

MASAAAAPAETMNKSAAGAEVPEAFTSVFQPGKLAVEAIQVDENAAPTPPIPVLIVAPKDAGTYPVAMLLHGFF
LHNHFYEHLLRHVASHGFIIVAPQFSISIIPSGDAEDIAAAAKVADWLPDGLPSVLPKGVEPELSKLALAGHSR
GGHTAFSLALGHAKTQLTFSALIGLDPVAGTGKSSQLQPKILTYEPSSFGMAMPVLVIGTGLGEEKKNIFFPPC
APKDVNHAEFYRECRPPCYYLVTKDYGHLDMLDDDAPKFITCVCKDGNGCKGKIRRCVAGIMVAFLNAALGEKD
ADLEAILRDPAVAPTTLDPVEHRVAAAALE

IIb. Protein production and purification

Protein Expression Protocols *Ph*CAO

Small-scale expression tests on an N-terminal 6X-His-tagged construct of *Ph*CAO in different *E. coli* strains (BL21(DE3), C41(DE3) and Δ*iscR* Bl21-(DE3)) revealed no significant overexpression under different levels of Isopropyl β-D-1-thiogalactopyranoside or at different temperatures. A C-terminal 6X-His-tagged construct was subsequently tested and shown to yield better overexpression of the target *Ph*CAO protein in the Δ*iscR* Bl21-(DE3) cell line. However, analysis by gel filtration chromatography suggested that this purified protein existed predominantly as an aggregate. Thus, a new codon optimized construct was cloned into the pMCSG9 vector (Genscript). This construct contains an N-terminal His-MBP tag and was tested for expression in the *E. coli* strains listed above. The pMCSG9 plasmid containing *Ph*CAO was transformed by standard heat-shock protocols into C41(DE3) chemically competent *E. coli* cells, which showed the highest level of over-expression. A 5 mL starter was grown from one colony in Luria Broth (LB) containing 50 μg/mL ampicillin in a constant temperature incubator at 37°C and 200 rpm. After an overnight incubation, the starter culture was used to inoculate larger 1 L Terrific Broth (TB) cultures containing 50 μg/mL ampicillin. These 1 L cultures were grown at 37°C and shaken at 200 rpm until the optical density at 600 nm (OD_{600}) reached a value of approximately 0.8-1.0. At this point, flasks were incubated at 20°C, left for 2 hours, and induced by the addition of 0.2 mM isopropyl-β-D-1-thiogalactopyranoside (IPTG), 0.2 mg/mL ferric ammonium citrate, and 0.4 mg/mL ferrous sulfate heptahydrate. The temperature of the incubator was then decreased to 18°C and left for an additional 16 hours before harvesting.

Protein Expression Protocols for His-tagged *At*CAO and His-tagged *Cr*CAO

As described for *Ph*CAO, both *At*CAO and *Cr*CAO were codon optimized, synthesized, and cloned into pET-41b(+) plasmids by Genscript. These *At*CAO and *Cr*CAO plasmids were transformed by standard heat shock protocols into ΔIscR chemically competent cells and plated on LB plates containing 50 μg/mL kanamycin. 5 mL starter cultures were grown and used to inoculate 1 L TB cultures containing 50 μg/mL kanamycin. Expression of both *At*CAO and *Cr*CAO was induced as described for *Ph*CAO.

Protein Expression Protocols for *Mp*CAO1 and *Mp*CAO2

A pRSFDuet-1 plasmid containing codon optimized *Mp*CAO1 and *Mp*CAO2 genes was transformed by standard heat-shock protocols into C41(DE3) chemically competent cells. Starters were grown as described for *Ph*CAO and subsequently used to inoculate 1 L cultures of LB containing 50 μg/mL kanamycin. Cultures were grown at 37°C, 200 rpm until the OD_{600} reached 0.6-0.8. Following the addition of 0.2 mM isopropyl-β-D-1-thiogalactopyranoside (IPTG), Flasks were then incubated at 20°C for 16 hours before harvesting.

Protein Expression Conditions for the reductase proteins VanB, DdmB, DdmA, TsaB, and *E. coli* flavodoxin (Flv) and *E. coli* flavodoxin (Flx) reductase.

The methods for purifying VanB, DdmB, DdmA, and *E. coli* Flv and Flx were previously described and were followed here[3-5]. In brief, these methods require a pMCSG7-*vanB,* pMCSG7-*ddmB,* pMCSG7-*ddmA,* pET28a-*flv,* and pET28a-*flx* plasmids, as well as BL21(DE3) (VanB, Flv,

and Flx) and C41(DE3) (DdmB and DdmA) *E. coli* cells. TsaB, on the other hand, was codon optimized, synthesized, and cloned into a pet-28a(+)-TEV plasmid by Genscript. This pET28a(+)-TEV-*tsaB* plasmid was transformed into BL21(DE3) *E. coli* cells. TsaB was then grown and expressed in cultures of TB containing 50 µg/mL kanamycin until the OD_{600} reached 0.6-0.8. At this point, expression was induced with 0.1 mM IPTG. These cultures were then left at 20°C for 16 hours.

Protein Expression Protocols for *T. aestivum* Chlorophyllase

A pET21d plasmid containing an N-terminal 6X-His-tagged *T. aestivum* chlorophyllase was synthesized and codon-optimized for *E. coli* expression by Genscript. However, this initial plasmid did not result in overexpression of the chlorophyllase protein. Thus, the codon-optimized chlorophyllase gene was subcloned into a pET21d plasmid that contained a C-terminal 6X-His-tag. This new construct was transformed into BL21(DE3) *E. coli* competent cells. To produce the chlorophyllase protein, a single colony of the transformant was grown in LB medium that contained 50 µg/mL of ampicillin at 37°C. When the OD_{600} of this culture reached 0.8, protein expression was induced with 0.5 mM IPTG for 16 hours at 16°C.

Protein Purification Protocols for His-MBP-tagged *Ph*CAO

The cell pellet produced from 1 L of culture that contained the over-expressed His-MBP-tagged *Ph*CAO was resuspended in 50 mL of lysis Buffer (50 mM Tris-HCl (pH 7.5), 200 mM NaCl, and 5% glycerol). Resuspended cells were lysed by sonication and clarified by centrifugation in an Eppendorf centrifuge 5810R at 12,000 x g for 40 min. The supernatant, which contained the MBP-tagged protein *Ph*CAO, was loaded onto 5 mL MBP-Trap column (Cytiva). This column was washed with 10 column volumes of Buffer A (50 mM Tris-HCl (pH 7.5), 200 mM NaCl, and 5% glycerol) and eluted with a five-column volume gradient of Buffer B (50 mM Tris-HCl (pH 7.5), 200 mM NaCl, 5% glycerol, and 10 mM maltose). Fractions that bound to the column which contained the MBP-*Ph*CAO were pooled and dialyzed into Buffer C (20 mM Tris-HCl (pH 7.5), 50 mM NaCl, 1 mM DTT, and 10% glycerol). Following buffer exchange, the His-MBP tag from the *Ph*CAO protein was cleaved using 5 mg of Tobacco Etch Virus (TEV) protease. Following a 20 h incubation at 4°C, the tag cleavage reaction was complete. The tag-free protein was loaded onto a 5 mL His-Trap (Cytiva) for further purification and removal of TEV protease and the cleaved His-MBP tag. The flow-through from the column, which contained the desired tag-free protein was concentrated and loaded onto a HiPrep 16/60 Sephacryl S200-HR (Cytiva) gel filtration column that was pre-equilibrated in Buffer D (50 mM HEPES (pH 8.0), 200 mM NaCl, and 10% glycerol).

Protein Purification Protocols for *At*CAO and *Cr*CAO

The cell pellet produced from a 1 L of culture that contained the over-expressed 6X-His-tagged *At*CAO or *Cr*CAO was resuspended in Buffer A (50 mM Tris-HCl (pH 7.5), 200 mM NaCl, 10 mM imidazole, and 5% glycerol). Cells were lysed by sonication, centrifuged, and loaded onto 5 mL His-Trap column. This column was washed with 10 column volumes of Buffer A and eluted with a five-column volume gradient of Buffer B (50 mM Tris-HCl (pH 7.5), 200 mM NaCl, 200 mM

imidazole, and 5% glycerol). Fractions containing the *At*CAO or *Cr*CAO were pooled and exchanged into storage Buffer (50 mM HEPES (pH 8.0), 200 mM NaCl, and 10% glycerol) using a PD-10 desalting column (BioRad) to remove imidazole.

Protein Purification Protocols for *Mp*CAO1 and *Mp*CAO2

The cell pellet produced from a 2 L culture of *Mp*CAO1 and *Mp*CAO2 was resuspended in 50 mL lysis Buffer (50 mM HEPES (pH 7.5), 250 mM NaCl, and 5% glycerol). Cells were lysed by sonication and centrifuged. The supernatant, which contained the His-tagged proteins *Mp*CAO1 and *Mp*CAO2 was loaded onto a 5 mL His-Trap column. This column was performed, and the protein was stored as described for *At*CAO and *Cr*CAO.

Purification protocol for the reductase proteins VanB, DdmB, DdmA, TsaB, and *E. coli* Flv and Flx

The methods for purifying VanB, DdmB, DdmA, were previously described and were followed here[3-4]. In short, the cells from 2 L cultures were resuspended in 50 mL of Buffer A (50 mM Tris-HCl pH 8.0, 200 mM NaCl, 20 mM imidazole, and 10% glycerol). These proteins were then lysed, centrifuged, and purified using nickel affinity and gel filtration chromatography. *E. coli* Flv and Flx were purified using a similar protocol with small deviations. Specifically, the Flv and Flx buffers, as previously described[5], contained 20 mM Tris–HCl (pH 8.5), 1 M NaCl, 10 μM FMN (Flv) or 10 μM FAD (Flx), and either 20 mM or 200 mM imidazole. Purified proteins were concentrated to approximately 200 μM and flash frozen by liquid nitrogen and stored at -80°C.

Protein Purification Protocol for *T. aestivum* Chlorophyllase

The cell pellet harvested after overexpressing chlorophyllase was resuspended in Buffer A (50 mM Tris-HCl (pH 7.5), 100 mM NaCl and 5 mM imidazole). The cells were lysed by sonication, centrifuged, and loaded onto a 5 mL Ni-NTA column. Once the supernatant was all loaded, Buffer A was used to wash the column for 10 column volumes. C-terminal His-tagged Chlorophyllase was eluted using Buffer B which contained 50 mM Tris-HCl pH 7.5, 100 mM NaCl and 200 mM imidazole. The purified Chlorophyllase was concentrated to 5 ml and was loaded onto a Superdex 200 size exclusion column that was pre-equilibrated with Buffer C containing 50 mM HEPES pH 7.5 and 100 mM NaCl. Chlorophyllase was eluted as a dimer in the size exclusion column with Buffer C. Chlorophyllase was concentrated to around 60 μM and flash frozen using liquid nitrogen for long-term storage in -80°C freezer.

Caution! *Liquid nitrogen is hazardous and may cause cryogenic burns or injury. It was handled with correct protection precautions in minimal amounts.*

IIc. Preparation of Substrates and Product Standards

Chlorophyllase reaction to produce Chlorophyllide species

Purified His-tagged chlorophyllase (Chlase) was stored in the -80°C freezer at a stock concentration of 30 μM. To perform a reaction, 2 μM Chlase was combined with 160 μM of the desired Chlorophyll molecule in DMSO (Chl *a,* Chl *b,* 7-OH-Chl *a,* and Bchl *a*), and 50 mM Tris-

HCl (pH 7.2). Reactions were initiated by addition of a Chl molecule and incubated at 30°C for 2 h in the dark. Reactions were quenched by the addition of 150 µL acetonitrile (HPLC-grade). Quenched reactions were centrifuged at 17,000 *x g* for 10 min. 50 µL of the supernatant was diluted with 150 µL acetonitrile containing 0.5 mM 1,3,5-trimethoxy-benzene as internal standard.

Caution! *Acetonitrile is classified as a GHS category 2 flammable liquid. Keep away from open flames and heat. Non nitrile gloves need to be worn when working with this liquid as it can penetrate nitrile gloves.*

Preparation of 7-OH-Chlorophyllide *a*

The preparation of 7-OH-Chlorophyllide *a* was accomplished in two steps. The first step, production of 7-OH-Chlorophyll *a*, was performed using previously described methods[6]. In short, 1 mg of Chlorophyll *b* from spinach (Sigma Aldrich) was dissolved in 5 mL of methanol containing 1.0 mg of $NaBH_4$. The reaction was stirred for 2 min at room temperature and then quenched by the addition of 5 ml solution of saturated NaCl. The reaction products were then transferred into dichloromethane (DCM) and dried by anhydrous sodium sulfate. A rotary evaporator was then used to remove extra solvent. The second step was to convert the produced 7-OH-Chlorophyll *a* into 7-OH-Chlorophyllide *a*. This step was accomplished using purified chlorophyllase and the methods described above.

Caution! *Sodium borohydride (CAS: 16940-66-2) is water reactive and highly flammable. Must be handled under inert gas. All manipulations were performed on smallest practical scale.*

Preparation of Chlorophyllide *a′*

To prepare Chlorophyllide *a′*, we capitalized on a previous method[7] and made small modifications. In short, Chlorophyllide *a’* was prepared by dissolving 1 mg of Chlorophyll *a* (Frontier Scientific) in 5 mL of diethyl ether. To this solution 50 µL of pyridine was added to a final volume of 1%. The reaction was stirred at room temperature for 1.5 hours before removal of the solvent using a rotary evaporator. The resulting liquid containing both diastereomers of Chlorophyllide *a* was then dissolved in 200 µL of DMSO for storage. A second step that employed chlorophyllase and the methods described above was followed to produce Chlorophyllide *a′*.

Caution! *DMSO is a combustible liquid and must be handled using correct protection precautions.*

Preparation of Chlorophyllide *b′*

The preparation of Chlorophyllide *b′* was accomplished using 1 mg of Chlorophyll *b* (Sigma Aldrich). The protocol followed was like that described previously[7], and for Chlorophyllide *a*’. First, Chlorophyll *b* was dissolved in 1 mL of diethyl ether. Then 200 µL of this solution was added into an additional 1.6 mL of diethyl ether and 15% pyridine. The reaction was stirred at room temperature for 14 h before removal of the solvent via using a rotary evaporator. The resulting liquid containing both diastereomers was then dissolved in 200 µL of DMSO for storage. Finally, chlorophyllase was used to complete production of Chlorophyllide *b′*.

Preparation of 3-formyl-Chlorophyllide *a* and 3-formyl-Chlorophyllide *b*

To prepare 3-formyl-Chlorophyllide *a* and 3-formyl-Chlorophyllide *b*, we capitalized on a previous method[2] and made small modifications. Briefly, 1 mM Chlorophyll *a* or Chlorophyll *b* was mixed with 10 µM chlorophyllase and incubated at 30°C for 1 h to produce Chlorophyllide *a*. 5 uL of β-mercaptoethanol (βME) and 200 µM heme chloride were then added into this reaction mixture for another 4-hour incubation at 30°C. The resulting liquid containing a mixture of Chlorophyllide *a* and 3-formyl-Chlorophyllide *a* or a mixture of Chlorophyllide *b* and 3-formyl-Chlorophyllide *b* was then fast-frozen by liquid nitrogen for storage for future assays.

IId. Enzymatic Reactions

CAO reactions in cell lysate

Stocks of each of the CAO homologs were prepared from purified (and frozen) stocks of *Ph*CAO, or His-tagged *At*CAO, *Cr*CAO and *Mp*CAO at a concentration of 100 µM. *A. thaliana*, Spinach, Green Algae, Barely, and *E. coli* cell lysate (GenLysate, GBiosciences) were reconstituted by adding 100 µL MQ water, respectively. Reactions consisting of 20 µM CAO homolog, 10 µL lysate solution, 1 µM Chlase, 100 µM Chl *a,* 500 µM NADPH, 200 µM $Fe(NH_4)_2(SO_4)_2$ and 50 mM Tris-HCl pH 7.2 were mixed and incubated at 30°C for 3 hours in the dark. These reactions were then quenched by the addition of 150 µL acetonitrile. Quenched reactions were centrifuged at 17,000 x *g* for 10 min and 50 µL of the supernatant was diluted with 150 µL acetonitrile containing an internal standard (0.5 mM 1,3,5-trimethoxy-benzene as internal standard). Equivalent reactions were performed with Spinach, Barley, Green Algae, and *E. coli* lysate (GenLysate, GBiosciences).

CAO reactions with non-native reductases TsaB, DdmA/B, VanB, Spinach ferredoxin/ferredoxin reductase and *E. coli* flavodoxin/flavodoxin reductase

Stocks of each of the CAO homologs were prepared from purified (and frozen) stocks of *Ph*CAO, or His-tagged *At*CAO, *Cr*CAO and *Mp*CAO at a concentration of 100 µM. The ability of VanB to reduce the Fe-S cluster of *Ph*CAO was tested by titrating 1.5 equivalents of VanB into a sample that contains 45 µM of *Ph*CAO with 1 mM NADPH at room temperature. The UV-Vis spectrum was measured by NanoDrop (ThermoScientific). Reactions consisting of 20 µM CAO, 40 µM VanB, 1 µM Chlase, 100 µM Chl *a* or 80 µM 7-OH-Chlorophyllide *a,* 500 µM NADPH, 200 µM $Fe(NH_4)_2(SO_4)_2$, and 50 mM Tris-HCl pH 7.2 were mixed and incubated at 30°C in the dark. After 3 h, these reactions were quenched by the addition of 150 µL acetonitrile. Quenched reactions were centrifuged at 17,000 x *g* for 10 min and 50 µL of the supernatant was diluted with 150 µL acetonitrile containing 0.5 mM 1,3,5-trimethoxy-benzene as internal standard. Equivalent reactions were performed with 40 µM of TsaB, 32 µM DdmA/DdmB, 20 µM *E. coli* flavodoxin/60 µM flavodoxin reductase, or 20 µM Spinach ferredoxin/60 µM ferredoxin reductase (Sigma Aldrich). However, it was determined that VanB provided the highest level of activity for the assays. In addition, equivalent reactions were also performed with Chl *a*, Bchl *a* Bchlide *a,* 3-formyl-Chlide *a* (Chlide *d*), and Pheophorbide *a*.

LC-MS and MS/MS analysis

Liquid chromatography-mass spectrometry (LC-MS) analysis was performed on an Agilent G6545A quadrupole-time of flight (Q-TOF) or an Agilent 6230 time of flight (TOF) mass spectrometer equipped with a dual AJS ESI source and an Agilent 1290 Infinity series diode array detector, autosampler, and binary pump. Solvent A contained water with 0.1% formic acid. Solvent B contained 95% acetonitrile, 5% water and 0.1% formic acid. For all separations, a ZORBAX RRHT StableBond Aq (2.1 x 50 mm, 1.8 µm, 80Å) column from Agilent was used. The employed chromatographic method used was made up of three steps: (step i) 40% Solvent B 0-1 min, (step ii) a linear gradient to 100% Solvent B over 1 min, and (step iii) 100% Solvent B for 2 min. 2.0 µL injections were made for each sample and the column was run at 0.4 mL/min.

Steady-state kinetics of *Ph*CAO with 7-OH-Chlorophllide *a*

To determine the linear range for kinetic analysis, duplicate 50 µL reactions with 10 µM *Ph*CAO and 10 µM 7-OH-Chlorophyllide *a* were performed and analyzed compared to an internal standard (15 mM 1,3,5-trimethoxy-benzene) by LC/MS. A 5 min time point was demonstrated to be the optimal time to capture the initial reaction rate.

To determine the steady-state kinetic parameters of *Ph*CAO with a 7-OH-Chlorophyllide *a* substrate*,* reactions were conducted on a 50 µL scale with substrate ranging 0.4 µM – 80 µM in duplicate with 10 µM *Ph*CAO, 15 µM VanB, 500 µM NADPH, 200 µM $Fe(NH_4)_2(SO_4)_2$ and 50 mM Tris-HCl (pH 7.2) buffer. Prior to reaction initiation, 1 mM 7-OH-Chlorophyll *a* was incubated with 5 µM chlorophyllase at 30°C for 2 h to generate the needed substrate, 7-OH-Chlorophyllide *a*. CAO reactions were then initiated by the addition of freshly-made 7-OH-Chlorophyllide *a* and quenched after 5 min by the addition of 150 uL acetonitrile. Quenched reactions were centrifuged at 17,000 x *g* for 10 min and 50 µL of the supernatant was diluted with 150 µL acetonitrile containing 15 mM 1,3,5-trimethoxy-benzene as an internal standard. 2 uL each sample was injected on the Q-TOF LC/MS. The result data points were then plotted and fitted into Michaelis-Menten curve using GraphPad Prism.

III. Supplementary References

1. Kunugi, M.; Takabayashi, A.; Tanaka, A., Evolutionary changes in chlorophyllide a oxygenase (CAO) structure contribute to the acquisition of a new light-harvesting complex in micromonas. *J Biol Chem* **2013,** *288* (27), 19330-41.
2. Loughlin, P. C.; Willows, R. D.; Chen, M., In vitro conversion of vinyl to formyl groups in naturally occurring chlorophylls. *Sci Rep* **2014,** *4*, 6069.
3. Lukowski, A. L.; Liu, J.; Bridwell-Rabb, J.; Narayan, A. R. H., Structural basis for divergent C-H hydroxylation selectivity in two Rieske oxygenases. *Nat Commun* **2020,** *11* (1), 2991.
4. Lukowski, A. L.; Ellinwood, D. C.; Hinze, M. E.; DeLuca, R. J.; Du Bois, J.; Hall, S.; Narayan, A. R. H., C-H Hydroxylation in Paralytic Shellfish Toxin Biosynthesis. *J Am Chem Soc* **2018,** *140* (37), 11863-11869.
5. Lanz, N. D.; Grove, T. L.; Gogonea, C. B.; Lee, K. H.; Krebs, C.; Booker, S. J., RlmN and AtsB as models for the overproduction and characterization of radical SAM proteins. *Methods Enzymol* **2012,** *516*, 125-52.
6. Wang, X.; Liu, L., Crystal Structure and Catalytic Mechanism of 7-Hydroxymethyl Chlorophyll a Reductase. *J Biol Chem* **2016,** *291* (25), 13349-59.
7. Helfrich, M.; Bommer, B.; Oster, U.; Klement, H.; Mayer, K.; Larkum, A. W.; Rudiger, W., Chlorophylls of the c family: absolute configuration and inhibition of NADPH:protochlorophyllide oxidoreductase. *Biochim Biophys Acta* **2003,** *1605* (1-3), 97-103.
